# An *AP2*-Family Gene Correlates with the Double-Flower Trait in *Petunia* × *hybrida*

**DOI:** 10.3390/plants14091314

**Published:** 2025-04-26

**Authors:** Tong Xie, Saneyuki Kawabata

**Affiliations:** Graduate School of Agricultural and Life Sciences, The University of Tokyo, 1-1-1 Midori, Nishitokyo, Tokyo 188-0002, Japan; xielilixt@gmail.com

**Keywords:** *Petunia hybrida*, ABC model, double-flower, floral development, petal number, AP2/ERF, transcription factor

## Abstract

The double-flower trait is highly valued in ornamental plants due to its unique aesthetic appeal, yet its genetic basis varies significantly across different species. While *AGAMOUS* (*AG*) and *APETALA2* (*AP2*)-like genes have been demonstrated to play crucial roles in floral organ identity regulation in the model plant *Arabidopsis thaliana*, the underlying mechanisms governing double-flower formation in many ornamental species remain largely unexplored. In this study, we examined the inheritance pattern of this trait and identified a genetic variant associated with petal number variation. Crosses between the single-flowered cultivar ‘Baccarat White’ (BW) and the semi-double cultivar ‘Duo Lavender’ (DL) produced a 1:1 segregation of single and semi-double flowers in the F1 generation, while self-pollination of DL yielded a 1:2:1 segregation of single, semi-double, and double flowers. These results indicate that the double-flower trait follows a single-gene, semi-dominant inheritance model. Whole-genome sequencing of BW and DL followed by sequence analysis of floral organ identity genes revealed no significant differences in B-class (*PhGLO1*, *PhGLO2*, *PhDEF*, and *PhTM6*) or C-class (*pMADS3* and *FBP6*) genes between the two cultivars. Notably, a 10 kb insertion upstream of the *miR172* target site in the *PhBOB* gene was detected in DL. PCR genotyping of 192 F1 progenies demonstrated complete co-segregation between this insertion and the double-flower phenotype, suggesting a strong genetic association. Moreover, qRT-PCR analysis showed that *PhBOB* expression was significantly elevated in DL—exhibiting a 69-fold increase in petals compared to BW—implying that its overexpression disrupts the petal-to-stamen identity transition. Additionally, another *AP2* family gene, *PhROB3*, was upregulated in semi-double flowers, with a 10-fold higher expression in the petals and stamens of DL relative to BW, suggesting its potential role in floral organ differentiation. This study elucidates the molecular regulatory mechanism underlying the double-flower trait in petunia, highlighting the role of *PhBOB* in floral organ identity specification and providing new insights into the potential function of *PhROB3* in double-flower development.

## 1. Introduction

Petunia (*Petunia* × *hybrida*) is a cherished ornamental plant that originated from crosses among *P. axillaris* and *P. integrifolia*, with a breeding history dating back to the 1830s [1,2,3]. Through extensive artificial selection and genetic improvement, petunia cultivars have developed remarkable diversity in growth habits, flower color, and floral size [3,4]. Its rich genetic background not only provides broad breeding potential but also makes it a staple in horticultural landscaping and urban greening [5].

The double-flower trait is a desirable ornamental feature that enhances petal number and overall floral aesthetics. In complete flowers, floral organs are arranged sequentially from the outermost to the innermost layers as sepals, petals, stamens, and carpels. This arrangement is traditionally understood to be governed by the ABC class genes [6,7]. In *Arabidopsis thaliana*, five key homeotic genes grouped into classes A, B, and C have been identified as crucial for establishing the layered structure of the flower. Specifically, class A genes (*APETALA1*, *APETALA2*) specify sepals; the combined activity of A and B class genes (*APETALA3*, *PISTILLATA*) specifies petals; class C gene (*AGAMOUS*) specifies carpels; and classes B and C together specify stamens [8,9,10,11]. A double flower mutant of *A*. *thaliana*, arising from the loss of function of *AG*, exhibits the homeotic transformation of stamens and carpels into petals and sepals, and this trait is recessively inherited [12,13]. Although the double flower trait was generally believed to be caused by the mutation in C class genes, and indeed some double flower plants are due to such mutations, double flower varieties of many ornamental plants, such as lisianthus (*Eustoma grandiflorum*) [14], peach (*Prunus persica*) [15], carnation (*Dianthus caryophyllus* L.) [16,17], rose (*Rosa chinensis*) [16], and Japanese apricot (*Prunus mume*) [18], were found to exhibit dominant inheritance.

Ishimori [14] analyzed double-flowering in *E. grandiflorum*, which exhibits normal development of both stamens and pistils, and found that this trait is dominant. Through whole genome sequencing and RAD marker analysis, he identified the causative gene as *EgAP2d*, a member of the *AP2* family genes. He found a point mutation in the *miR172* target site of double flower *E. grandiflorum*. The mutation at the *miR172* target site was considered to prevent the inactivation of the *EgAP2* gene by *miR172*. Similar mutations near the *miR172* binding site in *AP2*-like genes have been reported to cause the double-flower trait in other species. In *Prunus persica* (peach), the dominant *Di2* locus, which is known to control the double-flower trait, was found to carry the *Prupe.6G242400* gene. This gene encodes an euAP2 transcription factor that contains a deletion at the *miR172* binding site, allowing it to evade *miR172*-mediated degradation [15]. Similarly, in *R*. *chinensis*, an *AP2-like* gene (*RcAP2L*) has been identified as a key regulator of the double-flower trait. A transposon insertion was found within the eighth intron of *RcAP2L*. Further analysis revealed that this mutation was strongly correlated with the double-flower phenotype. The authors considered that abnormal regulation of *RcAP2L* by *miR172* restricted *AGAMOUS* (*RcAG*) function, leading to double-flower formation [16]. In *P. mume*, the *AP2-like* gene *PmAP2L* with a 49 bp deletion at the *miR172* binding site was suggested to play a key role in double-flower formation [18].

The AP2 gene family exhibits distinct regulatory mechanisms compared to other ABC model genes. Unlike B- and C-class genes, which belong to the MADS-box transcription factor family, *AP2* is a member of the AP2/ERF (Ethylene Response Factor) superfamily [19,20]. This gene family is characterized by at least one highly conserved AP2 DNA-binding domain [21,22,23] and is divided into three subfamilies: AP2, EREBP (Ethylene-Responsive Element Binding Proteins), and RAV (Related to ABI3/VP1) [24,25]. Among these, the AP2 subfamily plays a crucial role in floral organ specification and flowering regulation [26], distinguished by its unique structure containing two AP2 domains and a microRNA *miR172* binding site [27,28]. Unlike MADS-box genes, which primarily function as transcription factors, *AP2* genes are subject to post-transcriptional regulation by *miR172*, adding an additional layer of complexity to their functional dynamics. These differences suggest that A-class gene regulation is not a simple hierarchical model as proposed in the ABC theory, but rather a finely tuned process that varies across species.

In petunia, AP2 family genes play a critical role in floral organ identity, exhibiting regulatory patterns distinct from those in *Arabidopsis thaliana*. Based on phylogenetic analysis, petunia AP2 genes can be categorized into two major groups: AP2-type and TARGET OF EAT (TOE)-type [29,30,31]. Several AP2 family genes involved in floral development have been identified in petunia, including *PhROB1* (*REPRESSOR OF B-FUNCTION1*), *PhROB2* (*REPRESSOR OF B-FUNCTION2*), *PhROB3* (*REPRESSOR OF B-FUNCTION3*), *PhBEN* (*BLIND ENHANCER*), and *PhBOB* (*BROTHER OF BEN*). Among these, *PhBEN* functions as a C-class gene repressor, together with a miRNA *BLIND*, ensuring that C-class activity is restricted to the inner floral whorls [32]. Meanwhile, *PhROB1*, *PhROB2*, *PhROB3*, and *PhBEN* collectively repress B-class genes in the first floral whorl without affecting C-class gene function [33,34].

Petunias also exhibit double flowers, which were reported to be dominantly inherited by U et al. in 1930 [35]. However, the gene responsible for the trait remained unknown for a long time. Given that the trait is dominantly inherited, it appeared to result from a mutation in the *AP2* genes. Recently, Gattolin et al. (2020) suggested that a mutation in the gene *BOB* might be responsible for the double flower trait in petunia. However, the gene was not characterized in detail [17].

Despite considerable advances in understanding the roles of ABC-class genes in floral organ development, there remains a gap in our understanding of the contributions of *AP2* family genes to these processes, particularly in the context of ornamental traits. Double-flowered petunias, which exhibit a desirable variation in petal arrangement, serve as an excellent model for investigating the influence of *AP2* family genes on flower morphology.

To deepen our understanding of how these genes contribute to the double flower trait in petunia, we performed whole-genome sequencing and analysis on two commercial cultivars: a single-flower variant, ‘Baccarat White’ and a semi-double flower variant, ‘Duo Lavender’. Our objective was to compare their genomic sequences to identify specific genetic variations associated with the double-flower trait. This comparative analysis aims to clarify the role of *AP2* family genes and potentially pinpoint genetic alterations that lead to the development of double flowers in petunia.

## 2. Results

### 2.1. Inheritance of Double Flower Trait

**Figure 1:**
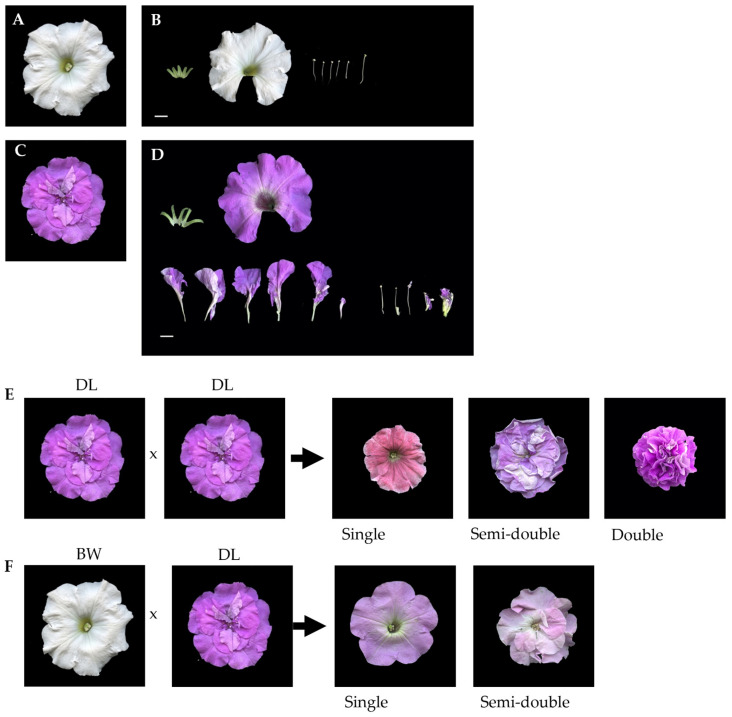
Floral characteristics and segregation patterns in Petunia. (**A**) Top view of ‘Baccarat White’ (BW, white). (**B**) Floral dissection of ‘Baccarat White’ (BW), showing individual floral components. (**C**) Top view of ‘Duo Lavender’ (DL, purple). (**D**) Floral dissection of ‘Duo Lavender’ (DL), illustrating its structural components. (**E**) Segregation pattern of self-crossed progeny from ‘Duo Lavender’ (DL). The offspring exhibited three distinct floral forms: single, semi-double, and double flowers. (**F**) Segregation pattern of progeny derived from a cross between and ‘Duo Lavender’ (DL) and ‘Baccarat White’ (BW). The hybrid progeny segregated into single and semi-double flower types. Scale bars = 1 cm.

To see the genetic characteristics of the double flower trait, crossing experiments were conducted between the single-flowered cultivar BW (Figure 1A,B) and the double-flowered cultivar DL (Figure 1C,D). Self-pollination of BW consistently yielded only single-flowered progeny, confirming its homozygous genotype in terms of the double-flowered trait. Self-pollination of DL was limited due to abnormal pistil development, leading to reduced seed production. In spite of the limited number of successfully obtained seeds, the selfed progeny showed phenotypic segregation of 4 (fully double):8 (semi-double):5 (single), which is not significantly different from 1:2:1 ratio (χ^2^ = 0.916, Figure 1E,F, Table 1), the expected segregation of Mendelian inheritance of a single and semi-dominant gene. Crossing between DL and BW yielded 196 progenies, comprising 94 single- and 102 semi-double-flowered plants, fitting a 1:1 segregation ratio (χ^2^ = 0.567, Table 1). An additional independent cross between DL and BW produced 142 progenies, consisting of 75 single- and 67 semi-double-flowered individuals, conforming to a ratio of 1:1 (χ^2^ = 0.502, Table 1). These results indicate that the double-flower trait in *Petunia* × *hybrida* is governed by a single gene with a semi-dominant inheritance pattern, where heterozygous individuals exhibit a semi-double phenotype, while homozygous recessive individuals remain single-flowered.

### 2.2. Morphology of Floral Organs and Their Surface Cell

**Figure 2:**
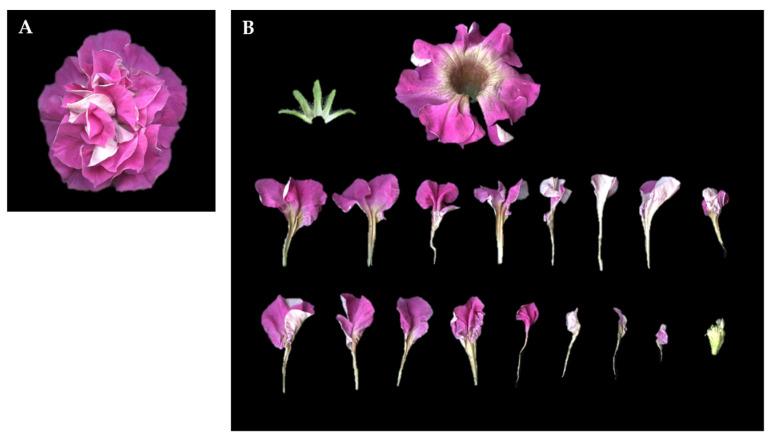
Floral characteristics and segregation patterns in petunia. (**A**) Top view of fully double-flower petunia. (**B**) Floral dissection of fully double-flower in F1 population, showing individual floral components.

Floral organ structures were compared among single-, semi-double-, and double-flowered petunias, with a focus on differences in petal number, stamen development, and floral organ morphology. In the single-flowered cultivar BW, floral organs followed a typical whorled arrangement, developing sequentially from the outermost layer as sepals, petals, stamens, and pistils (Figure 1A). In contrast, the semi-double-flowered cultivar DL exhibited an increased number of petals, with additional petaloid structures present between the outermost corolla and stamens. Some stamens in DL displayed intermediate petalization, with expanded filament bases and partially fused petal-like tissue at their tips (Figure 1D). In the progeny resulting from DL × DL crosses, a subset of fully double-flowered plants showed a significantly higher degree of petal multiplication than DL, forming a compact floral structure with layered petal whorls (Figure 2A). Floral dissections revealed that in these fully double-flowered plants, stamens were absent, and only sepals, petals, and morphologically altered pistils remained (Figure 2B).

**Figure 3:**
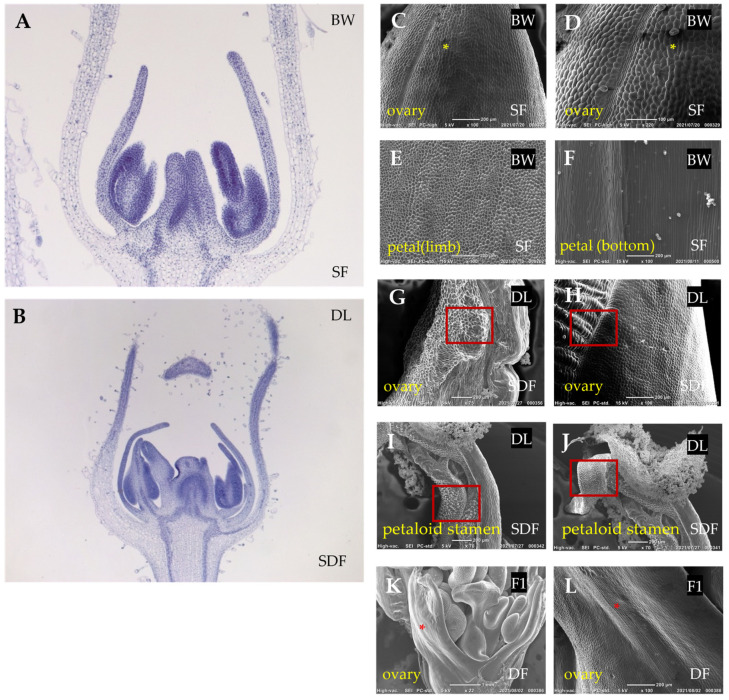
Floral and epidermal characteristics of single, semi-double, and double flowers in *Petunia* × *hybrida*. (**A**,**B**) Paraffin section images of floral structures in single-flower ‘Baccarat White’ (BW) and semi-double-flower ‘Duo Lavender’ (DL). (**C**–**L**) Scanning electron microscopy (SEM) images of floral organ epidermal tissues across different flower types. Floral organ types are labeled in yellow at the lower left of each panel, while flower types are indicated in the lower right. The cultivar name for each sample is indicated in the upper right corner of the respective figure. (**C**–**F**) SEM images of floral organs in BW (SF), including the ovary (**C**,**D**) and petal structures (**E**,**F**). Ovary epidermal cells (**C**,**D**) display regular polygonal shapes with visible stomata. Petal epidermal cells exhibit typical conical projections in the limb region (**E**) and elongated cells at the petal base (**F**). Yellow asterisks (*) indicate corresponding anatomical regions used for cross-panel comparison. (**G**,**H**) SEM images of the ovary in DL (SDF). The red rectangles mark epidermal regions with petal-like features: conical cells in (**G**), similar to the petal limb, and elongated cells in (**H**), resembling the petal base (**I**,**J**). SEM images of petaloid stamens in DL (SDF). (**K**,**L**) SEM images of the ovary in fully double-flowered individuals (DF) from the F_1_ population. Red asterisks (*) indicate corresponding regions across panels. The ovary epidermis shows a similar morphology to that of single-flowered plants. Additional internal floral structures, including stamens and pistil-like organs, are observed.

Paraffin sections were prepared to examine the whorl structure, confirming that BW flowers consist of a single petal whorl, while DL flowers exhibit at least two distinct layers of petal whorls (Figure 3A,B). In addition, pistils in DL showed abnormal structure, including an increased ovary size and irregular carpel morphology, differing from the typical pistil structures observed in BW.

Abnormal structure of DL floral organs were also observed in the organ surface structure observed by scanning electron microscopy (SEM) observation (Figure 3C–L). In BW, ovary epidermal cells exhibited an irregular polygonal shape with clearly visible stomata (Figure 3C,D). Limb epidermal cells displayed uniform conical projections characteristic of petal surface texture (Figure 3E). Basal corolla cells were elongated (Figure 3F), similar to those typically observed in normal petal development.

In semi-double-flowered petunias DL, the stamen filaments and anthers exhibited surface features resembling those of petal epidermal cells (Figure 3I,J). The filaments showed elongated epidermal cells with ridged surfaces, while anthers displayed partial conical projections, differing from the smooth epidermis of typical stamens. Additionally, epidermal cells in the pistils exhibited a shift in morphology, with some regions displaying conical structures similar to petal epidermal cells (Figure 3G,H).

In fully double-flowered petunias, abnormalities in pistil development were even more pronounced. Although SEM analysis showed that pistil epidermal cells retained a morphology similar to that of normal single-flower petunias (Figure 3K,L), the overall floral structure was extensively altered, with additional stamen-like and pistil-like tissues appearing within the carpel. These structures formed layered floral components, resembling a secondary floral arrangement embedded within the primary flower (Figure 3K). These observations suggest that the homeotic transformation of stamens and pistils into petaloid structures is associated with genetic alterations affecting floral organ identity specification in double-flowered petunias.

### 2.3. Sequence Variation of Double-Flower-Related Genes in Petunia × hybrida

The whole-genome sequencing of ‘Baccarat White’ (BW) and ‘Duo Lavender’ (DL) generated a 1.5 GB scaffold database. To identify genetic variants associated with floral organ differentiation, sequences of genes related to the ABC model were examined. A total of four B-class genes (*PhGLO1*, *PhGLO2*, *PhDEF* (*DEFICIENS*), and *PhTM6* (*TOMATO MADS BOX GENE6*)) and two C-class genes (*pMADS3* (*PETUNIA MADS BOX GENE3*) and *FBP6* (*FLORAL BINDING PROTEIN6*)) were identified in the assembled genome.

To further explore genetic factors involved in floral organ identity, full-length sequences of *AP2* family genes were retrieved from publicly available genomic data of *Arabidopsis thaliana* (TAIR10) [36,37], *Petunia axillaris* (v1.6.2) [2] and *Petunia inflata* (v1.0.1) [2]. A comparison of these sequences led to the identification of eight *euAP2* genes in *Petunia axillaris*: *PeaxiROB1*, *PeaxiROB2*, *PeaxiROB3*, *PeaxiBEN*, *PeaxiBOB*, and three additional unnamed *AP2* family members (*Peaxi162Scf01024g00326.1*, *Peaxi162Scf00072g00229.1*, and *Peaxi162Scf00389g00028.1*).

Similarly, nine *euAP2* genes were identified in *Petunia inflata*, including *PeinfROB1*, *PeinfROB2*, *PeinfROB3*, *PeinfBEN*, *PeinfBOB*, and four additional *AP2* genes (*Peinf101Scf12294g00004.1*, *Peinf101Scf01705g00017.1*, *Peinf101Scf01310g01017.1*, and *Peinf101Scf01034g02005.1*). Homologous sequences of these *AP2* family genes were also identified in the BW and DL scaffold databases, confirming their presence in both cultivars (Tables S1 and S4).

**Figure 4:**
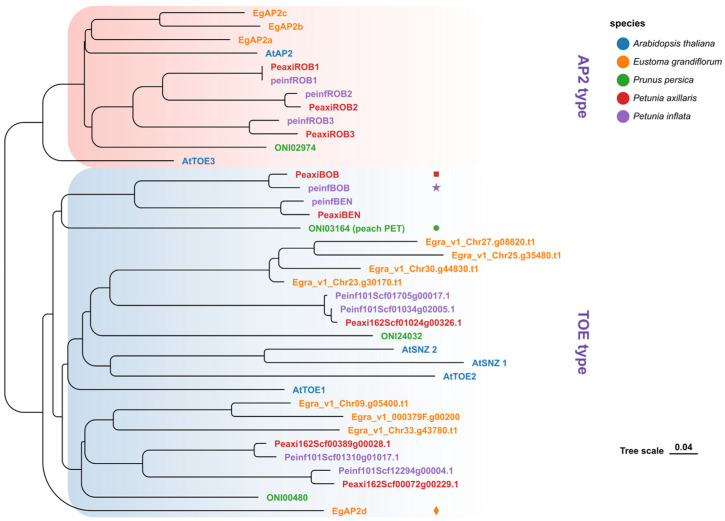
Molecular characterization and evolutionary analysis of the petunia AP2 family. Phylogenetic relationships of *AP2* family gene (AP2- and TOE-type) members among *Arabidopsis thaliana* and other species. The evolutionary tree includes sequences from *Arabidopsis thaliana* (*At*), *Eustoma grandiflorum* (*Egra*), *Prunus persica* (*ONI*), *Petunia axillaris* (*Peaxi*), and *Petunia inflata* (*Peinf*). The TOE-type and AP2-type clades are highlighted in red and blue, respectively. Different leaf colors indicate species, as shown in the legend on the right. Distinct symbols (square, star, circle, and diamond) represent genes associated with the double-flower phenotype in different species.

To investigate the phylogenetic relationships among these genes, their sequences were compared, and they were categorized into two major clades in *Petunia axillaris*: the AP2-type (*PeaxiROB1*, *PeaxiROB2*, *PeaxiROB3*) and the TOE-type (*PeaxiBEN*, *PeaxiBOB*, and three unnamed genes) (Figure 4). Notably, *PeaxiBEN* and *PeaxiBOB* exhibited high sequence similarity to *ONI03164* from *Prunus persica* (peach), a gene previously reported to be associated with the double-flower trait. This finding suggests that *AP2* genes may play an essential role in floral organ development in petunia (Figure 4).

**Figure 5:**
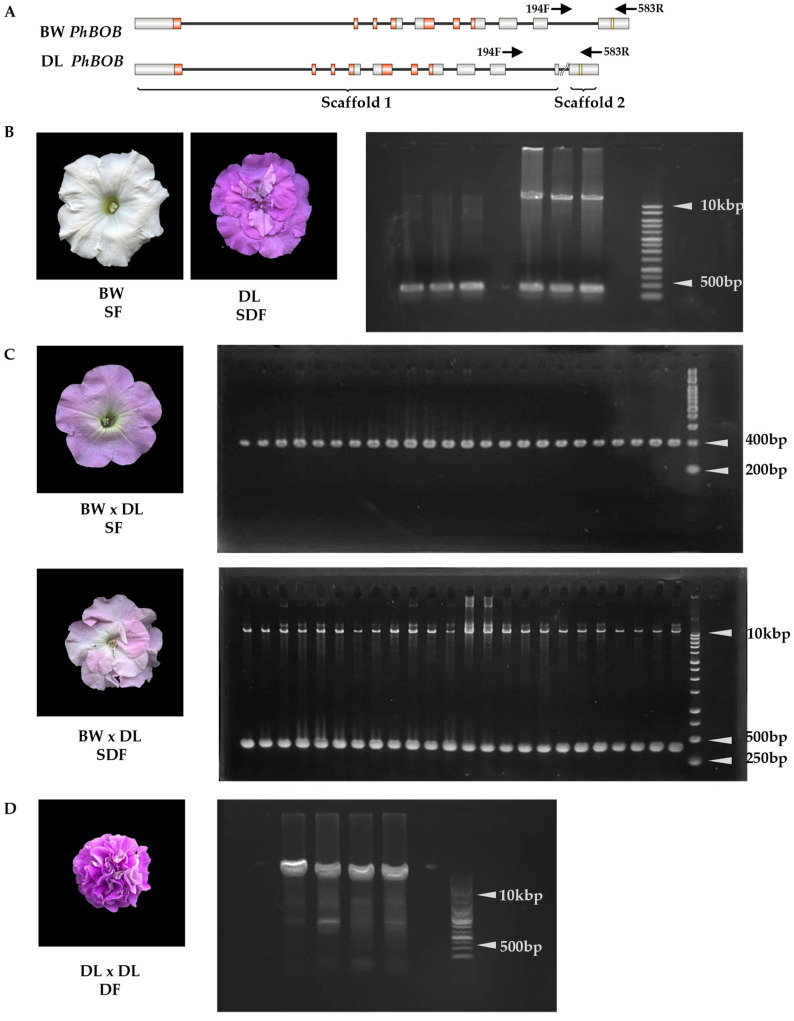
Linkage analysis of *PhBOB* and its association with the double-flower trait in *Petunia* × *hybrida.* (**A**) Gene structure of the TOE-type gene *PhBOB* in ‘Baccarat White’ (BW) and ‘Duo Lavender’ (DL). Solid boxes represent coding regions (CDS), with red boxes indicating the AP2 domain. The yellow box marks the *miR172* binding site. A putative ~10 kb insertion is present in the DL allele, positioned upstream of the *miR172* binding site. This insertion disrupts the continuity of the gene, resulting in two scaffolds in the DL genomic assembly. The locations of the primers 194F and 583R used for PCR genotyping are indicated by arrows. The diagram was generated using IBS 2.0 [38]. (**B**) PCR amplification of *PhBOB* in parental lines. The left three lanes represent three biological replicates of ‘Baccarat White’ (BW; single flower), each derived from independent seeds, showing a single 390 bp fragment. The right three lanes represent three independent individuals of ‘Duo Lavender’ (DL; semi-double flower), each showing both a 390 bp fragment and an additional ~10 kb fragment, indicating the presence of the insertion. (**C**) Genotyping results of F_1_ progeny derived from BW × DL. Single-flower (SF) individuals show only the 390 bp band, while semi-double-flower (SDF) individuals display both bands, consistent with heterozygosity for the insertion. (**D**) Genotyping of self-pollinated DL progeny. Individuals with fully double flowers (DF) exhibit homozygosity for the insertion (~10 kb). White arrowheads indicate the band size.

A comparison of gene sequences between BW and DL revealed no differences in *PhGLO1* and *PhGLO2*, *PhDEF*, and *PhTM6* (Tables S2 and S5). Similarly, no notable sequence variations were detected in C-class genes, including *pMADS3* and *FBP6* (Tables S3 and S6). However, a structural variation was detected in *PhBOB* between BW and DL, particularly in the upstream region of the *miR172* binding site. In BW, *PhBOB* exhibited an intact structure, whereas in DL, the gene was fragmented into two non-contiguous sequences. One fragment retained two EAR motifs, two AP2 domains, a linker region, and an NLS domain, while the other contained the region downstream of the *miR172* binding site. No repetitive sequences were found between these two fragments, indicating that the structural variation in DL was caused by a large-scale insertion upstream of the *miR172* binding site (Figure 5A and Figure S1).

Additionally, large deletions were identified in *PhROB1* and *PhROB3* in both BW and DL. However, these variations were restricted to intronic regions, suggesting that they may have a limited impact on gene expression (Tables S1 and S4).

The presence of this insertion in *PhBOB* indicates that structural modifications in *AP2* family genes may contribute to floral organ identity changes. These findings are consistent with those of Gattolin et al. (2020) [17], further supporting the role of *PhBOB* in regulating petal number and the double-flower trait in petunia.

### 2.4. The 10 kbp Insertion in *PhBOB* Is Highly Linked to Double-Flowering in Petunias

Scaffold sequences upstream and downstream of *PhBOB* were compared between BW and DL. A forward primer was designed in the conserved upstream region and a reverse primer in the conserved downstream region (Figure 5A). These primers were used to genotype both the parental lines and the progeny derived from the BW × DL cross, as well as the selfed population of DL.

In BW, PCR amplification produced a single 390 bp fragment. In contrast, DL individuals showed two distinct bands: a 390 bp fragment and an additional ~10 kb fragment, which corresponds to a large insertion at the target site and reflects a heterozygous genotype (Figure 5B).

In the BW × DL F_1_ population, single-flowered plants exhibited only the 390 bp fragment, whereas semi-double-flowered plants displayed both the 390 bp and ~10 kb bands. PCR analysis of 96 single-flowered and 96 semi-double-flowered individuals confirmed this pattern (Figure 5C). All single-flowered plants were homozygous for the absence of the insertion (−/−), and all semi-double-flowered plants were heterozygous (+/−). No individuals homozygous for the insertion (+/+) were detected in the F_1_ population (Table 2).

To further clarify the relationship between the insertion and the floral phenotype, genotyping was performed on selfed progeny of DL. In this population, fully double-flowered individuals (DF) exhibited only the ~10 kb band, consistent with homozygosity for the insertion (+/+) (Figure 5D).

These results indicate that the 10 kbp insertion upstream of the *miR172* target site in *PhBOB* is tightly linked to the double-flower trait and is likely involved in petal proliferation and floral organ transformation.

### 2.5. Expression of MADS-Box and AP2 Genes in BW and DL

**Figure 6:**
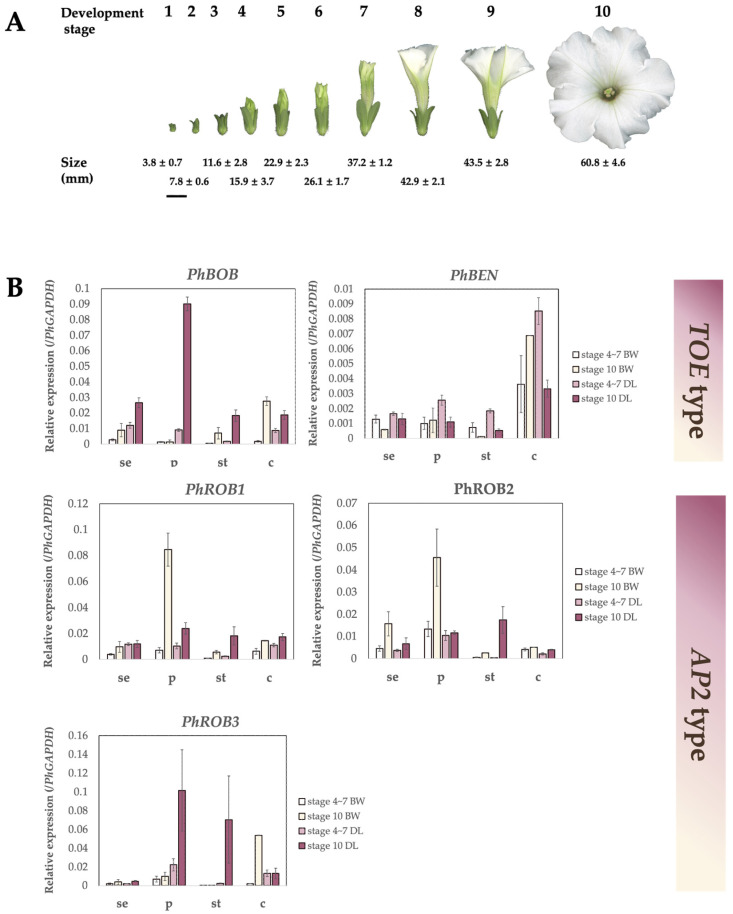
Expression of A-class genes in different floral organs. (**A**) Developmental stages of petunia flowers. Representative images of flower buds at 10 developmental stages from ‘Baccarat White’. The corresponding bud lengths for each stage were as follows: Stage 1: 3.8 ± 0.7 mm, Stage 2: 7.8 ± 0.6 mm, Stage 3: 11.6 ± 2.8 mm, Stage 4: 15.9 ± 3.7 mm, Stage 5: 22.9 ± 2.3 mm, Stage 6: 26.1 ± 1.7 mm, Stage 7: 37.2 ± 1.2 mm, Stage 8: 42.9 ± 2.1 mm, Stage 9: 43.5 ± 2.8 mm, and Stage 10: fully opened flowers. Scale bar = 10 mm. (**B**) Quantitative real-time PCR (qRT-PCR) analysis of A-class genes. Expression levels of *PhBOB*, *PhBEN*, *PhROB1*, *PhROB2*, and *PhROB3* were analyzed in sepals (se), petals (p), stamens (st), and carpels (c) at early (stages 4–7) and late (stage 10) developmental stages in ‘Baccarat White’ (BW) and ‘Duo Lavender’ (DL). Gene expression is normalized to *PhGAPDH*. The results show distinct transcriptional patterns, suggesting their role in floral organ identity and differentiation. Error bars represent the standard error of the mean (n = 4).

Expression patterns of A-class (*AP2* family genes) and C-class (*pMADS3* and *FBP6*) genes were examined in different floral organs of BW and DL using qRT-PCR. Gene expression was quantified at ten developmental stages of flower buds (Figure 6A), with early floral development (stages 4–7) and late floral development (stage 10) selected as key time points for comparison.

Among A-class genes, *BEN* expression was consistently low (<0.01) across all floral organs, with no significant differences between BW and DL (Figure 6B). In contrast, *BOB* expression was higher in petal tissues. In DL, *BOB* expression was 6.8-fold higher than in BW during stages 4–7 and increased to 68.7-fold at stage 10. *ROB3* showed a similar pattern, with a 3.1-fold increase in DL petals during early development and a 10.3-fold increase at stage 10.

*ROB1* and *ROB2* exhibited moderate expression in petals, particularly in stage 10 BW flowers, where transcript levels reached 0.1 and 0.05, respectively. Their expression in sepals, stamens, and pistils remained low with minimal variation.

**Figure 7:**
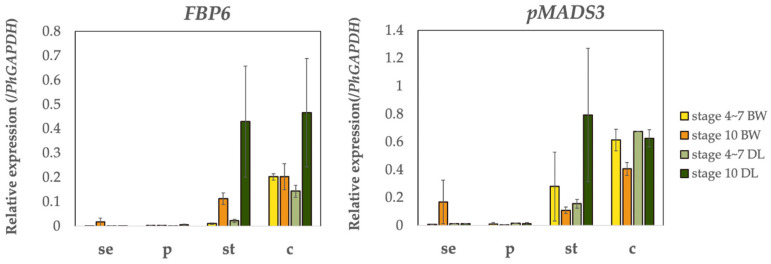
Expression of C-class genes in different floral organs. Quantitative real-time PCR (qRT-PCR) analysis of C-class genes. Expression levels of *FBP6* and *pMADS3* were assessed in sepals (se), petals (p), stamens (st), and carpels (c) at early (stages 4–7) and late (stage 10) developmental stages in ‘Baccarat White’ (BW) and ‘Duo Lavender’ (DL). Gene expression is normalized to *PhGAPDH*. The results indicate their functional involvement in reproductive organ development, with notable expression in stamens and carpels. Error bars represent the standard error of the mean (n = 4).

C-class genes (*pMADS3* and *FBP6*) were primarily expressed in stamens and pistils, with minimal levels detected in sepals and petals (Figure 7). *FBP6* expression in stamens increased significantly at stage 10, with a 19.4-fold upregulation compared to stages 4–7. This increase was more pronounced in DL, where *FBP6* transcript levels were 3.8-fold higher than in BW at stage 10. Similarly, *FBP6* expression in DL pistils was 2.3-fold higher than in BW at stage 10. *pMADS3* also showed significantly higher expression in DL stamens, with transcript levels 3.8-fold higher than in BW.

## 3. Discussion

### 3.1. PhBOB 10 kb Insertion Is Responsible for the Double-Flower Trait in Petunia

The double-flower trait in *Petunia* × *hybrida* shows a strong association with a 10 kb insertion located upstream of the *miR172* target site in *PhBOB*, an *AP2*-like gene, suggesting a potential disruption of *miR172*-mediated post-transcriptional regulation. *miR172* is known to play a crucial role in floral development by targeting *AP2* genes, regulating their expression across diverse plant species. In *Arabidopsis thaliana*, *miR172* directly suppresses *APETALA2* (*AP2*), influencing floral organ identity [39], while in *Brassica napus*, a genome-wide analysis confirmed the regulatory interaction between *miR172* and *AP2* genes [40]. Similarly, in apple, the overexpression of *miR172* significantly alters flowering time and floral organ identity, reinforcing its conserved function in floral patterning [41]. In tomato, *miR172* has also been reported to regulate fruit development through its control of *AP2* genes [42].

In ornamental and fruit-bearing species such as peach (*P. persica*), rose (*R. chinensis*), and Japanese apricot (*P. mume*), mutations in the *miR172* binding site of *AP2*-like genes have been shown to prevent their degradation, resulting in elevated transcript levels and increased petal number [15,28,43,44]. These cases support the broader relevance of *miR172*-*AP2* regulatory interactions in floral morphology.

In addition to directly regulating *AP2* genes, *miR172* is also involved in defining the spatial expression boundaries of B-class genes, which specify petal and stamen identities. In *Arabidopsis*, *miR172* establishes the boundary for *AP3* and *PI* expression in floral meristems, ensuring proper organ differentiation [45]. This spatial control is essential for preventing homeotic transformations between floral organs, highlighting the broader regulatory role of *miR172* in flower development.

In petunia, the insertion identified near the *miR172* binding site of *PhBOB* may interfere with this regulatory mechanism. Since *AP2*-like genes are known targets of *miR172*, the insertion could affect miRNA binding or stability, potentially resulting in altered gene expression. In support of this, PCR-based genotyping of F1 progenies revealed complete co-segregation between the 10 kb insertion and the double-flower phenotype. Single-flower individuals lacked the insertion, whereas all semi-double individuals were heterozygous, carrying both the wild-type and inserted alleles.

To assess the impact of this structural variation, gene expression analysis was performed. qRT-PCR results showed a substantial increase in *PhBOB* expression in petal tissues of double-flowered individuals, reaching levels approximately 69 times higher than those in single-flowered plants. This strong correlation between the presence of the insertion and elevated *PhBOB* transcript levels suggests a potential regulatory effect of the insertion on gene expression. As the overexpression of *AP2*-like genes has been linked to increased petal numbers in other species, *PhBOB* is likely to contribute significantly to double-flower formation in petunia.

### 3.2. PhBOB 10 kb Insertion Is Highly Conserved in Solanaceae and Contains CMC-EnSpm Transposon Sequences

BLAST analysis of the upstream 670 bp region of the 10 kb insertion in *PhBOB* (Table S9) revealed 98 significant matches across *Solanoideae* species, showing a high level of sequence similarity within this subfamily. In *Capsicum* species, only two matches were identified, reflecting a marked reduction in similarity compared to other *Solanoideae* members. This divergence highlights that while the upstream region is conserved in many *Solanoideae* species, it may have been lost or modified in certain lineages, reflecting different evolutionary pressures acting on this genomic region.

The downstream 652 bp region of the insertion (Table S9) was also found to be conserved across multiple *Solanaceae* species, including *Solanum demissum*, *Solanum lycopersicum* (tomato), *Solanum tuberosum* (potato), *Lycium barbarum* (goji berry), *Nicotiana tomentosiformis*, *Nicotiana sylvestris* (wood tobacco), and *Capsicum annuum* (chili pepper). This cross-genus presence within the Solanaceae family shows that the region is more broadly conserved. While its exact function remains unknown, its consistent presence across divergent species implies that it may have some regulatory significance, though experimental validation is still required.

Sequence analysis of the downstream region detected three segments with homology to CMC-EnSpm transposons, with sequence identities of 67% (6 × 10^−39^), 69% (3 × 10^−29^), and 78% (4 × 10^−28^), respectively. CMC-EnSpm transposons are one of the most abundant DNA transposon families in plant genomes and are known to contribute to genome evolution and structural variation. Ishimori et al. (2015) [14] reported that CMC-EnSpm elements are a major component of the *Eustoma grandiflorum* (lisianthus) genome, where RepeatMasker analysis showed that DNA transposons account for 92.0 Mb (12.8%) of the genome, with the CMC-EnSpm subfamily comprising 11.3 Mb, making it the most dominant DNA transposon subfamily. The widespread distribution of CMC-EnSpm elements in plant genomes supports their role as major contributors to genome structure and functional regulation.

Beyond their structural role, CMC-EnSpm transposons are known to influence gene expression through chromatin modifications and transcriptional regulation. Studies have shown that these transposons can modify chromatin structure, alter DNA methylation patterns, and provide novel promoter or enhancer sequences that regulate nearby genes [46,47]. In *Populus trichocarpa*, CMC-EnSpm transposons were found to regulate gene expression by modifying DNA methylation, leading to changes in gene activity in response to environmental stimuli [47]. Similarly, in *Dioscorea alata*, transcriptionally active CMC-EnSpm elements were associated with specific gene expression patterns, suggesting that they play a regulatory role in gene function. These studies highlight the functional significance of CMC-EnSpm transposons in modulating gene expression across plant species [48].

Given the strong evidence supporting the regulatory role of CMC-EnSpm transposons, it is likely that the 10 kb insertion in *PhBOB* plays a role in its overexpression in double-flower petunias. The presence of transposon sequences in the insertion suggests that chromatin modifications or alterations in transcriptional regulation may contribute to the upregulation of PhBOB in petal tissues. Further studies are needed to confirm whether the CMC-EnSpm insertion directly influences *PhBOB* expression through changes in DNA methylation, chromatin accessibility, or enhancer activity. Understanding the precise mechanism of how this transposon insertion affects PhBOB regulation will provide valuable insights into the genetic basis of the double-flower trait in petunia and could inform strategies for breeding novel floral morphologies in ornamental plants.

### 3.3. The Expression of C-Class Genes (FBP6 and pMADS3) Is Controlled by PhBOB

C-class genes play a critical role in defining reproductive organ identity in flowering plants, regulating the formation of stamens and carpels. While some species retain both *PLE* and *AG* homologs, others possess only one functional C-class gene [49,50]. In petunia, only two genes, *pMADS3* and *FBP6*, function as C-class genes, both of which are homologs of *AGAMOUS* (*AG*), the key floral homeotic gene responsible for specifying stamens and carpels.

In single-flower petunias, *FBP6* and *pMADS3* are highly expressed in stamens and carpels, consistent with their role in reproductive organ identity. However, in semi-double-flower individuals, these genes remain highly expressed in stamens and carpels but are notably suppressed in additional petal regions. This spatial shift in gene expression indicates that *PhBOB* does not completely repress C-class genes but instead modifies their spatial expression domains, which is associated with the transformation of stamens into petal-like structures. This change in gene expression pattern may explain the floral phenotype of semi-double individuals, where extra petals develop while the reproductive organs are still partially retained.

A similar regulatory mechanism has been observed in other double-flower species, where *AP2*-like genes modulate, rather than completely repress, the spatial expression of C-class genes. In rose, peach, and lisianthus, mutations in *AP2* genes have been shown to disrupt the spatial expression of C-class genes, leading to the petalization of reproductive organs while allowing some stamen and carpel development to persist [15,28,43,44]. This pattern is consistent with the role of *PhBOB* in petunia, which appears to act by altering the spatial domain of *FBP6* and *pMADS3* rather than fully silencing them. The ability of *PhBOB* to modulate C-class gene expression may be a key factor in the formation of semi-double flowers, where intermediate stamen–petal transformation occurs rather than a complete conversion of reproductive organs into petal tissues.

### 3.4. AP2 Genes in the TOE-Type Clade Are Associated with the Double-Flower Trait

The double-flower phenotype in *Petunia* × *hybrida* is strongly associated with *PhBOB*, a TOE-type *AP2* gene. Phylogenetic analysis shows that *PhBOB* belongs to a clade known as *PETALOSA*, which includes genes such as *Prupe.6G242400* in peach, *Dca21030.1* in carnation, and *XP_024186592* in rose [15,17]. In each case, the gene is expressed in floral organs, and its overexpression correlates with changes in floral identity.

In petunia, *PhBOB* shares not only phylogenetic similarity with these *PETALOSA* genes but also functional characteristics. The insertion found upstream of *PhBOB* is associated with its elevated expression in petal tissues, and semi-double flowers show altered spatial expression of floral identity genes consistent with AP2-related regulatory changes. These parallels in sequence, expression, and phenotype reinforce the classification of *PhBOB* as a functional member of the *PETALOSA* group.

While the *PETALOSA* lineage is clearly associated with dominant double-flower mutations, other TOE-type *AP2* genes outside this clade have also been implicated in the regulation of petal proliferation. In *Eustoma grandiflorum*, the *EgAP2d* gene is strongly linked to the double-flower trait but is phylogenetically distant from the *PETALOSA* clade, suggesting independent evolutionary origins for this regulatory function [14,51]. Similarly, in *Prunus mume* (Japanese apricot), *EgAP2L* contributes to the double-flower phenotype but does not belong to the *PETALOSA* lineage [18], further supporting the hypothesis that multiple TOE-type *AP2* genes, beyond those classified as *PETALOSA*, can regulate floral organ identity. These findings suggest that the ability of TOE-type *AP2* genes to modulate double-flower formation has evolved independently in different plant lineages, indicating a broader functional role for this gene family in floral development.

Beyond the TOE-type group, *AP2*-type genes such as *PhROB3* have also been identified in petunia. In semi-double flowers, *PhROB3* exhibits differential expression between petals and stamens, and its involvement has been proposed in floral boundary regulation. Although phylogenetically distinct from TOE-type genes, its floral expression pattern and response to morphological changes suggest that *PhROB3* may interact with *PhBOB* or respond to similar regulatory cues. This observation is consistent with findings in other species where different AP2 subfamilies co-regulate floral patterning.

Taken together, these findings demonstrate that while *PETALOSA* genes represent a distinct lineage of TOE-type *AP2* genes responsible for dominant double-flower mutations, other TOE-type and AP2-type genes may also contribute to petal number regulation. The interaction between these gene families underlies the diversity of floral forms observed in ornamental plants, providing valuable insights into the molecular mechanisms driving double-flower evolution.

## 4. Materials and Methods

### 4.1. Plant Materials and Growth Conditions

This study utilized two commercial cultivars of *Petunia* × *hybrida*: ‘Baccarat White’ (BW, single-flower, white) and ‘Duo Lavender’ (DL, semi-double flower, purple). Plants were cultivated under controlled environmental conditions, with a 16 h photoperiod using white LED lights at a photosynthetic photon flux density (PPFD) of 200 μmol m^−2^s^−1^. Temperature conditions were maintained at 23 °C during the day and 27 °C at night to support optimal growth and flowering.

For crossing experiments, all stamens were removed from BW flowers before anthesis to prevent self-pollination. Pollen from DL flowers was manually transferred onto the stigma of emasculated BW flowers, and the pollinated flowers were immediately bagged to prevent cross-contamination from other pollen sources. Pollination was conducted in May 2019 and January 2020 to ensure seasonal reproducibility of the results.

To facilitate self-pollination in DL, high-temperature treatment was applied to induce pollen production. Each flower was individually bagged to ensure self-pollination without external pollen interference, allowing for controlled genetic analysis of the semi-double flower trait.

### 4.2. Paraffin Sectioning

For histological analysis, flower buds of Baccarat White (BW, single-flower) and Duo Lavender (DL, semi-double flower) petunias (*Petunia* × *hybrida*) were collected at different developmental stages and immediately fixed in FAA solution (5% formaldehyde, 5% acetic acid, 90% ethanol, *v*/*v*) at 4 °C overnight to preserve tissue integrity.

After fixation, samples were dehydrated through a graded ethanol series (50%, 70%, 85%, 90%, and 100%) and subsequently cleared using Histoclear to enhance wax infiltration. Following appropriate pre-processing, samples were embedded in paraffin to facilitate sectioning. A rotary microtome (Leica RM2235, Leica Biosystems, Nussloch, Germany) was used to obtain continuous sections (8 μm thickness), which were then mounted onto poly-L-lysine-coated slides to improve tissue adhesion and prevent detachment during staining and observation.

To remove paraffin residues, sections were deparaffinized using Histoclear, followed by rehydration through a descending ethanol gradient (100%, 95%, 90%, 80%, 70%, and 50%) to restore tissue hydration. Sections were then sealed using Entellan New mounting medium (Merck) for long-term preservation and microscopic examination.

For tissue staining, hematoxylin staining was applied to visualize floral organ structures, including sepals, petals, stamens, and carpels, to examine cell morphology and tissue differentiation. Finally, microscopic observation and imaging were conducted using a light microscope (Leica DM500, Leica Microsystems, Germany) to document tissue architecture and analyze morphological changes during different floral developmental stages.

### 4.3. Scanning Electron Microscopy (SEM) Observation

To examine the cellular morphology of leaf and floral organ surfaces in *Petunia* × *hybrida*, scanning electron microscopy (SEM) was conducted following standard sample preparation procedures to maintain tissue integrity and optimize imaging resolution.

Freshly collected leaves and floral organs were immersed in NanoSuit Solution I to form a protective layer, reducing electron beam-induced damage and enhancing sample conductivity. Excess solution was carefully blotted using filter paper or Kimwipes to remove residual liquid that could interfere with imaging.

Samples were then mounted onto SEM stubs using conductive double-sided adhesive tape to ensure secure adhesion and minimize movement or vibration during scanning. The specimens were subsequently transferred to a JCM-6000 scanning electron microscope (JEOL, Japan) for imaging. The accelerating voltage and working distance were adjusted to obtain clear microstructural images of the epidermal cells on petunia leaves and floral organs.

### 4.4. Whole-Genome Sequencing

Whole-genome sequencing (WGS) was performed on *Petunia* × *hybrida* cultivars ‘Baccarat White’ (BW, single-flower) and ‘Duo Lavender’ (DL, semi-double flower) using Next-Generation Sequencing (NGS) technology to obtain genome-wide sequence information and provide a foundation for subsequent gene identification and variant analysis.

Genomic DNA was extracted from fresh leaf tissues, and sequencing libraries were constructed using a random fragmentation approach with an insert size of 500 bp. High-throughput sequencing was carried out on the Illumina HiSeq X platform using 150 bp paired-end sequencing to ensure high coverage and sequencing depth. A total of 66 GB of raw sequencing data was generated for DL, and 75 GB for BW.

The raw sequencing data underwent quality control (QC) filtering, where low-quality reads, adapter sequences, and PCR duplicates were removed to ensure high-accuracy genome assembly. The filtered high-quality reads were then de novo assembled using ABySS [52], resulting in a 1.5 GB scaffold database, corresponding to the genome sequences of both BW and DL cultivars.

### 4.5. Identification and Screening of AP2 Family Genes in Petunia and Other Ornamental Plants

To comprehensively identify AP2 family genes in *Petunia* × *hybrida* and other ornamental plants, two strategies were employed to obtain high-quality AP2 candidate sequences. An annotation-based approach was first applied by searching for “AP2” in the Plant Transcription Factor Database (PlantTFDB, https://planttfdb.gao-lab.org/, accessed on 22 April 2025), selecting the target species to retrieve annotated AP2 gene sequences. This method leveraged an existing transcription factor database to facilitate the rapid identification of AP2 gene candidates.

To further refine the dataset, a Hidden Markov Model (HMM)-based screening approach was used. Genomic sequences, protein FASTA files, and GFF3 annotation files for *Petunia* were downloaded from publicly available genome databases. The HMM model for the AP2 domain was retrieved from the InterPro Pfam database (https://www.ebi.ac.uk/interpro/entry/pfam/PF00847/logo/, accessed on 22 April 2025), and an initial search was conducted using hmmsearch with an E-value threshold of 1.2 × 10^−28^. To ensure the accuracy of the predicted AP2 domain sequences, further validation was performed using SMART (http://smart.embl-heidelberg.de/, accessed on 22 April 2025) and the NCBI Conserved Domain Database (CDD, http://www.ncbi.nlm.nih.gov/Structure/cdd/cdd.shtml, accessed on 22 April 2025). Verified AP2 domain sequences were aligned using ClustalW, and a petunia-specific HMM model was constructed with hmmbuild for a refined hmmsearch. A final selection threshold of E-value < 0.001 was applied to obtain high-confidence AP2 family genes. Given the presence of multiple transcript isoforms, GFF3 annotation files were utilized to match gene loci with corresponding mRNA sequences, and redundant sequences were removed to ensure the accuracy of the final AP2 gene dataset.

The genomic data used in this study were sourced from multiple databases: *Arabidopsis thaliana* data were obtained from Ensembl Plants (https://plants.ensembl.org/Arabidopsis_thaliana/Info/Annotation/, accessed on 08 December 2024), *Eustoma grandiflorum* from Plant Garden (https://plantgarden.jp/ja/download/t52518/t52518.G001/, accessed on 22 April 2025), *Prunus persica* from NCBI WGS Project AKXU02 (https://www.ncbi.nlm.nih.gov/datasets/genome/GCF_000346465.2/, accessed on 22 April 2025), while *Petunia axillaris* and *Petunia inflata* genomic data were accessed via the Sol Genomics Network (https://solgenomics.net/organism/Petunia_axillaris/genome, accessed on 8 December 2024) and (https://solgenomics.net/organism/Petunia_inflata/genome, accessed on 8 December 2024), respectively. The sequences used in phylogenetic analysis are listed in Supplementary Table S7. The phylogenetic tree was constructed using the Neighbor-Joining (NJ) method, with the Genetic Distance Model set to Jukes–Cantor. Tree visualization and annotation were performed using tvBOT [53].

### 4.6. Sequence Alignment and Homolog Identification of ABC-Class Genes in Petunia × hybrida

The scaffold database was imported into Geneious R10.2.3 for further analysis. The identified AP2 target gene sequences from *Petunia axillaris* were used as query sequences for BLAST searches against the scaffold databases of ‘Baccarat White’ (BW) and ‘Duo Lavender’ (DL). The sequences with the lowest E-value in the BLAST results were selected as candidate homologs. If the identified scaffold fragment did not contain the full-length sequence of the target gene, the scaffold was used as a new query sequence for an additional BLAST search in the BW and DL scaffold databases to extend the target sequence. To ensure the reliability of the sequence alignment results, the Dotplot function in Geneious was utilized to analyze the similarity between the query and candidate sequences, confirming high sequence homology.

### 4.7. Linkage Analysis

Genomic DNA was extracted from 200 mg of leaf tissue using the Maxwell RSC PureFood GMO and Authentication Kit (Catalog No. AS1600). Each PCR reaction was performed using 80 ng of genomic DNA as the template, with amplification carried out using KOD FX Neo (TOYOBO) and specific primer sets (Table S8). The PCR conditions were as follows: 94 °C for 2 min, followed by 30 cycles of 98 °C for 10 s, 60 °C for 30 s, and 68 °C for 7 min, with a final extension at 72 °C for 7 min before being held at 4 °C. PCR products were separated by electrophoresis on a 0.5% SeaKem GTG agarose gel (100 V, 30 min, Mupid-ex system) and visualized using a gel imaging system.

### 4.8. Gene Expression Analysis

Total RNA was extracted using the Maxwell RSC Plant RNA Kit, with tissue samples flash-frozen in liquid nitrogen prior to extraction. RNA reverse transcription was performed using the ReverTra Ace qPCR RT Master Mix with gDNA Remover Kit (TOYOBO). Quantitative real-time PCR (qRT-PCR) was conducted on a StepOne Real-Time PCR System (Applied Biosystems, Waltham, Massachusetts, USA) using KOD SYBR qPCR Mix in a 96-well plate with a final reaction volume of 10 μL, following the manufacturer’s instructions. The primers used in qRT-PCR are listed in Supplementary Table S8.

To analyze the dynamic changes in gene expression during flower development, the floral developmental process of ‘Baccarat White’ (BW, single-flower) and ‘Duo Lavender’ (DL, semi-double flower) was categorized into ten stages (Stage 1 to Stage 10) based on flower bud size. The corresponding bud lengths for each stage were as follows: Stage 1: 3.8 ± 0.7 mm, Stage 2: 7.8 ± 0.6 mm, Stage 3: 11.6 ± 2.8 mm, Stage 4: 15.9 ± 3.7 mm, Stage 5: 22.9 ± 2.3 mm, Stage 6: 26.1 ± 1.7 mm, Stage 7: 37.2 ± 1.2 mm, Stage 8: 42.9 ± 2.1 mm, Stage 9: 43.5 ± 2.8 mm, and Stage 10: fully opened flowers. To investigate the expression patterns of candidate genes at key developmental stages, floral tissues were collected from pre-flowering stages (Stages 4 to 7, bud length: 15.9 to 37.2 mm) and post-flowering Stage 10.

qRT-PCR was performed under the following thermal cycling conditions: Holding stage: 98 °C for 2 min; cycling stage: 50 cycles of 98 °C for 10 s, 60 °C for 10 s, and 68 °C for 30 s; melt curve stage: 95 °C for 15 s, 60 °C for 1 min, and 99 °C for 15 s. All experimental data were analyzed using Microsoft Excel, with three biological replicates and two technical replicates performed for each gene.

## 5. Conclusions

This study confirms that the double-flower trait in petunia follows a single-gene, semi-dominant inheritance pattern through genetic analysis. Morphological characterization using paraffin sectioning and scanning electron microscopy (SEM) revealed clear differences in floral organ structure among single-flowered (SF), semi-double-flowered (SDF), and fully double-flowered (DF) petunias. At the molecular level, we identified a 10 kb insertion in *PhBOB* that is strongly associated with the double-flower trait and exhibits complete co-segregation in F1 populations. Gene expression analysis further demonstrated that *PhBOB* is significantly upregulated in double-flowered petunias, reaching expression levels up to 69-fold higher than in single-flowered cultivars, suggesting that its overexpression is a key driver of petal proliferation.

In addition to *PhBOB*, another AP2 family gene, *PhROB3*, was also markedly upregulated in petals and stamens, particularly in semi-double and fully double flowers, where partial homeotic transformation of stamens into petal-like structures was observed. This suggests that *PhROB3* may work in conjunction with *PhBOB* to regulate petal and stamen differentiation, adding another layer of complexity to petunia floral organ development.

## Figures and Tables

**Table 1:** Segregation patterns of flower types in parental lines, F_1_ hybrids, and self-pollinated progenies of Petunia, with expected Mendelian ratios and chi-square analysis.

Cross	Total	Flower Type			Exp. Ratio	*X* ^2^
		Single	Semi-Double	Double		
BW	8	8	0	0	-	-
DL	8	0	8	0	-	-
BW × BW	8	8	0	0	1:0:0	-
DL × DL	17	5	8	4	1:2:1	0.916
BW × DL rep1	196	94	102	0	1:1:0	0.567
BW × DL rep2	142	75	67	0	1:1:0	0.502

**Table 2:** The number of the presence of insertion in *PhBOB* BW × DL hybrid offspring. Individuals were genotyped based on the presence or absence of the 10 kbp fragment insertion in *PhBOB* using PCR.

Flower Phenotype	Genotype (−/−) (No Insertion)	Genotype (+/−) Heterozygous for Insertion	Total (n)
Single	96	0	96
Semi-double	0	96	96

## Data Availability

All data supporting the findings of this study are available within the paper and within its Supplementary Materials published online.

## Supplementary Materials

The following supporting information can be downloaded at: https://www.mdpi.com/article/10.3390/plants14091314/s1, Table S1. Nucleotide sequence of A-class genes from ‘Baccarat White’. Table S2. Nucleotide sequence of B-class genes from ‘Baccarat White’. Table S3. Nucleotide sequence of C-class genes from ‘Baccarat White’. Table S4. Nucleotide sequence of A-class genes from ‘Duo Lavender’. Table S5. Nucleotide sequence of B-class genes from ‘Duo Lavender’. Table S6. Nucleotide sequence of C-class genes from ‘Duo Lavender’. Table S7. Nucleotide sequence of phylogenetic analysis. Table S8. Oligo sequences used in this study. Table S9. Nucleotide sequence of the 10 kb insertion in *PhBOB*. Figure S1. Sequence alignment and schematic illustration of the putative insertion upstream of the *miR172* binding site in *PhBOB*.
