# Supplementary material for: An AP2-Family Gene Correlates with the Double-Flower Trait in Petunia × hybrida
Source: Plants (Basel). 2025 Apr 26;14(9):1314. doi: 10.3390/plants14091314 (PMC12073423; doi:10.3390/plants14091314)
Supplement: Supplementary file 1 [file plants-14-01314-s001.zip › plants-3575391-supplementary.pdf]

Article

An *AP2*-family Gene Correlates with the Double-Flower Trait in *Petunia x hybrida*

Tong Xie [1], and Saneyuki Kawabata [2,]

Graduate School of Agricultural and Life Sciences, The University of Tokyo; xielilixt@gmail.com
*Graduate School of Agricultural and Life Sciences, The University of Tokyo; skawabata@g.ecc.u-tokyo.ac.jp

Contents

Table S1. Nucleotide sequence of A-class genes from 'Baccarat White'.

Table S2. Nucleotide sequence of B-class genes from 'Baccarat White'.

Table S3. Nucleotide sequence of C-class genes from 'Baccarat White'.

Table S4. Nucleotide sequence of A-class genes from 'Duo Lavender'.

Table S5. Nucleotide sequence of B-class genes from 'Duo Lavender'.

Table S6. Nucleotide sequence of C-class genes from 'Duo Lavender'.

Table S7. Nucleotide sequence of Phylogenetic analysis.

Table S8. Oligo Sequences Used in this Study

Figure S1. Sequence alignment and schematic illustration of the putative insertion upstream of the *miR172* binding site in *PhBOB*.

Table S1. Nucleotide sequence of A-class genes from 'Baccarat White'.
This table presents the nucleotide sequences of A-class genes identified in 'Baccarat White' (BW).

>*BW-ROB1* 12737550
ATGTGGGATCTAAATGATTCTCCAGATCAAAGAAGAGAAATCAATATTGATGAATCAGAAGAAGGTTGTTCTTCACATAT
TGAACTAGAACCCGATGATGATAAAGGTAAACGGGTCGGATCTTTTTCAAATTCAAGTTCATCAGCTATAGCTATAGATG
AAATTTCAGAAGAAGAAGATGGAGAAAAAGGAAAGAAGAAAAGAAGTTCTCCTAGTAAACTTTTCGGGTTTTCAATGGTG
AGTTCGGGAGACTTGGAGCAACCGATAACGAGACAATTTTTTCCAGTTGATGAGGCTGAGGCTGAAACGGGTGTTGTAAC
TAATGGATCCCTGAATTTTCCAAGAGCTCATTGGGTTGGTGTTAAATTTTACCAAAATGAGCCACTTGGCATCACCGGTG
TGGTTGATGTAACTCAACAGCAGCAACAACAACAACCTATGAAAAAAAACCCGTCGTGGACCAAGGTCTAGAAGTTCACAA
TATCGTGGAGTTACCTTTTACCGGAGAACTGGCCGGTGGGAATCTCACATATGGTAAATCTTTATTTGTTTCATTTCACG
CTCCACATACTTCATAATTCATAGCTTACACAAATTAGGAGTACGGTCTACGTACATATATATCACGTTAAGATTATAAT
GTTATTATTATTGTTGTTGTAATTTATAATTAGCTCGATTTTTTTTAGTGCGTGTTTTTTCTTATTTGGCTTAATATTCT
TTCAGGGATTGTGGAAAACAAGTTTATCTAGGTATGTGATGTTCATTATATTTACAATTAAAGGTTAGTAATATCATTTG
CTCTTGTTGTATTCTTGAAAGTTAACTGATACTATTTTTAATGGGGTCTGCTTAGGTGGATTTGACACGGCACATGCAGC
AGCTCGGTAATCTCCTGGTTCTGCGCATTAATACGTTTGATGAATTTATTTTCATTTTCTCTCTTATTTATTACGACGAC
AAAATAATTAAATTATTACTGTATTTATTTGTTTTTGATAAATTATAAAAAATCGAACGTTTACAGGGCATATGATAGGGC
AGCTATCAAGTTCCGGGGAGTTGAGGCCGACATAAATTTTAATTTAGAAGATTATGAGGGTGACTTGAAACAGGTAAAAA
AAAAGTTATCAGATGACTAGCTAGGAAAAGGTGTGTTCTTTTTATGTTTTAGCATGTTTTAATTCTGCTTGTCTAGTGGA
GTACTTTTGTTTAATTCTTGCCTTATCTTAAACAGTGGTTTTCTTATCTTCAACAGTTATGTACTTTACATGTTTGTGTC
AAGAGTTGGTGGATATCATGGAAATGTAAGATATTTTTTTTTTTAAATTTTTACTTTTTTATGTATGATACAGATGACGAA
TTTAACAAAGGAAGAATTTGTGCATGTGCTACGAAGACAAAGTACTGGTTTTCCAAGAGGAAGTTCCAAGTATAGAGGGG
TTACTTTGCACAAATGTGGTAGATGGGAAGCTAGAATGGGACAGTTCTTAGGCAAAAAGTAAAAAATCCTATTCTTATAA
TATTCCTTTGATTGGTTCAATCCATTTTCTAAGAATAATCCTTTTATTGTCAATTATATTCTTGCTTTGCTAGATATTTA
ACTTTTTTTTTAATTTTTTTTTTTTAACAGGTACGTTTATTTGGGCCTCTTTGATACTGAGGTTGAAGCTGCCAGGTGTGT
GGTTGAAGTTACAAATTTATAATTTAATTTACATTGCTTGTTCTCAAATTTCTACAAACCTTAGTCCTTAAATTACTCTT
CCTTTTTTTAAAATTGTTTCTCCTATATTCAAATTTTATAGGGCTTATGATAAAGCTGCCATCAAGTGTAATGGGAAGGA
TGCGGTTACTAACTTTGATCCTAGCATTTATGATAATGAGCTAAACTCAACTGGTAAAATACCGTATCAACTATACTTTT
ATTAGACTTCTACTTGATAAATCATCATAAATTTTTGATAAAGTAAATTATAAAAAATAATCAAACTGTAAAATTTTAAT
TAATATTATTTTTCAGAGTCTACAGACAATGGGGCAGATCACAATCTCGACTTAAGTTTGGGTGGTTCAAGCTCAAAGAA
AAACAATCGAGAATTTGGGGATAATAGAGGTCAAAATCCTTCCTCAATGCAATTCGATGTTGATTGGAGGCATAACGGAT
TAAGGCCTGAGGTACTTGCATGAACGATATTATAGCTTAAAATTCTTGCACGAGTTTTAACTAATATACACCGATCATAT
ATTCTTTGGTAACGTTATACTAATAGTACAAAATTCTTTTTATGTTATCAACATATGTAAGTTAAATTCTCGTTTCCTTG
TGGATTTATCTTTTTTACCTCATATACAACAATGTTTTGATTAATTTCCAGAAGCAAACTGCACCAATTAATATGGATGC
TCGACGAAGAGATGATGGGTACAATGAATCAGAAACGTTGCAGTTATTGAGCCAAACGCACCTACATTCTCCAGTCTCTT
TGAAGCATAATAGTAGTCATCAATTACAACGGTTTGGCCAATATATGAGACCTGGTGAATCCCATATGAGTCAAATGTTT
CCACCACAATTCGCCTCATCAAATTATCAGGTAATTATCCAAGTTTATTTTAATTACCATACAGTTGCCTGAACTTGTCA
AAATTGTTCAGTAAGTATATATACAACTACATAAGTTCGTTTAGGTGTCTAAATGAAAAAGTTTGACAAGTTCAGAGGTC
TGTCTGTGTATTATGCCTATTTTGTTGGCACTCTTTTTAATTGTTGTACCATGATATGTACAGATTCAATTTCCAAGCGG
CAGCAATGGAGGTCGAATTGGAGCTACAAATGTAGGAGATTTATCGCTCTCGAACAGCAATGCTTCTTCACAATGGCAAT
CCAATTATCCTCCTCAAATATTTGCAGCTGCTGCAGCATCATCAGGATTCTCCCAGCAGATAGTAAGACCTCAAAATTGG
TCATCAGAAAATGGCTTCCATCACTCTCTTATGAGACCCTCTTGA

>*BW-ROB2*_(reversed) 12722124
ATGTGGAATTTAAATGATTCTCCGGATCAAAGAATGGAAGAAGAATCAGAAGAAGCATGTTCATCGCCAATTGAACTTGA
TGATTATAAAGGTAAAGGGGTTGGATCGGTGTCAAACTCTAGCTCATCAGCTGTAGTAATTGAAGATGGTAATAATAATA
ATAATAATTCTGAAGACGAAGACGAAGAAGAAGAAGATGATGAAAAAGGTAAAAAGAAAAGAAGTATACATAATAAATTA
TTTGGATTTTCTGTAATGGATCCGAATAATTACCGCGATTTATCGGAGAGTGAACAGCCGGTTACTCGGAATTTTTTTCC
GGTGGACGAGTCAGAAATGGGGTCTAGTACAAATTTACCAAGGTCACAATGGGCTGGAATTAAATTTTATCTTGAATCGG
AGACACGTGGCAGCACTGTATTGGCTGGAAACCCTACTGAGATTGTTGTTCAGCAGCAGCAGCAGCAGCCGCCAGTTAAA
AAGAGCCGTCGTGGACCACGGTCTAGAAGTTCACAGTATCGTGGTGTTACTTTTTACAGGCGAACTGGCCGGTGGGAGTC
ACATATATGGTGAGCACTTTTTGACATTTTATGTACACCGTTTTAAATAGTGACGGATTCAGAATTTAAACTTTATAAGC
TCGAACTATTGAAATTAGCGCACTTTTACATTTGTCCGTCAAATTTCGTGATACATTGAACCAAAAATATTGTGTTCAGT
TGAACATCCGCCTCGGTATGTTGATCAACATTTTCTTCCTACTATTCTCTCTTATAGCTGAATTTTTTCAATGCTTACTT
TCCTGACTTTGCTTTGTTTTGGTTTGTATGATTTCAGGGATTGTGGAAACAAGTTTATCTAGGTAAGTCTTGAAAATAA
TAAATGTATAGTTGAACTGAAGCTTTTAAGTTTATCAAGAATATTATTTGGTTGTTGTGTATGTTCTTGAAGATCAATCT
GATATTGGTGAATGGGAATTTTAATTTCCTCGGATATCATATGTGTAGGTGGATTTGATACAGCACATGCAGCAGCTCGG
TGAGAACTTGATTCTTGATTGTAATTTTTGCTCCAATTTTTTTTTTTTTTTTCAATTATCATTGTCTTATAGAAAAGAAAC
TAAAGATGAAATTTAAAAGTAATTTTTACTATTTTTGTGACCCTTTTTATTTCAGTGCATATGATAGGGCAGCAATCAAG
TTTCGTGGAGTGGAGGCAGACATAAACTTTACCTTGGAAGATTATGAGGAAGACTTAAAACAGGTATGTTTTTATTCCAT
GTTGTTCTAATCTTTCCAAAATGTCTCTGCACTTGTGTCGGATCATTGAAATTGTGCATTTTTTTAAAGTATCTGACATG
GGGTGACATTATTTTGGAGTTGAAATTCACCCTCATAAGTCATACCTTATCATAAACAGTTGGCTTCCTTATCATCAACA
GTAGTTAGGTTCCCAAATGTAGTGTCAAATTTCAGTGAAATGAATATACTTTTCTCTCCAATAATTTGTTTTATCTTTTT
GTGGTATATGTCACAGATGACCAATCTAACAAAGGAAGAATTTGTGCATGTACTAAGGAGACAAAGTACTGGTTTTCCAA
GAGGAAGCTCAAAGTATAGAGGAGTAACTTTGCACAAATGTGGTAGATGGGAAGCTAGAATGGGACAATTCTTAGGCAAA
AAGTAAAAAGAAAAACAATCTTATCTTCATTTTATTCTCTTGTATGATCAAATAAATTTTCAAAAATGATCCTTTTGTTG

TCAACTACACTAATATTCCATTCCTTTCTTGCTATATTGACTTTTCCTTATCTATTTCTTTCTAACAGGTACGTTTATTT
AGGTCTCTTTGATACTGAAGTTGAAGCTGCCAGGTGTGTGATGTTCTTCTTTCTTTACTCTTTTAGTTCTAATAATGGCG
GCGTCCAGATCAACCTGTAGGCTCCTCAACTATTCCACCAAATATTTGCTAGCTTTTATTAGCATATGTACTGCGTAACT
CTTACCGCCAAGGTTTAGGCACATAAGAACAAAATCACCGAGTATTCAATCTTACTGGAATTTGAACATATATTGATTTC
CGAGGTTTTCACCCGCTTCATTGACCAAACCACACCCATGGGGTGCCTGCTGTTGCAATTCTTGCACTCAAACTACTATT
ATTAGTGAGATTGAGCCTTGAGGGCCTTGTATATTTTAACTGTTCTATGGACTAATTTTTACTTTCTTTTTTCTCCTTTA
TCTTTATTTAATAGGGCTTATGATAAAGCTGCTATCAAGTGTAACGGGAAGGATGCAGTTACTAACTTTGATCGTAGCAT
TTATGAAAATGAACTTAACACAACTGGTACATTACACTTTTCTTTGCTAGCTTTTTGATTTAATCTTTGTCTGCCAACAT
ATTACTGGATAGTATATTATTAAAAGAAAAACCTAGAATTCATAAATGCTTCTTTTTTCAGAATTTACTGATAATGCAGC
AGACCACAATCTTGACTTAAGCTTAGGAAGTTCAAGTTCAAAAGAAGGCAGTCGAGAACTAATGGGAGATAATAGGGGTC
AAAATTTTCAATTAGATGTCGATTGGAGGAACCAAGGGTCAAGGCCTAAGGTATATACTTTTATATCTATAATGTATTTT
AGTGGCGGATCTAAAGTTATATTTACGGGGTTTTCAGAACCAAAAACTTTGACCAAAATCCTATATTTATAGTTTAGAAT
TCATGAACGTAAAATCTTAGATCCGCCCCTAATGTACTTTAGACAATGGCGTTTTTATTTTCACATATTACTCACATCAT
GCTCTAAATGTTTTAATTTCCTGCAGCTACCTAATCAAATTGGGTTTGATACTCAAAGAAGAGGTGGGTACAATGAGTCT
GAAACCTTGCAGCTCTTGAGCCAAACACACATACATTCTCCAGGCTCCTTGCAGCCTAATATAAATGAAATGCAAAGGTT
TGGCCAATTTACTAGAAATGGTGAATCCCACATGGTTCAAGTGTTTCCTCGACAGTTCAGCTCGTATCAAGTGAGTTCTA
ATCCTATCATTACCTCGTTGTCGAGATATAATATAATTAAGTAATAAAAGTGATCTTCACATCTTAAATTTAAACAGTTT
TAGAAAGCACATATTTCATTTCTTAATTATTTTACATCTCAACACTCTCCTTACATACATGTGTGCCTAATTCGTTTACA
TTGATCAAATTAAACATGCAAATATATTTTTTGCTTTTAGAGTGGCATTCAATTTAATCCTAAGACTCTTATCTCCTCTG
ATATCATGTCAAATTATGCGTGGTTAAGTTGTTAGAGAAAGTATAATCCTAATTACTCTCTTGGTTCCACTTTAAAATAG
CACTTTACTGATTGGAGAGTTAGCCAACAATTTTTCTCTAACTACAATTTATCAAATATCACTTGAATATTGTGAAATAT
TAAATATTCAGACTTATTTGTAATTTTTATTAACTTCTTAATATGTAGTAATATTTTTCTTTAAAAACTTGAAGATTTTA
AGTCAGTGATTGACTTCAAACTCATGAAATCACATAAAATGAAATAGAGGGATTACTTTATTGTATCATGTCTTAGCATT
ATTGAGTTTTTATGTACCATCCCATGTTTTAAAAACTTGTATTGTATACAGAATCAATCGCCAAGCAGCAGCAATGTAGG
CCAAATTGGGACTACAAATGCAAGAGATGTGCTTTCACTCAGTACAAGTAATTCTCAAGAATGGTACCATCCCAATATTC
CTCCTCATCATCATATATTTGCAACTGCTGCAGCATCATCAGGATTCCCCCAGCAGATAGTAAGACCTCAAAATTGGTCT
CAAAAAACTGGCTTCCATCACACTTTCATGAGACCCTCTTGA

>*BW-ROB3*(upstream)
ATGTGGAATCTAAATGATTCTCCAGATCATAGAAAGGATGATGAGTTATCAGAAGAAGGAGGTAAACAAGTTAGATCGGT
TTCAAATTCAAGTACTTCATCAGCAGTGGATAATATTGAAGATGGTACTAGTAATTCATCTATATCAATATCAAGACAAA
AAGGTAAAGAAAAGAAAACTACTACTACTACTCATAGTAATAAATTATTTGGCTTCTCAGTTATTTCTCCTAATAATAAC
AACAACAACAACGACAACTTGTCGTCGGAAAGTGAACCGCCAGTTACCCGGAACTTTTTTCCGGTTGATCAGTCAGAAAT
TGGACATGCAACTGCAAGTACATTTGATGATAGATCATTTCCAAGGGCCCATTGGGCTGGAATAAAATTATGCCAGCCCG
AATCACCAGGAAATTCATCGATGGGAAAGAATACTGAGTTGTCGCAACAAGTTCAGCCTATGAAGAAGAGCCGGCGGGGA
CCAAGGTCTAGGAGTTCACAGTACCGTGGTGTTACATTTTACCGCAGAACTGGCCGGTGGGAGTCACATATAT

>*BW-ROB3_1_*(downstream) 12214246
CAGAATCTACTCATAAGGCATTAGATCACAGCCTTGATTTAAGCTTGGGTGGCTCTAGCTCAAAGCAAAGCAGCAAACAA
TTAACGGAGGATAATGAGGATCAAAATTATTCTTCTGTCCAATTTGATATTGATCGGAGGCACCAAAGATTGAGGCCTAA
GGTACCTACATAACTTTTCTAGCTTAATTTCTACCAAACTCTCTTGCACTTTTTCTTCTGCATATTATGATAAAGTTTAC
TAAGGGTTAGCTGGTGCACGATTTTAATATACGATGGATAATTAATAAGCATACATGTCTATCCATTTTAACTTAAATAT
AAGACATTAGGCATGAAGAAGTTCGAACATGTGACATGCATCTAACTCACACATCATGTGTTATGCTCATAATTATCGCA
AGGTGAAAGTGCTAAGGCACCTTGTAGTTGGTGCATTTATATTGATACCCCTTATGTTCCAATTTTTTTGGTCATAGTTT
TACTTGATTTGCGAAATTTAAGAAATGAATGAAAATTTCTAAAATTTATTGTTTAAAACATGACATCGCATCATTTTTAT
GGCTATAAAATTTTTCATTAAGTTGAAAGTGATAAGTTGAAAGCTATATTGTTCCAAATTAAGAAACCGGACATTCTTTT
GGAATAAACAAATTAGAAAATTGTGTTTTTCTTCTTGGGTGGATGGAATAATTAGTAGCATTGTTTTCCTTTTTTGCTC
CTTATACATGTTTTGATTTCAGCAGCAAACTAGTCCAACACGAAGAAGAGATGGGTACAATGAGACGGAAACCATGCAGC
TCTTGAGCAAAACGCACCTACATTCTCCGGGCTCCTTGAAGTCAAATAAGAATGAAATGCAAAGGTTTGGCCAATATATG
AGAGTTGGTCATGAAGCCCAAATGATTCAAATGTTTTCACCACAATTCAGCTCGTCAAATTATCAAGTAAGTGCCCATTT
CACATGAGTTTAATTCCATGCATTGACACTACATATAGGATAACTATACAACTAGGTCATGGCTTAAAAAAATAGTAACT
ATAAATAACTCTCCATAATGAATATGATTAATTATGTATCTAAGAAAATAAGACAGATAACCTGCTAAACAAAATACATT
GATAAGATAAAAATTCTTTACATATGCATGTATAAAAATAAAGAGTCTAACCTCTATAATACAATGTATTTTAGGTTTAC
TAGAGATAAGTACTTGTACCACCCATAAGAACTGGGAATCTTAACTTGGAAAATAAGGTAAAATTACCAGCTACTTACAG
CTCGTTAAAATTCAATATTAGTCAATAGTGTAAAATTCCTTATATGTATATAGGTTTTTTTATTGTTTGATGCTATACTA
TAATTGCTATTGGTATCTGTACTTTTTGTCATTATTAACGTAGTTGCACTAAGAAAGTTTATTTTATCATCCTTGTAAAG
TTTGTTCAAATTGTTATATGATACAGAACATTCAGTTCCCAAGCAGCGGCAATGTGGACAGATATGCTGGACCTAATACG
AATAGAAGAGAGCCTATGTTCTCATCAAGTGATACTCAACAATGGCAATATTCCAATACTGTTCCTCCTCAGCTATTTGC
AACTCCTGCAGCATCATCAGGATTCCCTCAGCAGATAGTAAGATTTCAAAATAATTGGTCTCAGAAAAATGGCTTCAACT
ATTCTCTTATGAGGCCTTCTTGA

>*BW-ROB3_2_*(downstream) 12189066
AGCAACCTAGTCCAACACGAAGAAGAGATGGGTACAACGAGACGGAAACCATGCAGCTCTTGAGCAAAACGCACCTACAT
TCTCCAGGCTCCTTGAAGTCAAATAAGAATGAAATGCAAAGGTTTGGCCTATATATGAGAGTTGGTCATGAATCCCAAAT
GATTCAAATGTTTCCACCACAATTCAGCTCGTCAAATTATCAAGTAAGTGCCCATTTTACATGAGTTTAATTCTATGCAT
CAACACTACATATAGGACAACTATACAACTAGGTCATGACTTAAAAAATAACTATAAATAACTCTCGATAATGAATATGA
TTAATTATGTATCTAAGAAAATAAGACATATAATCTGCTAAAAAAATACATTGATAAGATAAAAATTCTTTACATATGCA

TGTATAAAAATAAAGAGTCTAACTTCTATAATATAATGTAATCTTAACTTGGAAAATAAGGTAAAATTACCAGCTACTTA
CAGCTCGTTAAAATTATATTAGTCAAATAGTGTAAAATTCCTTATATTTATATAGGTTTTTATTGTTTGATGCTATACT
ATAATTGCTATTGGTATCTGTACTTTTTGTCATTATTAACGTAGTTTCACTAAGAAAGTTTATTTTATCATCCTTGTAAA
GTTTGTTCAAATTGTTATATGATACAGAATATTCAGTTCCCAAGCAGCAGCAATGTGGACAGATATGCTGGACCTAATAC
GAATAGAAGAGAGCCTATGTTCTCATCAAGTGATACTCAACAATGGCAATATTCCAATACTGTTCCTCCTCAGCTATTTG
CAACTGCTGCAGCATCATCAGGATTCCCTCAGCAGATAGTAGGATTTCAAAATAATTGGTCTCAGAAAAATGGCTTCAAC
TATTCTCTACTGAGGCCTTCTTGA

>*BW-162Scf00072g00229.1*_(reversed) 12716442
ATGAAGAAAGTGATGTTGGATCTTAATGTAAGTATAATCAATAATTACATACTTGATCAGAACCTTCCACAAGTTTCTCC
ATCATCAGGAACCTCCAATTCATCCATACAAAATGCAGAGGCAACAAGCAGCGTCGACGACACGTGCTCTACACGCGCCG
GTACCTCGTTCGTGACTCGGCAACTCTTTCCTATTGAGTCGAACCGGGAACAGGACTTAACCCGGTCTGATCGGGTGAAT
TTCACTTCCGGTTTCGGAAACGTGGTAATAGTACAACAACAACAGCAGCAGGAACAACAACAACAGCAAGCGCATGTGAA
GAAGAAGAGTAGGAGAGGACCAAGGTCAAGAAGTTCACAATATAGAGGAGTTACTTTTTACAGGAGAACTGGTAGATGGG
AATCACATATTTGGTTAGTTAGTTAGTTACTTAATTCTCCAGCTGTAATTTTTTTACTGCATTTTATTTATCGTCGTTT
TATTTAATATAATTAATTTTGGATGCAGGGACTGTGGGAAACAAGTATACTTAGGTATGAATATTGCTATTTGAGTTTGA
ATTGTAAATAAATTTTACACTATCATGTCAGGTTTACCTGTCGACGGTTGGAACAGTTAACCTGTCTTATTATTCAAGTT
TCATTATGCTTGCTATAGTATTAGTTATTACAATCTCCATTTCTGTTTATGTTACCTTTAAATAGTCAATCTCCATGAAC
TTTGACCAATATTCTAAGATATGTCTTTCACCATGTTAATATAAGAAGAGTTGCAACCTATAGTATTTCTCGTATAGTTT
TGCATATCTGAATTTTAATTATAAAATATTAAACTTATCTTACCCAATTTAGCTTCAAAGATTAGCCACTTGACTTTCAG
AAAGGAGAAAGGTCACATAAGTTGGAAAGGAGGAAGTACCAGATTTAGTTAATTTCCTTGTTTCTGTGGATTTTGATGCT
AAAGTTATTATTAATGTCTCTACTTTTTTCTATTTTTATTTTGATTGGTTTTTGTTGTTGGTTTATATTAGGTGGTTTTG
ATACTGCTGATGTTGCAGCAAGGTAAAGTGCAAAAGCATAGTTCGCAAGCATGCTTCCAGTTTTACTTTATTTCCCGAAT
AAATTGTTCCTTGATTTGATTGTATTGGTTTTCTTCTCGATTTTATAGAGCTTATGACAGAGCTGCAATTAAGTTCCGGG
GTGTTGATGCTGATATAAACTTTAGCATAACGGATTATGAGGAGGATATGAAACAGGTTAGAGATACAAAGATTGAATTT
TTGCAAATATGTTATGGTCACATTTGAACCTTATTTCGAGGCGGATAGAGAATTTGAATTTTATGAGTTCGAGTTCTGGA
ATCTCCCACGATCCATTTGATTTATTAGGTTTGAAATCAATTATCTTTACTTGATGATTTTTTCAACAATACACCGGGTC
TGAGCCAACACTAGTTGGTTCGAAATGAACCCATAGCTTATACTCTATATCTGCTTCTGTCTTTGGTTTCATATTATTTC
TTTATTAATTGGGGTGCATTATATGAATCATTTAAATTCTATTCTGTATTCATTGGCCTTATTTCTGGACTGATGCAGTT
GAAACACCTTGGTAAAGAAGAATTTGTTCACGTGCTACGACGCCAGAGCAATGGTTTCTCAAGAGGGAGCTCCAAATTTA
GAGGTGTGACACTGCATAAATGTGGCAGATGGGAGGCTCGGATGGGGCAGTTCCTCGGCAAAAAGTAAGGAACTAAAAAT
TGAAATTCTTGTAAGGGTAGATTACAGCTTAGGTTATACTAGTAGTTTGTCCAAAGTGGGAAATGATTCTGTTAAACACG
TGTATTTGAAAAGAAATCGCTGGTACTGTGGTATTCATGGTGGGGATTAAACTGCTTACATTGCAGGTATATATATCTTG
GGCTATTCGACAGTGAAGTAGAAGCTGCAAGGTCCTAAGATCTTGAATTACCCTCTCTGATAATGAGTTGTTAACCTAAA
CTCTCAACTTCAATCTTGTGTCATCCAACCTCTATCTCCCTTTTCCTTTTCTGCAAAATAGGGCTTATGATAAGGCTGCA
ATCAAAACTAATGGAAGAGAGGCTGTTACCAACTTTGAGCCAAGTGCATATGAAGGGGAAACAATATCTGAACCTCAGAG
TGAAGGTTTGCCCAAAAGCTCTTGCTCATTTCCAAACTAGTGCAACTAATCCGAAGCAACATGCATTACTATACCTTGAT
CTATTGCATTTGCAGGTAGCCATCATAATCTTGATTTAAACTTGGGGATATCGACCTCTTCTTCAAAGGAAAATGACAGG
TTTGGAGGGAAATACTATCATCCTTATGATACGCAAGATTTAACAAAATCAAAGGTATTAGCAGAGTAGCTTATACGTTT
CTGTTTGTGCAGATTGATTTGGATTAAAATGTTCTCTTTTCTGTTATAGTCTTCTTATTTCCTCTTTTTCTAAGTTCTTA
TGCATTACAGTAGATTCATATCCGAAGAAATTTTATCCATAATGAGACCCTTGTTAATTCATAACTGTGATGAAACATTT
CATGAACATCTGTAATCTTTCTTTCTTGTAGATTGTAATAATCATCATTACTTGTAGATTGTAATAATCATTTTAAGCTC
ATTAACCACAGATGGATAAACCAGGTCCAGTAGTAGTTGGAAGTTCATATCTCAAGGGACTACCAATGAGACAACCTCAC
TTGTGGACCGGAGCCTATTCTAGTTTCTCTCCCGGCTATGAGGTAAGATATCGACTCTACCCTCAGTCAATGATTCTGGG
GCCAAAAGATTTCATGAAATTCCCAAAGTTTAGCTCTCTTTAGTTTTCTTTTTCTCTCTTATGGTCAAATTTGTTTGTCT
TCAGTTGCTAAATTAGCCATCTTCATGCCCCCATGTTTCCCTCAAATGCTAGGATCATTTGTTTTGCATGAGACATTTCA
CTTTTTTTTCTTCTCCTTTTNNNNNNNNNNNNNNNNNNNNNNGGGGGGTTGAGAATCAGTTTATAGTGGCTTGGTAACAAGA
ATTTAGATGCCCAATGACAAAATTGATCTTTTTAGTAATGTAGCAATATTTACAGAAACCACTTTCCCTCCTATCGAGGA
AGAATAAAAAATACCTTATCAAAAAGTAATGTGGCAATATTTGTACTTTGTGTCCCTTCGAGGATATCCGATAAAAATGG
GAGTAATATTTGTACTTTGTGAGTTTACTGGGATGTCAGCTTGTTATTCTTCCCGTGCATCTCCTGTGGGATCATAGTCA
GTGCATAAATGCAACAAGTGGTTATCTTAAGGTAAACAAGTGTGACAGATCAATGAGGAAGCTCCTGCTTAACAGATTTC
AATGCCTCTGTTATGCCTTATTTTACTACTTCCTTAAATTGAACGTATCCCTGAGATTAGCCTTCCAAAGGATCCAACTC
TTACACAAGTTGGTACCTAGAGTTTGTTAGCAGACTAGTATATATAAAACTTGATGATGATGGCATTTTCTTCGCTTTCC
TCTATATTACTAGTAATACTAGTCGTGGGTTTAAATCATATTACGACAGTTAAAAGCTTCATTTTTTCATTAAACCCTTG
GCAGGGAAGAGCATCTGATAAGAGCAAGGATATAGGTTCATCTCAAGGACCCTCAAACTGGGCATTGCAAATGCCTGGTC
AAGGTGGTACAGCACCAATGACAATGTTCTATACCGCAGCATCATCAGGATTCGTAACACCAGCTACTGCTTCAGCATCT
CCAATGGTACCCTCAACAATTGCGTCTCAATACTATTACCAGATCAGCTCCCACATACCACCACCATAA

>*BW-162Scf00389g00028.1* 12384905
ATGATGTTGGATCTTAATTTAAGTGCAATTTATGATGAAAAAGTAGGAGAAATTGCCGTAGCTGATGAATCGGGAACCTC
CAATTCATCGGCGAGGAATGCAGAAGCCTCCAGCAGTGCTGGAGATGATGACTCGTGCTCCACACGCGCTGCCGGAGACT
TGTTCGCCTTTAACTTCGATATCCTTAAAGTTGGTGGAGCTGAAACTAGTAGGAGTTTCAGTATCAATAATGATGAGGAA
GTGTATGATGAGAATCATATGAGGATGGCTCGGCCCGATGTTGTGACTCAGCAGTTTTTCCCGGTAGATACTGCTGAGTC
TAATCGGGCCCACCATACCTCTCGGAGACCTGATTGGGTGGATCTTTCATACGACCCACCCAATACTCTTGGTTTCCGAG
AAGTGGGAATAGTACACCCACAACAACAACAGTACCAACAACAACAGCAGCAGCAACAACCAGTTAAAAAAAGTAGGAGG
GGACCTAGGTCCAGAAGTTCACAGTATAGAGGTGTCACTTTTTACAGAAGAACTGGTAGATGGGAATCACACATATGGTT
TGTATGCTTTAACTATGAAATGTTTTAAATTTCTTAATATTGTTAAAATTATTAATATGTGGGTGTTTTTTTTTTTTTT

TTTTTTTTTTTTTTTTGTATAGGGACTGTGGCAAACAAGTATATTTGGGTATGTACTACAGTATGTAATATTGTTATTTT
AATTGTTATCTAATTGGTTAAGTTCATTGGTCTGTGATCTTTTGGAGTTTAGTTATGCATATGCTATTTCTATTTTGGGA
AATTTTCTGATTGGCTTTGTTTTTGGCTTGTTTTTAGGTGGATTTGACACTGCTCATGCAGCTGCTAGGTAAAAACATAA
ACAGTTTATATACGATATCTCTAATATTTTATAGATCCTTTCAGTTTTATGCTTTTTCCCAAGTTAAATTCTATGCTTGA
ATAGTTGCATTGATGAAGTGTTTCTCGATGTTTTCTTCGTGATATCAGAGCTTATGATAGAGCAGCAATTAAGTTTCGGG
GTGTTGATGCTGATATAAACTTTAACCTAAGTGATTATGATGAAGATATGAAGCAGGTTCGAATGTAAAGATTTTAACTA
GTACTATAACATGTTAAGCTCATTGTTAAACATGAATATGCATTGTACTGAATTTGGATTCGAATTGTTTGTGAACTGAT
GTAGATGAAAAACCTAAGTAAAGAAGAATTTGTACACATGCTGCGACGCCAAAGCACTGGTTTCTCAAGAGGGAGCTCGA
AATACAGAGGAGTAACGTTGCATAAATGTGGAAGATGGGAGGCTCGGATGGGGCAGTTTCTTGGCAAAAAGTAAGAATCT
TTATTCATTGAAATTGTTGTAGAAGTAGAAACTTTATTTATTGAAATTGTTGTAGGAGTAGTTTCAACTAATGTTACTCA
AATCCTTCGGAAGTGGCGTTGTACCCGTGTCGGATTCTTAAAAATGAATATTATTTGAAGATCTGACACAGGTGCAGTGG
CACTTTGTTGGAGGATTCGAGCAAAATAAGGAGTACATTACATCTTGAGAATATGTAGTTGGTCTGAACTGGGAGACAAT
CTGTTAAGCATGCCTATTTGAAGAAAGAAATCAACGGGACTACAATATTCATAGTGGGCATTCCACTGCTTACATTGCAG
GTATATATATCTTGGACTATTCGACAGTGAAGTAGAAGCTGCAAGGTCCTAATGATCATGAATTTACCCTCTTCCCTGAT
AATGAATTTATTACACTAAACTTCTCAACACAAAATCTTGTATCAACCAACCCGTATTTTCTGCAAATTAGGGCGTACGA
TAAGGCAGCTATCAAATGTAATGGAAGGGAAGCTGTTACCAACTTTGAGCCAAGTACATATGAAGGGGAAACAATCTCTG
ATCCTCGGAGTGAAGGTTTGTTCACAAGCACTTAACATGATCATTAGTTTTTTTATTATCTTTTCATTTTAAATATGTGT
ATCAAGCAGGTAGCCAGCAGAATCTTGATCTGAACTTGGGAATATCGACCTCTTCTGCGAAGGAAATTGAAAGGTCGGGG
GGTTTCCAGTATCATCCTTATGATATGCAAGATACAACAAAATTACAGGTTTAAATAGAGTAGTTTATTTTGAAATGTTG
CAGCAGATTGAATTTGATGGTGTTTTACTTCCGAGCTCTTCCTTTTATTTCTTTATACAGATGCAAGAGACTGTGAAACC
GTTAATCTTGTTTTTTTAGATTGTGCTGATAATTGGTTTCCTTTTATGCAGATGGACAAATCTGGTCCAGCAATAGTTAG
TAGTTCACATTTTAAAGCACAGCCAGTGACATCTGAACAAGCTCACTTGTGGAATGGAGTATATTCTAATTTTTTTCCCA
GCTACGAGGTACAAAAACAGTTCACTTTATTCTCTAGCTCTGTTATTCTTCTTTACTAGTTGAAACTTTCGATTTTCTAA
AACTTTGGACCGACGTGGGCTTAGTAAGAGCGACATATTCTGTGAGGATTCATGAAACCGAACACATGCTTGGATTTGAG
GCATAGTAGTAGTTGTTGTACTTGTACATGTGCCTTCAGTCCAAAAGAGCCATCTGCATGACCCCCTTGTTTTCTACATT
ACAGGATTCTGTCATTTATGAAATAGTTGGTGGCTGCAAGTCAATACAGTGTAGGTTCTGATTGTTGTGAACTCTACTGA
AACTCTGTTGAAAATTCTTGTAAACATAAGATTCTGCATAGGTTTTGTGAGGGCCTTTACATTCTTTCGACTGTTTTCCA
GAGGGTAAATTGCCATTGATAGACATTCTATTCTTTGTGGCTAAAGCATTTTATTTAACGGATTTAAATCATATGCCCTA
ACATTGTAAGCTGGTAAGGCTCTGGTCCTTCCCGACCTTGATGCATAGCCGGGAGCTTCGTGCATTGGACTTCCCTTTTA
TACTCTAACAACATGAATAGTATAAACTTGTCATTGAAGTTTAATTTCAGTTTTTTCTAGATGGTTACCAATCAATGCTC
GGGCTAATTAACTAAATGGCTTAGCAGGAAAGAGCGTCTGGGAAGAGAGTAGAAGTAGGTTCATCCCAAGGACCGCCAAG
CTGGGTAATGCAAATGCATGGTCAGGTCGGAACAACCCCAATGTCAATGTTTACTGCTGCAGCATCATCAGGATTCTCAT
CTCCGGCTACCATTGCTTCGGCCTCTCCAATATCTGGTCCTAACCCGAATATTCACAATCTATCATTTGCTACATATTCA
ACACCATCAACAAATACCTCTCAATACTTTTACCAGATCAGGCCGCCGCTACCACCTCCATAA

>*BW-162Scf01024g00326.1* 12556053
ATGTTGGATCTGAATGTATCAGCAGTTTCTGTGAACTCAAATTGTGATGAAACTGACCCATACAACAATACCAACACGTT
CTTGAAAGATGATATTTCTGGAACTTCAAACACTGATTCTTCTTCAGTCGTCAACGCTGTTGTGGGAGATGAAGATTCCA
ACAGCTCTTCACATCAGCATGTGTTTAAACATTCTCTTTCAACTCTGAGTTTCTCCATATTGAAAAGTGATCGTGTTATG
GAAACTGAAGATGACATGACCAGTGATGATTATAAAACAAGGCAGCTTTTTCCAGTGAAAGTGGAGACGAAGATTCAAGA
TCAAGCTCAGTGCTGGCTTAACTTGTCTGTGCCGGAATCCCGGGGAGGGGCAGATATCGGAGTATACAAACCACCGCCGG
CCAAGAAAAGCAGACGGGGGCCTAGGTCCCGTAGCTCACAGTATCGTGGTGTCACATTTTATCGGCGAACTGGAAGATGG
GAATCGCACATCTGGTACTTAATTCTACATTGCACTAAACCATATTCATTTGTATATGATTAAGTGATGAATTGAGGAAA
AATTGACTTACAAAAATAGGAAATTTATTACTATAGGAAATTAATTATAGGAAGGGCAGCCTTGGTGCAACTGGTAAAGT
TGTCTTTGTGTGACCTAGATAGGTTACGGTTCAAGCCGTAAAAACAGCTAATGATGAAGCGTATATCACACCTCTTGAGG
TGCGGCTTTTTCCGGACCTTGCGTACATCACATCCCTTTGGGGGGTGTGGCCCTTCCTCCGACCTTGCGTGAGCAGAATG
CTTTGTGTCCCTGACTGCTTTTTTTTTTAGATGTTAATTTAGAATTAACTATGATTATTAAGTGATAAACCTTATGAAAA
TTATTAATTATATTGTAAACCTAGATAAAATTTATAACTTATCATTATTAAGGGATTTAATGCGCTCAGTATCTATATTA
ATTAACAACTATGATTAAGTGAGAAAATAACATGCATCTATTAATTTTGTGTAATGATGCGGATGAGTTTGTTTTCACTT
TGATTTAAATCCTTTATTAATTAATCATCATCATGTGATAAATTGTGTATAAAACTTAAATCATATACTATACTACGGTT
TAGTGATAAGCCTTCTGCAAGATATTAGTTGTTGTTTCGATGAATGTACCAGGTGGGGAAAGTATTTCCAGTGCAGGTGC
ATACATGCATACCTTACTATAGGCCTTATGAATATAGTTATACCTACAAATCTCTCTTTTCTGTTCATCTGTGGGTATGT
ATGTAGTTGTGCATTTATGCACAAAATTTCATATAGCCGACCCATTGTTTGGGATTGAGGCTTAGTTGTTGTTGTTACGA
GGGGGATTAGTAAGAATTTGTGAAGTCGCATAACGATGGACCATCAAATTCCAACCATTTTCAAGTAATAAGGTACAAAA
AAATGAAGCATGAAGTCCAACTTGACCTTCAGTACGTCTGTACTTTATTTGCCTTTTTATTTAATGATGCTTCATTTTAT
GATGATTTGGATCCCATAGGTTCTATGTGGTATTCTCATTGGCCTTTTTCATGATTACTTATTGGGGATTTTACATTCTA
TGTGCAGGGATTGTGGGAAACAAGTGTATTTGGGTAAGTGTCTATCTAAGCTTAAAGTTAATTGAAGACAGTTTTGCATA
TTGTTACTGTTCTGTTAATTGTATAAGGGATTAAACCTTTACTGGGATGTTGGTTTTTCAGGAGGATTTGATACAGCTCA
TGCTGCAGCTAGGTAAGATTTTATTCTCAGACCTAACAATCAATTTTGTTATGCTTAATTAATCCTTATCATGCAGTATA
TCTATATAATTTCTTTATTCTTTTGAATGATATAGGGCGTATGATCGAGCTGCAATTAAGTTTCGGGGAGTTGATGCTGA
CATCAATTTCAGTATAACTGATTATGAAGAAGATATGAAGCAGGTAGAATAATGTGGTGGAATGCAACCAATAACTGTGG
ATATCATGAGAGTTAATTAATTTGAAAGTTGACCTTTAATAAATTGATTTGCAGATGAAAAACTTGTCAAAAGAAGAATT
TGTCCAAATCCTTCGTCGTCAAAGCACTGGATTCTCTAGGGGAAATTCAAAATTCAGAGGGGTAACCTTGCAGAAATGTG
GTCGATGGGAGGGTCGCATGGGTCAATTCCTTGGGAAAAAGTAAGGATGCCCCAAAACTAGCAGTTTAAAAGTATCAATG
CATGTAACTTGTATTTCTAATTTGGTTTTGAAGGCCTGGTGTTTCAGTCGCTTTTTGTTTCAAATTCTTGCCTAGATTAC
AAACTCATGGTAGTTAATTATGTGCGAAAAGTTGATAGGACCCTAGTTTGTAATCTTGCTCCTACAAGATTACAATGCTT
GGCTGAAGGTATATATATCTTGGATTATTTGACAGCGAAGTTGAAGCTGCAATATCCTTGTGATCATGAGTTCATACCCT

TTTGAGACTAATTGTCTCACCTTTAGCATTCTCATTTTCTGAATTACATCCAACCAAATATGCATTTTCCAATTTTGGTG
CAGGGATTATGACAAAGCAGCCATCACATGTAATGGAAGGGAAGCAATTTCCAACTTTCAACCTAACACGTATGGACGGG
AAATAAAGGAGGATAATAAAGATGGTGGTAAATCCATCTAACCCTTATCTCTTATTCTTGCAACTTCTGTGGTGGCCATT
TGGAAACCTACAGAAAATAATAATTTCTCTGAACATGGATGTAGGTAGTGGTGAGAATCTTGATTTGAACCTTTGGATTG
CTCCACCTTGGGAGGGGCCAAAGGGCGATGAAATTGGTAGAAACGTGCACTTCAAATTTGGAGCCGGTGAAATGGTTATT
GGAAAAAGACTCGAGGTATTTTCTGATTCAAATACTTAACAACTTCTGTTTTAGTGAGGCCTCCATTATGGCAAATTCTG
TGCTCTCTCTCTCCCCTCCTCTCTCCCTTTCTCTCGGCTTGAGCATTTTGGGTCTGATCCATTCAGATGTTAGTAACAGT
TATGGCTACTCTTTGCTCTGTCAAATTGTTTTATGAACACATACAGTAGTCTAGACATTTTGAAGTTATTGCTGAATCTA
TAGCATGATTTTGCATTCTCTTATTTTGTCAATCTTCGTTCTTTTTTAAGTTTCCTTTTATGAACTCTGCTTTTGCCAAT
CTTACAGATTGAAAGCTCCTCTACTGCTCCACAAGGTCCACCAACAGCATCTAAGTGTTCCATACTGACTGGAATGTATC
CTGGTTTTTCTCCACATAATTGGGTAAGTATTATGTTCTTCTCCCGATATAAGTAAATTTATGTTATAGTGTCGAGCGTA
ACTGTTATATGAAATAACACCAGGCAATGCTTACTTCTGCCATCTGCTAAGTTAAGATTTTGAAAGAATGGAGAAGCAAC
TTGTTTAATAATAGACTTGTAAACTCCATTATGGTACACATGGGATTTGTAAACTAAGCAGACTTTTGTTTAGCTATCCG
CCCCACAATTATTCTCCCCTTGCCCCACACCCCTTCTCAACAATATTTCCTCTTCGAATTTATTACTCCACCAAGCATAA
TAATTTCAAGACTGTTTCTTGCACATAGTAACATTTATATTATTCTGGTCCAGAAACTACTGAGGGCACTACAGTATGAC
CCTTAAACAAAATATTTCCTTGTCTCCTTGATTCATTATCAACGATGATTGACATGGATTTAATGTTTTTGGGGATACCA
TATGATTGTTATGCTTTCAGCGCTCCAACTTTTATGCTGGAAATACTTACATTGACAGAGATGAAATTATTTCATCATTC
TTTCAAATTACTTCATTTTTTATTTGAACAAAACCTGATGGAGATATTTCTTATTGTTAACAGGAAGGAGGAATGACGAA
GGCTGAAGCAACTTCCTCTCCAGGATTCCCAAATTGGGCATGGAAATTTCGTAGCCAGGGTATGGCCACTCCAGTGCCAG
TGTTTTCTTCTTCTGCAGCATCATCAGGATTCTCTACCACTACCACTCTCTATCCAAATTCCCTCCTACCGCCAAGCAAC
CAACTACGACTCCCCACAAACTCGACTTTCCCACACCCTCCAATGACCCTTAACATCAGTTAA

>*BW-BEN* 12726803
ATGTTTGACCTTAATCTTTGTTTCGAAGATGAAGAAGAATTACAATTCGATAATCATAATAATTCAACAGAAACATCAAA
TAATTCTTCTTCAATCATTAATAATATTGAAACAACAACTACTAGTTCAACTTGTGATGATCATGACTACATTTCATATT
CTAATAATAATAATAATAATAATTCATTTGTTGATTTTCTAAAAACTGACAATGATCAGTTTTTAGTTACTAAGGAATTG
TTTCCGTTGAGTAACGGCGGAGAAACGGCGGCGACGGTGAATGTGTATGGTAATTACGGTGGTAGTATGGAACAGAGGAT
TATTGTTCCAGTACAACAGCAGCAACAGCAAGTGAAGAAAAGTAGAAGAGGACCGAGGTCAAAAAGTTCACAATATCGGG
GTGTTACTTTTTATCGTCGCACTGGTCGTTGGGAATCTCATATCTGGTGTGTGTTTTGTGAATTTTTGAGCTTATTTTGG
TTAATTTTTGAGAAATTAATTGTTGTTTTTGTAATGTATTTTATGCAGGGATTGTGGAAAACAAGTTTATTTAGGTAAGG
GAAATGTGTTTATTAGTATACGAGTTTAAGTTTAATGCACCGTCGATGTAAAAGTTATTTATAAACATGATATTGGTGTA
TATAAGTGAAACTCGTAAATCTCTTGATAAGCATTACTGAAATTGATAATCTAGTAAAAGTTTTAAGTAATATGTTAAAT
ACAGGTTGAAATACGACGATAGTGTGAAAACATGAAAATCGATGTATTTGTTATATCAGTTTAGACGAGTTTGAGTTCAA
TTCACTGACACTGTAAAATTTATCTATAGAAAGGATTTACACTGACTTTATATATAAGTTAAACTCGTAAATCTCTTGAT
AGACATTGATTAGTAATCTAGTAAAAGTTGTAGCTATATGTTGTTACGGGTTAAAATTCGACGATACTGTAAAAACATCT
TTACATCGTCGGTGTATATAACTTAAATTTGATGATTTAAGATTTGTTTTGTTAATTTAATGATTTATGATTTGTTTCTA
ACTGTAAGCTTAATTTGGAATTTCGGACTTTGTTTTAATTACAGGGGGATTTGATACTGCACATGCTGCTGCTAGGTATG
AAATCAGTTAAAATGAGTTTTGTTAGTGAAGTAATTTTATAATTGGAGTAATTTATAAGGCAGATATAGGTCGTTATTAG
TACTAAATTTCGATACTAATATCAGGGCGTATGATCGTGCTGCGATTAAATTTAGAGGACTTGATGCAGATATTAATTTC
AATGTCAGTGATTATCAGGATGATCTAAAGCAGGTCTGTTGAATACTAGCTAATTTATTGAACTACCTAAGTTTGAAATA
TTGTTTTTTCTCGATTAGGAAACTGAATTTAGTTCATTTTTGTTGTTGTCTTGTGACTGGTATGTAGATGATGAACTTTA
CAAAGGAAGAGTTTGTGCATATACTTCGACGCCAAAGCACTGGCTTCTCCAGAGGAAGTTCGAAATACAGGGGAGTCACA
CTGCACAAATGTGGACGATGGGAAGCTCGGATGGGGCAGTTTCTTGGAAAGAAGTGAGTTGCCTGAGAACTTGTCTCACC
ATTGCTTAGGAATTGTGATGTTACCAGTAATATAGCTGTTTAAAAGCTTATTGGTATATGATTTTATCATAATACTGAGT
TTATAAGTTAAAGGGAATATTGTCTCATTGGATATTTGACTGCAGGTATATCTATCTTGGACTGTTTGACAGTGAGATAG
AAGCTGCAAGGTCCAATGATCATAACTTAACTCTCCCATCCATTTTATGTTATGTGGTTTATCCTTTCAACATTGACTGG
ACTTGGTCGTTTGTGCTTTCTCAGAGCGTATGACAAGGCTGCAATAAAATGCAATGGAAGGGAGGCAGTCACCAATTTTG
AGCTAAGTACGTATGAAGAGGAATTGAGTACTGAACCTGATAATGGAGGTGCTATGTTTAGGCTTTTGTACATATTTGGT
TTCCATTATGATAGTTGCTTGCGTGTTGAACGACCTATTTTTGCATCCTTGTGTAGGTGCAATCCATAATCTTGATCTGA
ACTTGGGAATAGCGTCCTCTTCTTTAGCTGATAATCAGCATGGCGACACCTGCCTAATTGGGCCTTCTGAATTCCAATGT
GCCTCAATTGGTTTGCCTGAATATCGTGGAGCCATGGTAACTTGGAATTCCATGATTTTCATTCACATTCGTTCAAGTCA
TCTTACTTGAAAATCTTTCTGCAAAGGAAGTTTGGTAGTTTGTATTTTCATAAATAAGCAAGCATTCTTAGTTGCACATT
TTTTTGCAGAACTCTCCTTGTACCACAATGGGAAGTAAACTGCCTCATGGCCGTCACCTGCTCTGGAATGGAGTAAATAC
CAGTGTCTTTCCCACATTTAAGGTATTCTTTGTCAAAATTTCTTGAACCGTTTTAAAGTTTTATCGAATTGTAACTGTAG
CGCTCACTGCCAAGTCTATGTCCCAGTTATATATGGTGCTTATTTTAGTTTAAAGTGTAATCAGAAACAAATAATGTTCC
TTAGCTAACAAGTTAGGAAGCTAATGGTATCTCTGTCTAACCTTGGTTCCTTATCTACTTAAATTACAGTCTTCAGTTAT
CATTCATAACTTTATCAAGATTTTTGCCAGCATATGTGTAGCATATATGTCTTAATATCCAGTTCTCTTGATCTGATAGC
TTTAAGTTGTCTTATGTAACTGCTTCTTATGGGCCTGAAAGTTCAAGAAGTTTGAGAATTTGATCTTCAGTTGAGCATTG
AATGACATATAACAATGCTCCTTCAGGGAACAGCAATAGAGAAGGGTATGGAAGTTGATTCCTCACCAAATTGGACGTGG
CAAGACCAAAATCTTTATGGTGGGAGTTCTTCAGTGCCACTCTTCTCTACTGCAGCATCATCAGGATTCGCTAATTCAAC
TACAGCTGCTGCTCATCAACCTTGCTTTTCTACTGGACCATTACCTTACCATCATTCACCATCACTTGCCAATATGAACT
TTGCACATTATTACTGCAGAAGCTGA

>*BW-BOB*_(reversed) 12199047
CTGATTCCACTTTTAAGTTTGGATAGCAACTCATAAATTTTTTATATGCAACTCTGAGCAATTTCTTTATGGACTTTGCA
GGGATGGTGGGAAACAAGTCTACTTGGGTAAGCAACTGTGTTAATAACCTCATGTTTTATTGATATGAAAGTTATTCCAG
CTTTATTCATGAATTGTTGCTGACTGCAAGCTTCATTTGGGCATTTGGAACCTGTATTTGCAGGGGGTTTTGACACTGCA

CATGCTGCTGCTAGGTACTGTATAATTTAAAATAGGTTTGACCTTAACGCCATGCTGATATTTTGTACTTTTCAAAAGAA
AGAATTGTAGTGCTAAGTTTTTTTTTTTTTTTGGGTACTAATTTCAGGGCATATGACCGTGCTGCGATTAAGTTCCGAGG
ATTTGATGCAGATATCAATTTTAACGTTAGTGATTATGAAGAAGATCTGAAGCAGGTTGGTTGAAGTAGCTAATACTGCC
ATTACTTCTGGTTGAAGTCCCTTTCCAATAAGGAAATGGCTGCTATGTAATGTAATTGTCTGACTGGTATGTAGATGAAG
AACTTTTCCAAAGAAGAGTTTCTGCACATACTTCGTCGTCAGAGCACTGGTTTCTCTAGAGGAAGTTCGAAGTTCAGGGG
AGTCACTCTGCATAAATGTGGACGATGGGAAGCGCGGATGGGCCAGCTTCTTGGAAAGAAGTGAGTGACTGCATGTTTCA
ATGTGGCTTAGAAATGTCACTCTATATTTAATGTGCCTATTTCAAGTTTTCCATTATCTATCACATGATAGCACTGATCT
TTATAAGCTAGAGGGAATATTGTCTCACCAGCTACCGTATGTTGTATGCCTGCAGGTATATCTATCTTGGACTATTTGAC
AGTGAGATAGAAGCTGCAAGGTACAATAATCAAAACCAGACTCTTCATTCCCTTTCATGTTATGTAGTTTAACCTCTCAA
CATTGACTAAACATGGTCTTTGCATTGGGGCTTGATCAGGGCATATGATAAGGCTGCTATAAAAAGCAATGGGAGAGAAG
CAGTCACCAATTTTGAGCTGAGCACATATGAAGGGGTATTAAGTTCTGAGACTGCTGATACTGGAGGTACTATCATGACT
TACACCTTTTTCTGGAAACCATTTTTATGTTTGTCTCGTTTACTCTTTAGCTTTGGTTAGCTGGTTGAAAACTACTAACC
TTTACAATGGATGCAGGCACAAGTCATAATCTTGACCTGAGATTGGGCATATCTCCCTCTTCCTGTGCTGACAATCAACA
TGGAAATACCAGCCAAATGGGAATCTCTCAGTGCCGGCCTGGCTCAAATGGTTTACCTGAACATAGAGAAGTCTTGGTAA
TTTGAGACCTCATGTTTCTATCTTCATCCATAGTCTTCCATGTATACTAGTGAGATTTGTAACAAAGGTAGATTTGTATT
CAACTTCTATGGCTAAGCAGCATACTTGTTGTGCCTCTCTTGCAGAGCTCTGCTTCTACTACACCAAGAAGTATGCTGCT
TCATGGTCAGCATATGCTAGATCAGCACCCCCTCCATTGGAATGGACCGAATGACAATCTCTTTCCCACATTTAAGGTAA
CTTCTACATTGCTAGGAATTAGGATTATTTAATTTCTGCTCGTAAAAAATGGTGTTTGTAGCCTTGATTACCTAGTTTCA
TCGACATGTTTCTTATTTATGCTTAATGTAATTTGGAAGCATCATTGCAGTTACCTCACAGGACAGGGAACTTATATAGT
CTCGGCCTAAACCCCACATTTCATGATTTCAAGAGCATCATCTTATTTATTATTGACAATCTCAATAAAATTTCATCTTG
TATATATGTAGTTTATTGAGGTCAGAAAAGATTCTCTTTTCCTTCATGGTAATTGTGACAAATTATCTAGTAGGAGAACC
TTTTAGTTCTATGTAAATGCCTTACAATGTGCATCTTTTAACAACATCCTATTGGCACTTCCTGCAGGGAACATCAATAG
AGAAGGGCTTGGAAGTTGACACCTCATCCAAATGGATGCGGCAAGACCAGAATTCTTATGGTGGAAGTCCTACAGCTCCA
TTCTTCTCTACTGCAGCATCATCAGGATTCGTAAATTCAGCAATCAGTGGACCTTCAGCTGTTGTTCATCAACTGCACTT
TCCTAGTAGAGCACTACCTTATCACTATTCACCATCCCTCACAAATGAAACCTTCACATTATTATTGCAGGAGCTGAAAT
AG

Table S2. Nucleotide sequence of B-class genes from 'Baccarat White'.
This table presents the nucleotide sequences of B-class genes identified in 'Baccarat White' (BW).

>*BW-DEF* 12708713
ATGGCTCGTGGAAAGATCCAGATCAAGAGAATAGAAAACCAAACAAACAGGCAAGTGACATATTCTAAGAGAAGAAATGG
ACTTTTCAAGAAGGCTAATGAACTCACTGTTCTTTGTGATGCCAAAGTTTCCATAATTATGATTTCCAGTACTGGCAAGC
TTCATGAATTCATTAGTCCATCTATCACGTACGTATCCAAGAACTAGTATTCTCTTGTGTATTTCAAATTTGTATTTATG
TTAATTATTTTGTGTTTTTGATATTGTTTGTATGTTCATAGGACTAAGCAGTTGTTCGATCTGTACCAAAAGACTGTTG
GAGTTGATCTTTGGAACTCCCACTATGAGGTTTTGTGTCGCTTCTAAAGTTTCTAGATTTCCAACTCGATATTTTGTTAA
ATTGTGTGTTAATTCCGTCTTAAACTTCGGTTCAAGTCAAACTAAGACAATCATTTTAGAATCGAGGGAGTATATTTCAG
TGTTTCAGATTAATTATTGTTACTAATATTGCGCAGAAAATGCAAGAGCAACTGAGGAAGCTAAAGGAAGTAAATAGGAA
TCTCCGAAAAGAGATCAGGTAGGTACAAACCTAGGAGTTATAGATAGTATTTACTAGGAATATACTTTATGATATTTTAT
TGATATGTCAAAATAAATGAACAGTATTTTTTTTTAAAATAAAAATTTCTCTTTAAGTTTTGCTGATATTAACAATGGAC
AGGCAGAGGATGGGAGAAAGCCTAAACGATCTGAACTATGAGCAGTTGGAAGAGCTCATGGAAAATGTCGACAATTCTCT
CAAGCTTATTCGTGAAAGAAAGGTTTAATATATTTGCTTTTCAAATTCAGCAATTTGCCAATACCTACTAAATTATCAAA
CTAATTTTTTTAAATTGAATCAACTGCAGTTTGCTTTCTTTTCCTTTGGATCTACTTGAATATTCATGTAGATCAACAAA
AAGAAGAGATATAAAGCAACAAAAGAGCCTAGAAACAATCGGAGTAGCTTACATTCTACTTTGTTAATTACTTTAAATTG
CAAATGGGATACTAGGAAATGTCTTTTCATAAAACTAGACAGGGAAATGTTTGCTATTCCTTTATTTAACTTCAGCATTC
TTCAGATGATATGACAATCCCACCTAATTATAGGTCAAAATTACTCTATTTTCTTGGTTTCCCTCATCCCCTCTCAATTT
CATTTGATAATAGAGTGGCTGGCATATAATTATGATAATTTTACTTTTAAATGCATTTTGGAGTTCACATATGTTGCAAC
TATCAGATCGATATTCAATCATTCGGCTAATTTAGATTCGTTTTGACTATGGTTAGAGTGTTTCATATTAAGAAGTTCTT
TAATCTAGAGCTGATCAAACTCGAAATATTTAGTTAGAGATGAAGGAATTCCAACATTTCACTATACCACTTGTAGTATC
ATAGACTTTATTTCACTAGCACCAAAAATTCCACTCCCATACATCGATCCATCCTTAGACTATCAAAGATTAAGCAAGCA
AATGAAATAAAAGAATAATTCTATTTCCTTGTTTCTTTCTGTCTTTTTGGAAGATTTATCGAATAGGAGAATTGAATATT
CTTGATATATTTTCATGTCCTTAGCTTGTCTTCCAAAGTTACTCATTTAATTTATGTATTTTGTAAACAATGCCTAACTT
AGTTTCATTTGTGAAAGAACAGTATAAGGTGATTGGCAATCAGATTGAGACATTCAAGAAGAAGGTAAAAACTACTATCA
GTTGGCAGTTTTTGTAGGAGTTACTTCTTGAGGGTGAATTCTTATTTTGGTTGATTGTATATTTGCAGGTCAGGAATGTG
GAAGAAATTCATAGGAATCTCTTGCTTGAATTTGTAAGTGCTTAAACTTATTAATTCCAGCTAACTCACTGTTTTGTTAG
AAGCAAAAGCATCTGCCATTGGATTCATATATATTGTCAAAGGCTAAATAGATCTAGGTGAGGGTTGACCTGTTAATTGA
TTTTTTCTTTATGTGAGAATGATGAAAATGATGTTGTGTACGTTGAGAATGGTTCTGCCCCTAAGATCGTTATAAATTCA
AGTAAGAGCCTTAAATTTCCATAGTTGGAATAATTCTTGAATCAGATAAAATAGAAAAGAAGAATAAAGCAGAAAAAATA
TGTAAGATAAGATAATCAACTACTAAAAACTACTCCATAAGATAGTTCAATTGCTAGGATTATTTTTTATAAGGAGCCCT
TTGTCTTCATAGTTTATCACTAATCCTGGACTCAAATTAATGGGATCTAACAGCCTCGAATACCAAAGTCCTAAAGTAA
AAAAAAAAAAAAGTTTCGTTTATGTACTCTTAGTAAAAGAGTAAGAAATTTAAGCAAAAAAATTTATAAGGTAGTGCATA
TGGTGGATCTTGGATTTTAAATTTATCGTTACGGACTATAACCCTTCTTGCTTATTGGGCTCTAGATAGATTATTTATTT
TTAGAGTATTTTTTATAAACATAAATACAAAATTTAGACCAAAGTTACTGAACCATTAGTAGACTCGTAGATCTGCGCTT
GATAAGGAATTGATGATTTTGGATATTTGTGCAGGATGCAAGACAAGAGGACCCATATGGGCTAGTTGAACAAGAAGGTG
ACTACAATTCTGTGCTTGGTTTTCCAAATGGAGGGCATCGCATATTAGCCTTACGCCTTCAACCAAACCACCACCAGCCA
AATCATCATCATCATCTTCACAGTGGTGGAGGCTCTGATATCACTACTTTTGCTCTGCTTGAGTAG

>*BW-GLO1*
ATGGGGAGAGGAAAGATAGAGATAAAAAGAATAGAAAACTCAAGCAACAGACAAGTAACTTACTCAAAAAGAAGAAATGG
GATCTTGAAAAAAGCTAAGGAAATTAGTGTTCTTTGTGATGCTCGTGTTTCTGTTATCATTTTTGCTAGCTCTGGCAAGA
TGCATGAGTTCTCTTCTACTTCGTTGGTTGATATTTTGGATCAATATCACAAGCTTACTGGTAGAAGATTGTGGGATGCT
AAGCATGAGAACTTGGACAATGAAATCAACAAAGTCAAGAAAGACAATGACAACATGCAAATTGAACTCAGGCACTTGAA
GGGTGAAGATATCACATCTTTGAACCATAGAGAGCTCATGATATTGGAAGATGCCCTTGAAAATGGACTCACTAGTATTC
GTAACAAACAGAATGAGGTTCTGAGGATGATGAGGAAAAAGACTCAAAGTATGGAGGAGGAGCAAGACCAACTTAATTGC
CAATTGCGCCAACTTGAGATAGCAACCATGAATAGGAATATGGGAGAAATTGGCGAAGTGTTTCAGCAGAGGGAGAATCA
TGACTACCAAAACCATATGCCTTTTGCCTTCCGAGTACAACCAATGCAGCCAAATTTGCAGGAGAGGTTGTAA

>*BW-GLO2*_(reversed) 12691906
ATATGAAAATATTACGGTCAAAGTTAAACTCGTTTGACTCTCGAAATCCGAACTATGACAACCATTTTGGAACTGAGGGA
GTATTAATTAATTTATAATCAAGTTTATGTAGGAAATATGTCTTTTCAGGCACCTCAAAGGAGAAGATATCAATTCTTTG
AACCACAAAGAGCTTATGGTTTTGGAAGAAGGCTTAACAAATGGACTTTCTAGTATCAGTGCCAAGCAGGTAATTAAATT
TTCCCCTAAATTTTTATCAGTAATCATGTCTTGCCTTTTAATTACAATTATCAGAAACAGACTCTGATGACCTTTCTTTT
TCTTTTTGTTTCTAATTACTTACTGTTCTACAGTCGGAGATCTTGAGGATGGTCAGGAAAAATGTAAGTGTTTGCAAATA
AAGATACCTGCTTATACATAGGTCTGAATTAATGAATTAATGCATTTGGGCCAAGTACTAATATAGTATTTATTTATTTA
TTTGTGATGAATAATTCAGGATCAAATTCTGGAGGAGGAACACAAGCAACTTCAATATGCTTTGGTAAGTCAATCATACA
TGGTAAAATTTACCTACAGCCGATATTTATTTCACCACCCTTATCCAAAACAATTATTACACAAAATCATGTAAGTTTCA
CCTCAACAACTAAAATTAAAGTGATTATAATTATCATTAAAATTTGGTTAAGTGACAAATTGGGTGTATGCAGCATACAT
GTCTAGCATTCTTTGATTGGCGTAAATAACTGCGAGTTTTCCAACATATTTTTAGTTACGGCTCTGATTTTGATAGTAGT
GATTTTTATGTTCATATTGGCTCTGGGTTGGTTATTCTTATTGTAAATTGGTAAAAATTTCAAGTTTTTATGGGTAAAAA
TCTTAAAATTTTGTTTATTGGAGAATAACTTGACTTATATAATCAACTTTATTAATCTGTGCACAGCACCAAAAGGAGAT
GGCAGCCATGGGTGGAAATCTGAGAATGATTGAAGAAGTGTACCATCAAAGAGACAGGGATTACGAATACCAGCAGATGC
CATTTGCCCTTCGAGTTCAGCCAATGCAGCCAAATCTACATGAAAGAATGTAGAGCCTATAATTCTACTATATGCATTTT
AAATGAAAGATCGTTAACAATTTAGGGTATGTACTAGAAGACTTCTAACTAGTGATATGGTGAATTAACTAGACTATTGC

GGGACAACCTTTTCATGTGTGACAATATAATTATAGTATATTACCTAATGGTTGTACTGAATATTGAAGTTCTCTTCACT
TCCTACATGGTTTTAAGTATTGATATGCAATGTTTGTGCTACTATATATAGATATGGCATATTTTGGTTGCATTGTTATT
ACGC

>*BW-TM6*
ATGGGACGTGGAAAAATTGAGATCAAGAAAATAGAGAACTCAACAAACAGGCAAGTGACTTACTCCAAGCGAAGAAATGG
TTTATTCAAGAAAGCTAAAGAACTTACTGTTCTTTGTGATGCTAAGATCTGTCTCATCATGCTCTCCAGTACTAGGAAAT
TCCATGAGTATACCAGTCCCAACACTACGTACGTATATCATGTCTTTTTGTTGTTACATTAATCTTAGTATTAAAAACCT
TATTAAGAACATGATTTTGTCCTTCTCTTTTCCTATTTAAAGTTTGAATAAGTATATAGTTTCTGCCTAGCTTCGTCTTG
CCCTTGAGGGGATTTAAAACAGGATTTGTGGATTCAATTGAACATAGTTTCGATTCGAACAATGTATACATAATAAAACA
GGACATTTAAAAATATATCCAAATAATAAAGTTACATTTCTCTTGTATACTCACCTAACCTATAGCTAGATCTTACATTT
GACATTTGTGTCTGTCTTGACCAAACATAAGTGTATATATACAAAATGTAACTTTTTAAGAAAAAGACTATGTTGGAGTA
CTTGTTTTTTTGCTTTCTTTCTAACTTCTTCATTGTGTTAATCAGGACAAAAAAGATGATTGATTTGTACCAGAGGACAC
TTGGGGTTGATATTTGGAACAAGCATTACGAGGTAGTAGTTTTCTCCACTACTATTCCGCAATTTGAATAATTTGCTACT
TTGGAATGAACTGACATGTATTGTTACGAGTTATATACGTATGTGTGTATGAATTTTGCAGAAAATGCAGGAAAACTTGA
ACAGATTGAAAGATATCAATAACAAGCTAAGAAGAGAGATAAGGTAAGGCATATGGTAATAGTAGGATATATCCTTGACA
CTGTTGAAGTGATTTTGGTTAGCAGGTTTATAGGTTGTCTTTTTGAGCTATTCAAGAATGATTTATGGGTTGAATCAATT
TTCGAACTCTTTCTATTTTGAACTAGGATTTATGTTATATAGACGGATAGTGCAACGTTTTTCTATACTATATGTGCATT
TAACATGCGATAGCCAGTTAGTTACAACTTACAAGTATCATTTTTACCAAATTCGAGATTTACGAGTAATTAATGAGTAT
AACTAGTATTTGATTGTGCAATGTATAGAATCTAAATTCCTTTCATAATGAAATGGCTGATACTCATGTTTGTCCATAGG
CAAAGAACAGGGGAAGACATGAGCGGCCTCAATTTGCAGGAATTGTGCCACTTGCAGGGGAACGTCTCTGATTCTCTAGC
TGAAATACGTGAAAGAAAGGTTATGATTAATACCTTGAAGCATTTTTATCTGTTATTTCCCTCTTTCATGCCTCTGTTAC
AACAATTTCTTGATCTTTCTATTTATCGAGAAAAAAAATTGATCAATTTTTCAACCAGGAAATTTTGTTTCTCCTAAACAA
GTTTAAATTTATCGGTAGCTTAAAAATATTCACAGTAAGACTAAAGATTATGAATAACCTATTGAGAGTCGGTCAAATTA
CACTGATTGTGAGTTTTTTAACATTGTTCGTTTGCAAAAGTAAATTCTCTTTGCATTAGTATTATTTACCTACTTAACTT
TAAGTGCCTTTATGAATTACATACAGTATCACGTGATCAAGACTCAAACAGATACCTGCAGGAAGAGGGTTAGTCCTCCT
TTTACTACTCGTTAATCTTTAATTAATTCCTTTCTTTTAATTCATATACAAGGTGCAATATTTTGTTCTTGTATGTGTTA
TTTACAATAGGTGAGGAACTTAGAAGAGCAACATGGAAGCCTCGTACATGATTTGGTAAGTATATTTTACAGAGATATAA
TACTTTAAGTTTATTAGTAGATTGTGTGGGATTATTTAGTAACACAAAAATTTACTGTAATTGACTTATACAAGGAAGCA
AAAAGTGAAGATCCAACGTATGGTGTAGTTGAAAATGAGGGACATTTCAACTCTGCTATGGCATTTGCCAATGGGGTACA
CAACCTTTATGCTTTTCGCCTACAGACATTGCACCCCAATCTTCAAAACGGAGGAGGTTTTGGTTCTCGTGATCTACGTC
TTGCTTGA

Table S3. Nucleotide sequence of C-class genes from 'Baccarat White'.
This table presents the nucleotide sequences of C-class genes identified in 'Baccarat White' (BW).

>*BW-FBP6*_(reversed) 12609230
TGGGAAAATGTATAATAATACAACAGACCATAAAAAAGAAATAGGATCATACGAGCAAGTTATTTTTTTTGATGATCCAT
GTAAATCAATTATGACAAGCAGGGTAGTGGAAATATAGAGTTGGAAGTTTTAATTTCTAATTTATGATGTTTAAAGATGC
CACACCTCTTGCAGGAGATTGAGATGCAGAATGCCAACATGTACCTTCGAGCAAAGGTCCTATTCTTTTTGTTTACATTT
TTTGTATGAACTTTTTATACTCCATTTTTATGATGCTACTACCATATAGGGAATTAGCTAGGTTGTCTAGCATTTTCTGT
AATCTTAAAAAGTTTTGACTTGCAGGATTTAATAAAAAGTAGTAGTAATACTAATTTTATTTCATGGATATTCTTTCACA
TAGAAACTGAGATATTTCCGATGAATTTTTTGAAGCCAAGTGTATTACTTTGAATAATGTGTGCAGATTGCTGAGGTAGA
GAGAGCAACACAGCAAATGAACTTGATGCCTGGAGGAGGATCTGAATACCAGCAACAGCCCATGAGTAGTACTTCTCAGC
CTTATGATGCTCGCAACTTCCTGCCTGTAAATCTGCTCGAACCTAATCCTCATTACTCTCGTCAGGACCAAACTGCTCTA
CAGCTTGTGTAAGCTTCCCTTCTAACCTGCTTATTGCCACTGCTGGTTCCAAGATGGACATCAGCTTTAAATTTCTGACA
GTACGAAAAATAGCCTCCTAGCTGTTATCATCGATGGTTATACCAAATCTTGTATGTAAAAATAAGGAGTAGAATGTGAT
ATCATATACATTTATAATTATCTATTGTCAATTGTTGAGCTATATAGTTGTTCTATATTGATTAAGGGGGCTCCAAAGGA
GTTTCTAGAGGTCCTCAGCACATTCTTAAGGTTTAGGTTGGATGCCAACAATAGTAATCAAAAATCAACCATTGGTAACT
CCATCTTACAGTGATTAGAGCACACAAAGATTCCTTGTATGATTTGAACTCCAAAATTGGGTTACATATGTGGGCGGTGT
TGAATTATATCTGAATGGCTTCTCAATGAGTGATGCAGACCTAAGGCCACGGTGTGAAGTTTGCTAAAGTTTACATCGGA
TTAAGAAATTGTATCTCATATATAGCCAACCAACTCATCTGCCACACTACTTCCTTGAAACAATGTGGGAGTTGAGCCTT
CAATGCAAGATCATTGTATATTTTCTACATTTTATTGGATATGACAAGATATTTGTAATACTCTCTTTTGGATTATCCAG
AATAGTAGTTAAATGTCTCAACTATTAGGTCCATGATCCTTATCTTTTCTACAGATTCATAGAGCATTGCTTTTGCTTTT
GCTATCAAACTATTTCCTCTTCCTAGTGGTGTAGCATCTTGGTTAAAATTACATAATTTTGATTATTGCTTTTGCCTTGA
ACTGCATATTACTAAATACAACAATAGTTTCTAATTGGAACAGTTATGATGTGATCATTTACTATATTTAGTCTCTTACT
GATTACTTTCCCTTATTGCTGCCTGATGAAATCTATGCAGCTGATGATCTTGGAGGAATCTACTATCCAGTTTTCTGAAT
TTAGCTCAGGGGTCTTCCTTCATCTTCAGAAACGTCCTTCAGTTTTAACCTATAGTTTGTAAATCCTCAGAACTGCTACC
ACTGAGATTTGTATGTTTCTGGTTAAGCATTTAGTTATCTGTTTCGTTAAGGA

> *BW-pMADS3*
TAATTAGAAAAAAAAAAAAAATTGAGAAGATCACTTTCCTCAATTCTCAAAAATCTTTGCAATTTTCACGTAGTCTCAAC
TTTTTCAAAATGACACTTAGCCCCTTAAGATTTTGAGAAATGGACAAGATTGTATTAGTCCCTATCATAAGACACCTCAT
GTTTATATAGCAGCGAAGCAAATTTTATAACCTATTAAATTAATGGTGGGCGAAAATCCGTTTGCAAGCATTAAAGAGTT
TTTATTGAATGACGACCCGCATTCTGGCTTCCTTTATTTCAAAGTGGAAGTTGTACCTAGTGTACACTGGTTAGAAGAAG
TGGACATGACACATCAAAAAGTTACGGCACACCTTTGTAAATAACATTCATTTCTAACCATATCTTCTCTGTGACTTATA
CCCATATTGTAGTTGCTTCTAGGGTTCCACCGGTGTATTCTTAACTAAGCACAAAAAATAAAATAAAATCTTCCTAGGCC
CAAGTTATGTTAGCCTTAGCAGTTAATATGTGTACTCCTTTAAGGGAATAGACATTTTCAAATATACGCTTCATAGTCTT
TTCCCCTTATACGTGAATCTTGTGACTTCAGTGGTCAAGGGAAGCTAATGTATTTTCTTGCCAAACAATGTTATGTCATT
GCAATGTTAAACATGTCTCATGGTTGGCATAGAAGAATGACAATTCCTATGTGAAAATAAGCATTAATAAGAGCAAAAGA
ACAAGAATTTAGGCTAATGTCTAGAGAGGACTAAACTTATTGTAACGTCGAATCAGATCAAAAGGGTAAGGTCAAAGGCT
AAGTAGTGGCAAATTGAGATGAGAGTGAAATTGGATGAAGAAATCATGATAACCTAACAAAGTAGACAAGAAAATGCAAA
AGGTGGAAAAGAGGAAATTGAATTTGTCGTATATGTCATTGAGCACAACCAGCCATATAGACAAATTTATGTCCTTCTTC
CCAAAGAAACAAGAAACTTTTTGGCATGATGGTAACTTGATAGATAAGTGGTTGTTCATAGATGAGTTTCAATAGAAAAG
AAAGTAGAGCACTGGATGTCACATGGCAGTCACCTTCTCTGTTGTCATTGCAAGTTTCACTGATCTAGTTCTTTTCTCTT
TCTTTTTCCCTTCAGTGATCTATTTATAGTCTAAGTTTAATGTGAGAGATAAAACAAAAGATCACAAAGTAAAAATTATG
TACACATAGGGTTGGTAAAGTTAAGAGAAGAAAAGTAGTGATGGTGGGGGAGAATAGGATGTTAAAAGCAAGGATTCCAA
GTTAGTTTTGGGGGGAGGGGAGTAATGGGATGTGAAGTTCACATGTAGAAATAAAAAAATTAAAATATTTTTAGGAAAAT
ATTCAAATGGTCCAAATCTTAATAGGGCTTAGAAGAACTTTAAAAGCTCTAATCCCATTTTTGAGAACATTTAAGCTTGA
GTGTAACCTAGTCCAATGTTTCTCTTCACCCATCAAACATCAGAGTCACTTTCAGGGGTCATTTTCTCAACTCTGTCCAC
TTCCTCCCACCACCCACCCCACTCCACCCCCCTCTCTTCCAATAAATGATTACAATCTAACAAGAAATTAGAATATAAAC
AAGAGATCAAGAAATATGAATGGCCAAGAAAGGCTGATTATCATGAGATAATTCCCTAATTAACCATCTATCCTACTTGC
TCAATACAAGCCAACAATCTGAAAAAGAGAACTATTAAAGAAATATGTCAAAGTTTAATGTAAATTAGCATAAGAATAAA
CAGACAAAGAAAATTCAAGTTAGTTAATAGTAACATAATAAATTAAAAGAAACACTCTTTACTTTATAAATACCTATCCC
TTAGTGCAAACTCTCTTCCATTTCTGCATCTATCCTCTGCAGATTAATTTGCAAAGGAAGAACTAAAAGCTTCTATCTC
TTATTCCATCTCCAAATCTTCTTTTCTTATCAGGTTAGCAATTAAACTAAAAACATTTACACACCATGTCAAAAAAATCA
ATTTGAACCATTTTTTGAATGTAACATAAAATAAGTGGTTGGATATTGATCAATGCAAGATAGTTTGAGTTAGTATGATT
AGGAAAGTATTCACCAAATTCTTTAGCAATCATCACTGATTGCTTCATTTTAGTTGTATTGAACAATAAAAGGTCATTTT
TTCCAAGTTTGTCACATAGTTTTTTTTTTTGTTTTTTTGTGTTTGATATAGGTGCTGCAATGGAGTTCCAAAGTGATCTA
ACAAGAGAGATCTCTCCACAAAGGAAACTAGGAAGAGGAAAGATTGAGATCAAGAGGATCGAAAACACGACAAATCGGCA
AGTCACTTTTTGCAAGAGACGCAATGGTTTGCTCAAAAAAGCCTATGAATTATCTGTGCTCTGTGATGCTGAAGTTGCTT
TGATTGTCTTCTCTAGCCGAGGCAGGCTCTATGAGTATGCCAACAACAGGTAATCTTTTAACAAAAAAAATTAGAAAGTG
TTAATTTCAGAAAGTTTAATCTTTACCTTCTTGGGTCTCTACAGCTTTGTCTACCTAGCTTTACCTTTCTTCTTCATTTC
TCACTTCTTCTTTTCTTGCAATTCTGTTTGTCTTCCTTAAAAGAGAAAATAATCGTGATTGGAAAATTCGCTTGTGTTAC
AGAATGCTTAGGAAGATCTGAACTTTAAAAGGAGAAAGATCATGTTGTTCTGAAGTTTTTAAACTAAGAGTTTTTCCTTC
TAACAAAGAATTGACTTTCCTTTCCTTTCTTTTTTTATTGTTTAACAAGTTTTGTTTGTTTCTATTTTTGTGATCCCATT
TTTGTTAAAAGACTTGTAGATGTATAGATCTGTTTGTTGTAAAAATTTGGGAAGGTACTCATCACAACTCTGAGATCAGC
ATAGTTTCTTCATTTCAGTTTCTGTTATGTTTTCTCTCTCTCAAAGATGAAGCAAGAGAGCTGTCGAGTTCTAATTCGCC
CATTTAGCCCATCATTAAATTAGAGTTAATATGGGATTTAGATCTTATAGTAGTCCTTTCACAGCCAATGAAAATTCAGA

TTAGGGTTTTGTACTTTGTTAATAGCTGTGCATTACTGTTTTTGTCTTGAACTTTGTTTCTATTGGCAGAGTCAAACTTG
CCCTAATTAGGGTTTTTTTTTTTCAGCTAGAAAATTATTGGTGTTTTTCCAATTCTCTAAATTATTTCTTCTTTAACAAT
AAAATCTCAGATATGCTTCTCATGTGTAGTGCAAATTCTAGTGTTTTTTCTTGAAATAAAATGTAGAAATCCAATCAGTG
TTCAAAGATCAATTATAGACAAAAGTTGACATTCTTCAAAGTGTTTCCGTTTTCCCTTTTTCTTTACTTTCCCTTCCTAT
TTCTTAAAATGTTTTACACCGTTGAGGAAATGGGGTCATCTATACACTCTACTCTAGCTTAGAGTTTTAAGAACCAGAAA
TTTGTTGGGGTGGGTGGTTGGTGTAAGTTTTGTCTCAGTTACAAGAATCTCTGGTCACGTCATACAAGATTTTACACTTG
TTGACAAATTTGAGAAAAGACATATTGTGATGAGGGAATGGTTTCCACTGAGCTATCTCTTAAATTTCTATCTTCAATTG
TGTAAAATTGAAAACACTAGCTCCTTATTTTTCTTTGAGATATCTCAGTTTAATGCTTTCACAAAGTTTCTGAAAGTGTT
GGAACTTAAACAGCAAAGAAACAGAAGTGGAAGAAAGAGAAGTACTAAACCAATGAAAATTTTACTCACTTAGCTAAGTT
TTCAATAGGATCTTGTGCATAGATATATTTGCGTAAGACATGTAATAGAGTTCGGGGGGGNNNNNNNNNNNNNNNNGGGT
GGGTGTTGAGAAAGAGATCAGACATAGATCCGTACAATTTATATTCCAACCAATGAGAGAGTTCTGAACTCTCTGGCTTT
CTATCTTGCATGGTATTGGCTGCTGAAAATGGACGGTTGTGATGTGATCAGATGAGACAGACAAGACTGGATCATACTAT
TCACTTTACCTTACAACACTGTAAAAACATTATGTTTATTATAGATTATAGATAAAATGCCTTTGAAACAAAACCCTAGG
TGGGTAAAAACCTAGAGTTGATGGGTATTACCCAAGGAATAACTGGTACATGATGCGTGGCATAGATCTGAACCGTTGCA
ACAGTGGACCAATCATGTTGTGGGAACTTGTGCCAGCTTGGCAGAGAGCAGCCAATCAGTAGCTCGACGAAATTAAGGTT
GTAAGTAGACTAGGAAATGAGAGTCATTCCTCAGTGTTGGTTCTTTTCTTTCAATCTGAAGGTCTATAGACTATACTAGA
AAATTTTGTATCAATGTCATGTTTGAGTAGACAGATTGAGTCTGACTCAAAGCAACACTTTGTTTTTTATTTAAGCTTTA
CGTGTTGGCCTAATTTAGCAAGACATAATAGGTGTATTAAATATGGAGTAAGAAAGTATCTGGATTTTCATTACCTTTTG
AACAAAAGGGTAGTGGTTAAGGCACTCTTTCTATGTTTGCTTCAAGTGAACAAGCACTCGTGTCTAAAGATATATGAATA
GCTGAAACCTCTCTCCTTATGTTTTGGTCCGTAGTATAAGCTTTGTTTCACGCTAAATTAATTTCTCACGAACTTAGGTA
CAACACTTTTTGGGGGAAAATGACACTCTGTTTTTAAAGTGTAGTTGTAATAGGAAACGAGAAAATTTTAAGCTTAAGAG
AAGATTAAATTTGAAACTGATATTGTCATGAGCAATTTGACCAGTGTGGGCAAATAATTAATACTCTTATCTATTCTATT
TTATGTAGGGACGTTTCTTTTTTGTGAATTTTCAAAATAAATAAATAACTTTCTAAATTAAAAAATAATTTAAATTAAAC
TTCTATATTAGTAGCACAAATTTGTTGCCAATACTAATCTCACTTTTTTTATTTTGGTAATATTTGTAGTACCCCTTTCC
CCAATATATATTCTTTTAAATGTTTAACATCACAAGTTTCAAAAAAATTTTGATTGATAGGTTCCCAACACTCTTCACTT
CTGCTTTTAATTCGGTGCTTATCAATACTTTGTCAAATAAATTACGACAAAGGTAATTATACCTGTTGGCTGATTTACTA
TCTTTGTGTGAAAATTAGTGAAGTTCAATACTAGTAAATAAAGATCTGGGCAAGCTTGCGTTACACATACTTATCATCAC
ATCTTATACAGATCCTTTTTCTTGCCTAAATATTGGAGAGTACAATTAATTATCTTTCTTCAATTTCCCATCTTTACTTT
GTCAAGCCAAACAAACGACTGCAGTTTTTTTCTCTTGTTTTCTTGTATTTTCCCCAGTATCTCAGCGAAGGGTAAATCTT
TCATATTGATTTATGCAAATGTGTCATTTTCCTTTTCCTTTTTCTCCTTTCTTTTCAGCCTTTACACCATTAAGGAAGAC
TTAGCAGGTGTTAAGAACACATAGCTAACTACAACTCTACTGTGGGAAATGTGTTTTCACAAGTTGCTTGTTGACAGCCA
AATTGAAGGGAAATGCTCTTTCTTTTGTTATGTAGAATAGCTAAGCACTAGAAACTTATAACCTACTAGCAAATGATTTT
GTGCAAGCAAATCTTCTTTAACATTATTAATTTTCTTCCTTAATCTTTTAAGTACCCTGAAAGCTCTAGATTTGCTCGTC
TTTCATGTTTCCTAGGGGAGTTGCAAGATGTACGTGGTCATAAGTAATAGCCAAAAATCAAGAATACTTTAGCAAATGGA
ACAAACCGTGGAGATGGCTTTGGAAGATGTTCCTTTTTGTGTTCTTAATTTTTTTTTTTTTTTTTTTTTGKGGTGGAGGT
GGGGGTGGTGGGGGCTTAAAATGGAACTTAAATTAATATTTAAATATTTACAAGAGTACAGCGGAAATAGTTAATGCATC
TATTTCCTCTGTTTAAAAAAAGATGAACCTATTTTTTTTAGTTCGTTTAGATAAAATGATCTCTTTCTAAATTTTGAAAT
AAACTTTTACTTTTACCTTTAATGAGATGATATAGCCTACAAATATCTATGACTTACTTTAGACCAAGTTTCAAAAGTCA
TTTCTTCTTTATTAAACTCCGCACAAAGTTAAATGGATTCATCATTTTTGAAACAAAGAGAGTAGTGCCATGAGACGCTC
CTTTTCAAAAACATCAGTAACAAAAGGTTGCGGACTTGCGGTGTGCAAACACTAATTTTGTTGTAAAAAGGGTAGTTTTC
GTGGTAAGAAAATTTGCCTCATTTTGCTGACACCATAGGAGGTCACACAAGAGGGAGGCAAACCTTATTTTGGAGGAAGG
GTCAATAGATAGAATGCTATTAGGAAACTTTTAATTCGATCTTGATTTTGATACCGATCAAAAATATATTAATCTGGTTG
TAACGCGATAGCTGATTATGGTTGTCTATAAATTTTAAGATTGATTAGAAACTGTCTAACATTTTTATGGTCTAGTGTGA
AAGCAACAATTGAGAGGTACAAGAAAGCTTGTTCAGATTCCTCAAACACTGGTTCAATTGCCGAAGCTAATGCTCAGGTA
TAATTTCATAAAGTCCTCTTCCTACAACGAAGCCTATATTATGACTTGCTTTGATTATTCAAACTTCTTTGAGATTATCG
AACCATTTCTTATGTTATGTATTTTGATTCCCCTCCCCCTTCAGTAATGAAATGTTTTATTATATTAGACTTTATAGAGA
TAAAATCTACTACTACCTCATTTCAAAAAAAGTAACACATTTCCTTATTAATCTGTTGCAAAAAGAATAACTTGAACTTT
CTATTTTAACACAAATGTTATGATATATATAAAACCAAAAGTTTCAAAAGTTTTATAGTAACATAAATGTTATGGCACCG
GACCTCTATTGTTATTCACTCTACTTAATCAGCTGTTCTAAAGTCGTCAAGGTGGCACGATTGGATTAAGTTTTTCATCT
AGTATTTTATTTTGAAAGAATTAGTTTTGTTTTATTTTGGTCGTTTCTTGAAAGAAGGCATTTGTTTTGTTCTTTCTTTC
TTTTGGTTCTGAACTACTTATGTTAAACCATGTTGATTTGGTTTAGTAAACAACCAAGTTAAACTGAAATACATCTAAGG
TCAAATTCTAAGGTCATTATAGGAGCATATTCGTATCTCTAGATTCAGTTTACATTTAGTTAATTAAAGGCCTTCTGTCC
ATACATAAAGTTCTTAACCAATATGGATTATAAGTTGCAAGCATTATGGATTCTTGATCTCATTAAATCTTATGCTGTTT
AATTAAACCCAGTATTACCAGCAAGAAGCCTCCAAACTCCGTGCACAAATTGGAAATCTGCAGAACCAGAACAGGTGAAA
TTTATTGTTTTCTTTTTTTTTTCTCTTTACTTTTCTATTCACATTTGTTTTCTTACCATTTTTTTCTCATTTTGTTATTT
GTTGTTAATGGTCCTCCTTAACTCAACTACTTCGATTCAGGAACTTTCTTGGTGAATCTCTTGCTGCACTGAATCTCAGA
GATCTGAGGAACCTGGAACAAAAAATTGAAAAAGGCATTAGCAAAATCCGAGCCAAAAAGGTGTACTTACATTATTTCCC
AAAATTTCATATCACTTTTGTTTGGTGAAATTTTCAACTCCTTGTGATGGCTATGATTTACTAACATACCATTCTCAAAT
TACAGAATGAGCTGTTGTTTGCTGAAATTGAGTATATGCAAAAGAGGGTAAGTAATCGTGCTTACATACCATGAAAGAAT
AGTTTCCTAAAGTTTTAAACAGATGTTGAACTTATGTGAAATTCATAAAGATCAAAATGTTCAAGCCTTTTATGTTCATG
CCAAAAAGTAACATATGTTGATGCACAGCAGGAAATTGATTTACACAACAACAATCAGTATTTAAGAGCAAAGGTCCATT
TCTTACTCAAATATTTGCTTGTTAGTCCTTAAATATATTCCTCCTTCTGCAAAAATTATTGGTTTTTGTATCTAAATTAA
ACAGATTGCTGAAACTGAGAGATCCCAGCAGATGAACTTGATGCCTGGGAGTTCTAGCTATGACCTTGTGCCTCCCCAGC
AGTCATTCGATGCGCGGAACTATCTACAAGTGAATGGCTTGCAGACCAACAACCATTACCCTAGACAAGACCAACCACCT
CTTCAACTAGTGTGAGTACTCCCTCCTACTTAAGTTGCCCAATTATATTATCATTGTTTATTAATAGTATCATTCAAGTT
CTTGCATTGAGCATTCAGAATTGGTATACTGGATCAAATACTAGATATAATTTTTCATTAAAATAGTCATTTCAGCCACT
CCCTCGTCCCAAATTCTTTTGCACTCTTTTTGTTCAAGACTGGGAGAGTATTAACAAAACTGTTCAAAATAACTCTTTTA

TTAAATTGAAATGAATATTTTATTATCTCATAGCGGAAAAAAAAAGATCTTGAACTTGCAATACTTTTGTTCTACTTCTAA
ATATCTAAATTAGGCATTGTCTATATCGAACTTAATTTTGAAAATTGACCAAAAGTAGAGACTTGAAAAGCACAAAGAGA
AAGAAAAAAAGGAAGAAGTGAAAATTAGGACAGATGTATATCATCTAGGTGAGGGGATAGAATGCAACACTCAAAGGTACA
TTGGAACATTGATATTTATCTAGTTTTACTTAAGGGAAAACCCTTTAGTTTAGGAAATATAGGAATAAATTAAATACTTA
ATATGCATCATTAGATCATCACAAAACGTATTGATACCCCAATCCCCATACTAGCAAAAGTCCAAGTACTGTTCGGATTT
TTCAAACAAAAGTAGTTCCTAATTTGTTTTTCCCTAAAACTGGTAGAACAAATGCAGAAAGAACTGCTTGATAAATAAAA
CTACAATAGGCACTGTTTGAATCCTTTCTTAGCTAGTATATCAGCATTCTGCTCTCTGAAACAGTGAAACTACNMTGGAT
TGCTTGGGCCGTAGCTGGTGCATCARTAACCTGYAATCATTAACTATAGTCCAATAAGTTTGCTTAGAGTCATTAATAAT
GTTAATARACTCTGTAGAGTCCACTTCAATANNNNNNNNNNNNNNNNNNNNNNNNNNNNNGGTAACCTTTWTTCTTGTTTTCT
TGAGCAGCTAATTTATTTGGAGRTCTTTCTTTCTGCTATGGTCTACATGATCTACAAGAGTAGACTACTAAGCTTGAAGA
TTCTCGGGAATGAAGATCAACTKAWGTATACCATATTATTACTTGCTGAATGAAGGAGAATCTTGAATATTATATTTTAA
GTGGACATGTACTTTTCGCTTAATGTTCGTTTTTCTGCCTGTGTACTATCAGGAATTAAGCTTCTTAAGAAGATAACTAC
TGTTTCGCTCAATTATGTTCTTAATATTTTTGTGGTAATTTGGATGGTTCATATCACTCCATATATAAGGAAGCCAGGAA
GAGATAAATGGGATCACTAATTGTCTGTTTCTCTTACTTGCTTGCATTCATTTTCATGTATCAGAGGTGTTGGTAGTTTT
GAATTTGGGTTATGGATTGCCCATATGGACTGACCGACCCGATTTTGAAAGGACAATAAAAGGAAGTTATGTGGAGATTA
CCCAGATTAAATAAACTACCGAGTTTATTTGAACGTGGGTATGTGGGGATAAAACTATATGAAGACTTGCAAAATAACTC
CTCCTTTGCCGAGCATAGAAAGTGTAGGGTATCTTTTTGGGCAATTTACATAAGTATACAATCCAACACTCTTTTTTTAC
ACAAAAATGTCAATAATTTCCTATTTTCAAGATATAGCTACATTTTTTTTCAATACATAGATAACATATAAAAGGGTGCT
AAAACCCTAAATCTTCCTCCTACCACTCCCCCAGCCGCCACCCCCATCACTGCCTCCGCCTCCGGCCACAGGCCGCCTGA
AAATTCTGTTTAATATTTTTTTTGATGTTTTTTGTATATGTTAGTGTATTTTTATTGTATGCACATGTATATTCTTTGTA
TGCACAGAGCAAATACAGTAGGCATACTATGAAATACAGTAAGTATATACGAATACATTGTTATATGTCATGTGTATATA
TTTGTATGTCTATTGTATCCATTCAAAATATAACTCGTGCATACAGTGTTCGTACATTGAATAAACAACACATACACTCG
TCATTAGTGTATGTCTAGTGTATATGTATGCCTATTGTATGTCCCTTATATATACAAAAATACATAGGATACATACCAGT
CATACAAAATTAATATACTATGCATACAATATCTATACACCTTGAGCTTTCAGATTTCGAAGTTTCTTCTTCGTCACCAG
AAACACGTCATTGGAAAAGCTCAAAAAAGAAAAAACAATAACGAAGCAGCAAAGCTGTTGTAGCTGCATTCTTGACATCT
TGTTGACGTATAGATCACCCTTTTGACAAAACCAAGGTTAGAGCCACACAAATAGTAGTAGGCTTGACCATAGGACTAAA
TGTGTCATGATAGTCTATGCCAGCTTCTTGGTTAAACCCTTTTGC

Table S4. Nucleotide sequence of A-class genes from ‘Duo Lavender’.
This table presents the nucleotide sequences of A-class genes identified in ‘Duo Lavender’ (DL).

>*DL-ROB1* 13591805
ATGTGGGATCTAAATGATTCTCCAGATCAAAGAAGAGAAATCAATATTGATGAATCCGAAGAAGGTTGTTCTTCACATAT
TGAACTAGAACCCGATGATGAAAAGGGTAAACGAGTCGGATCTTTTTCGACTTCAAGTTCATCAGCAATAGCTATAGATG
AAATTTCAGAAGAAGAAGATGGAGAAAAAGGAAAGAAAAAAAGAAGTTCTCCTAGTAAACTTTTCGGCTTTTCAATGGTC
GGTCCGGGAGACTTAGAGCAACCGATAACGAGACAATTCTTTCCAGTTGATGAGGCTGAAGCTGAAACAGGTGTTGTAAC
TAATGGATCCCTGAATTTTCCAAGAGCTCATTGGGTTGGTGTTAAATTTTACCAAAATGAGCCACTTGGCATCACCGGTG
TGGTTGATGTAACTCAACAGCAGCAGCAACAACAACAACCTATGAAAAAAAGCCGTCGTGGACCAAGGTCTAGAAGTTCA
CAATATCGTGGAGTTACCTTTTACCGGAGAACTGGCCGGTGGGAATCTCACATATGGTAAATCTTATTTGTTTCATTTCA
CGCTCCAAATTCTTGATGATTCATAGCTTACACAAATTAGAAGTACGGTCTACGTACATATCATTTAAGATCACACTTGT
AAAATTATGTTGTATATGTTATTCTTGTTGTTTTAATTTGTAATTAGCTCAATTTATTTAGTGCTTGTTTTTCTTATTTG
AGTTAATATTATTTCAGGGATTGTGGAAAACAAGTTTATCTAGGTATGTGATGTTCATTATATTTACAATTAAAGGTTAG
TAATATCAGTTGCTCTTGTTGTATTCTTGAAAGTTAACTGATATTATTTTTAATGGGGTCTGCTTAGGTGGATTTGACAC
GGCACATGCAGCAGCTCGGTAAGCTCCTGGTTCTTCGCAATAATACGTTTGATGAATTTATTTTCATTTTGCCTCTTATT
TATTACGACAACAAAATAATTAAATTATTACTGTATTTATTTGTTTATGCAAAATATTGGTGTATTTGATAAATTATAAC
AATCAAACTTTTACAGGGCATATGATAGGGCAGCTATCAAGTTCCGGGGAGTTGAGGCTGACATAAATTTTAATTTAGAA
GATTATGAGGGTGACTTGAAACAGGTAAAACAAAGTTTTAACTATCAGATGACTAGCTAGGAAGAGGTGTGCTATTTTTA
TGTTTTAGCATGTTTTAATTCTGCTTGTCTAGTGGAGTACTTTTGTTTAATTCTTGCCTTATCTTAAACAGTGGTTTTCT
TATCTTCAACAGTTATGTACTTTACATGTTTGTGTCAAGAGTTGGTGGATACCAAGGAAATGTAAGATATTTTTTTTAAA
TTTTACTTTTTAATGTATGATACAGATGACGAATTTAACAAAGGAAGAATTTGTGCATGTGCTACGAAGACAAAGTACTG
GTTTTCCAAGAGGAAGTTCCAAGTATAGGGGGGTTACTTTGCACAAATGTGGTAGATGGGAAGCTAGAATGGGACAGTTC
TTAGGCAAAAAGTAAAAAATCCTATTCTTATAATATTCCTTTTATTGGTTCAATCCATTTTCAAAGAATAATCCTTTTAT
TGTCAATTATATTCTTGCTTTGCTAGATATTTAACTTCTTTTTTTTTTTTTAAAAAAATTTTTTAACAGGTACGTTTATT
TGGGCCTCTTTGATACTGAGGTTGAAGCTGCCAGGTGTGTGGTTGAAGTTCCAAATTTATAATTTAATTTACATTGCTTA
TTCTCAAATTTCTACAAACCTTAGTCCTTAAATTACTCTTCCTTTTTTTTAAATTGTTTCTCCTATATTCATATTTTATA
GGGCTTATGATAAAGCTGCCATCAAGTGTAATGGGAAGGATGCAGTTACTAACTTTGATCCTAGCATTTACGAAAATGAG
CTAAACTCAACTGGTATAATACCATACCAACTATACTGATATTTGCTTTCCTCTTGATAAATCACTATAATTTAAGATAA
AGTAGATTATAAAAAATAATCAAGCTGTAAAATTTTAATTAATGTTATTTTCCAGAATCTACAGACAGTGGGGCAGATCA
CAATCTCGACTTAAGTTTGGGTGGTTCAAGCTCAAAGAAAAACAATAGAGAATTTGGGGATAATAGAGGTCAAAATCCTT
CCTCAATGCAATTCGATGTTGATTGGAGGCATAACGGGTTAAGGCCTGAGGTACTTGCATAAACAATATTGTATCTTAAA
ATTTTTGCACGAGTTTAACTAATATACACAAATTATATAGTACTAGTATATTTTTTTTACACTATCATGTCAATAAGCAT
GATAAAAGACAGTTACAACGGCTAAAGCTAAACTAATAGTATAAAGTTCTTTTTATGCTATCCGCCTATGTAAATTAAAT
TCTCGTTTCCTTGTGGATTTATCTTTTTTTACCTTATATACAACGATGTTTTGATTAATTTCCAGAAGCAAACTGCACCA
ATTGATATGGATGCTCGACGAAGAGATAATGGGTACAATGAATCAGAAACGTTGCAGTTATTGAGCAAAACGCACCTACA
TTCTCCAGTCTCTTTGAAGCATAATAATAGTCATCAATTACAACGGTTTGGCCAATATATGAGACCTGGTGAATCCCATA
TGATTCAAATGTTTCCACCACAATTCGGCTCATCAAATTATCAGGTAATTATCCAAATTCATATTTTATTACCATACGGT
TGATGTATTCCGCCTATCTTTCCGAGTCCTTTTTAATTGTTGTACCATGATATATACAGATTCAATTTCCAAGCGGCAGC
AATGGAGGTCGAATTGGAGCTACAAATGTAGGAGATTTATCGCTCTCGAGCAGCAATGCTTCTTCACAATGGCAATCCAA
TTTACCTCCTCAAATATTTGCAGCTGCTGCAGCATCATCAGGATTCTCCCAGCAGATAGTAAGACCTCAAAATTGGTCAT
CCGAAAATGGCTTCCATCACTCTCTTATGAGACCCTCATGA

>*DL-ROB2*(downstream)_(reversed)_ 13378572
TATTTTAACTGTTCTATGGACTAATTTTTACTTTCTTTTTTCTCCTTTATCTTTATTTAATAGGGCTTATGATAAAGCTG
CTATCAAGTGTAACGGGAAGGATGCAGTTACTAACTTTGATCGTAGCATTTATGAAAATGAACTTAACACAACTGGTACA
TTACACTTTTCTTTTCTAGCTTTTGATTTGGTCTTTGTCTGCCAACATATTACTGTATATATTATTAAAATAAAAACCTA
GAACTCATAAATGCTTCTTTTTTCAGAATTTACTGATAATGCAGCAGACCACAATCTTGACTTAAGCTTAGGAAGTTCAA
GTTCAAAAGAAAGCAGTCGAGAACTAATGGGAGATAATAGGGGTCAAAATTTTCAATTAGATGTCGATTGGAGGAACCAA
GGGTCAAGGCCTAAGGTATATACTTTTATATCTATAATGTATTTTAGTGGCGGATCTAAAGTTATATTTAAGGGGGTTCG
TAGAACCAGAAACTTTTACCAAAATCCTATATTTGTGTTTCAGAAGTCATAAACGTAAAATCTTAGATCCGCCCCTAATG
TACTTTAGACGGTGACGTTTTTATTTTCACATATTACTCACATCATGCTCTAAATGTTTTAATTTCCTGCAGCTACCTAA
TCCAATTGGGTTTGATACTCAAAGAAGAGGTGGGTACAATGAGTCTGAAACCTTGCAGCTCTTGAGCCACACACACATAC
ATTCTCCAGGCTCCTTACAGCCTAATGTAAATGAAATGCAAAGGTTTGGCCAATTTACTAGAAATGGTGAATCCCATATG
GTTCAAGTGTTTCCTCGACAGTTCAGCTCGTATCAAGTGAGTTTTAATCCTATCATTACCACTTTGTTGAGATATAATAT
AATTAAATAATAAACAGTTTTAGAAAGCACATATTTTGTTTCTTAATTATTTTACATCTCAACACTCTCCTTACATACAT
GTGTGCCTAATTCGTTTTCATTGATCAAATTAAACATGTGAATATATTTTTTGCTTTTAGAGTGGCGTTCAATTTAATCC
TAAGACTCTTATCTCCTCTGATATCATGTCAAATTATGCGAGGTTAAATTGTCAGAAAAAGTATAATTCTAGTTACTCTC
TTGGTTCCACTTTAAAATAGCACTTTACTGATTGGAGAGGTAGCCAACAATTTTTTCTCTAACTGCAATTTATCAAATAT
TTCTTGAATATTGTGAAATATTAAATATTCAGACTTTTAATAAGTTATAATTTTTATTAACTTCTTACTATGTGGAGGAA
TATTTTTCTTTAAAAACTTGAAGATTTTAAGTCCAATTTCACAACGAAAAGGAAGTGATTGACTTCAAACTCATGAAATC
ACATAAAATGAAATAGAGGGATTACTTTATTGTATCATGTCTTGGCATTTTTGAGTTTTTATGTATCAACCCATGTTTTA
AAAACTTGTATTGTATACAGACTCAATCGCCAAGCAGCAGCAATGTAGGCCAAATTGGGACTACAAATGCACGAGATGTG
CTTTCACTCACTACAAGTAATTCTCAAGAATGGTACCATCCCAATATTCCTCCTCATCATATATTTGCAACTGCTGCAGC
ATCATCAGGATTCCCCCAGCAGATAGTAAGACCTCAAAATTGGTCTCAAAAAACTGGCTTCCATCACACTTTCATGAGAC
CCCCTTGA

>*DL-ROB2*(upstream)_(reversed) 13605178
ATGTGGAATTTAAATGATTCTCCGGATCAAAGAATGGAAGAAGAATCAGAAGAAGCATGTTCATCGCCAATTGAACTTGA
TGATGATAAAGGTAAACGTGTTGGATCGGTGTCAAACTCTAGCTCATCAGCTGTAGTAATTGAAGATGGTAATAATAATA
ATAATTCTGAAGACGAAGACGAAGAAGAAGAAGAGGATGAAAAAGGTAAAAAGAAAAGAAGTATACATAATAAACTATTT
GGATTTTCTGTAATGGATCCGAATAATTACCGCGATTTATCGGAGAGTGAACAGCCGGTTACTCGGAATTTTTTTCCGGT
GGACGAGTCAGAAATGGGGTCTAGTACAAATTTACCAAGGTCACAATGGGCTGGAATTAAATTTTATCATGAATCGGAGA
CACGTGGCAGCACTGTATTGGCTGGAAACCCTACTGAGATTGTTGTTCAGCAGCAGCAGCCAGTTAAAAAGAGTCGTCGT
GGACCACGGTCTAGAAGTTCACAGTATCGTGGTGTTACTTTTTACAGGCGAACTGGCCGGTGGGAGTCACATATATGGTG
AGCACTTTTTGACATTTTATGTACACTGTTTTGCATAATAACGGATTCAAAATTTAAACTTGATAAATTCGAACTATTAA
AATTAGTGCACTTTTACATTTGTCCGTCTAAATTTTTCTATTTGTTTGAAATTTTGCGATATATTGAACTAAAAATAATC
AGTTCAGTTGAACGCATCCGCCTCGGTATGTTGATCAACATTTTCTTCCTACTATTCTCTCTTATAGCTGAAATTTATCA
ATGCTTACTTTTCTGATTTTGCTTTGTTTTGGTTTATATGATTTCAGGGATTGTGGGAAACAAGTTTATCTAGGTAAGTC
TTGAAAATAATAAATGTATAGTTGAATTGAAGCTTTTAAGTTTATCAAGAATATTATTTGGTTGTTGTGTATGTTCTTGA
AGATCAATCTGATATTGGTGAATAGGAGTTTAATTTCCTCGGATATCATATGTGTAGGTGGATTTGATACAGCACATGCA
GCAGCTCGGTGAGAACTTGATTCTTGATTGTAATTTTTGCTCCAATTTTTTCCAATTATCATTGTCTTATAGAAAAGAAA
CTAAAGATGAAATTTAAAAGTAATTTTTACTATTTTTGTGACCCTTTTTATTTCAGTGCATATGATAGGGCAGCAATCAA
GTTTCGTGGAGTGGAGGCAGACATAAACTTTACCTTGGAAGATTATGAGGAAGACTTAAAACAGGTATGTTTTTATTCCA
TGTTGTTCTAATCTTTCCAAAATGTCTCTGCACTTGTGTCGGATCATTGAAATTGTGCATTTTTTAAGTATCTGACATGG
GTGTGACATTATTTTGGAGTTGAATTTTACCCTCATAAGTCATACCTTATCATAAACAGTTGTCTTCCTTATCATCAACA
GTAGTTAGGTTCCCAAATGTAGTGTCAAATTTCAGTGAAATGAATATACTTTTCTCTCCAATAATTTGTTTTATCTTTTT
GTGGTATATATCACAGATGACCAATCTAACAAAGGAAGAATTTGTGCATGTACTAAGGAGACAAAGTACTGGTTTTCCAA
GAGGAAGCTCAAAGTATAGAGGAGTAACTTTGCACAAATGTGGTAGATGGGAAGCTAGAATGGGACAATTCTTAGGCAAA
AAGTAAAAAGAAAAACAATCTTATCCTCATTTTATTCTCTTGTATGATCAAATAAATTTTCAAAAATGATCCTTTTGTTG
TCAACTGCACTAATATTCCATTCCTTTCTTGCTGTATTGACTTTTCCTTATATGATCTTTTCTAACAGGTACGTTTATTT
AGGCCTCTTTGATACTGAAGTTGAAGCTGCCAGGTGTGTGATGTTCTTCTTTCTTTACTCTTTTAGTTCTAATAATGGTG
TCGTCCAGGTCAACCTGTGGGTTCCTCAACTATTCCACCAAATATTTGCTAGCTTCTATTAGCATATGTACTGCGTAACT
CTGACCACCAAGATTTAGACAAATAAAAACAAAATCACCGAGTATTCAATCTTACTGGAATTTGAACATGTCTTGATTTT
CGAGGTTTTCACCCGCTTCATTGACCAAACCACACCCATGGGGTGCCTGCTGTTGCAATTCTTGCACTCAAACTACTATT
ATTAGTGAGGTTGAGCCTTGAGGGTCTTGTGTATTTTAACTGTTCTATGGACTAATTTTTACTTTCTTTTTTCTCCTTTA
TCTTTATTTAATAGGGCTTATGATAAAGCTGCT

>*DL-ROB3*
GAAGATGGTACTAGTAATTCATCTATATCAATATCAAGACAAAAAGGTAAAGAAAAGAAAACTACTACTACTACTCATAG
TAATAAATTATTTGGCTTCTCAGTTATTTCTCCTAATAATAACAACAACAACAACGACAACTTGTCGTCGGAAAGTGAAC
CGCCAGTTACCCGGAACTTTTTTCCGGTTGATCAGTCAGAAATTGGACATGCAACTGCAAGTACATTTGATGATAGATCA
TTTCCAAGGGCCCATTGGGCTGGAATAAAATTATGCCAGCCCGAATCACCAGGAAATTCATCGATGGGAAAGAATACTGA
GTTGTCGCAACAAGTTCAGCCTATGAAGAAGAGCCGGCGGGGACCAAGGTCTAGGAGTTCACAGTACCGTGGTGTTACAT
TTTACCGCAGAACTGGCCGGTGGGAGTCACATATATGGTTAGTCCTTAAATATTACTACTATTATTATTAATCATCTTGT
TAAAGTGTATGTATATTAATCATTTGGTCTTGTTGAATTGTGCGTAAGCGTAACTAATCGTCTTAAAGTTTTAACCGTTA
GAAATATCACATGTTGTAAAACGTTTTCCTTGACAATAGGTTTAGGTGGTGAGATATGAATAAGTATTTAACAATAAATG
TGGTGATAATTGAAATAAAGAGAAGTCTTGTTTTATATAAATGGTGGTGAGAATTGAAATGAAAATTTTGTGTTTCTCTT
TCTAAAAATAAGTACGGGGAGAATTCAATTCATGGTTATTGATTAAAGACTTTGTGACAAGCTTGGATTTATACATACAC
TTGGCTTTTCTCATTTCACTTGTATTATTTCTCAGGGATTGTGGGAAGCAAGTTTATCTAGGTACTTATTTTTTCTGTCA
TTTATTTGTGAAAACTGAAGCTGTAAGATTTATAATCTGCTCATCCTTGAAAATTAATCTCACATGATCAATTTTTGTAG
GTGGATTTGATACATCACATGCAGCAGCTCGGTGAGTTCTTTGATTCTTGACCTTCATAATTTTTGCTTGCACTTTTGTT
ATTATTGTTTCTTTGAAATATGAGATAAAAATTTGAAAAAGATCAAATCTTTCGGAGATTAATAAAGATTCAAAAAATGT
GTTTCCAGTGCCTATGATAGAGCAGCAATAAAGTTCAGAGGAGTGGAGGCGGACATAAACTTTAACTTGGAGGATTATGA
AGAAGACTTAAAACAGGCAAGTTCATGATCAGATCAAGGTGACAAGACTCTTAAAGAATGTGTTTTCCTTTGTGCCTTTC
TACTTTCTAGCTGGAGATCATCTATACTTTAGTTTTACTCTTTGCCTTATCTTCAATAGTGACTCCTTATCTTCAACAGT
TGTTTACTTTACATCGGAATCTTAAAGATGGATTCAGAATTTAAACTCAATATTTGCAGTTTTAGGTGGTTATTACTGAA
CTACATTTTCTAAATTATGGGATCAGAATGATATTTATTAAATAATTTTCCACATGTATATTTATGTGCCGTGTAAAAAT
TGATGAGTTTAGATGTGCCAAATGAATAGATGCTTCATTAGCTTCACCAAGAAGCATTTGGAGTATCTGTCTTATGCGTT
GAACTGAACTTAGCATTTTCAGCACAATGCAAAAATGATTCACACCATGAAATTGAATGCAAAAATATTATAGKGACTTT
CTTTTTATGTGTTCTAMATMATCTTTTTGGTTTGTTGTGTGATGCAGATGAGGAATTTAACAAAGGAAGAGTTTGTGCA
TGTACTTAGGAGACAAAGTACTGGTTTTCCGAGGGGAAGTTCCAAGTATAGAGGGGTGACTTTGCACAAATGTGGTAGAT
GGGAAGCTAGAATGGGACAGTTATTGGGCAAAAAGTATAAAATCTTTAATCTAATTTAATTCTCCTAATTTTCAAGGAAC
AGTCCTTTTGTTGCAATATTAGTTCCTCGCTATTGACTTCCAACTTTCTAATTCTTTTCTAACAGGTACGTTTATTTGGG
ACTCTTTGATACTGAAGTTGAGGCTGCTAGGTGTGTAGTTTATTACTAATCTATCAGTATAATTTAATACTACTAGTATT
TCTTAGCGTTCTTTTTAACTACTGAACTAAGAAAACCCTTAACTTTTTCATAATCATTTTCATTATAGGGCTTATGATAA
AGCTGCCATCAAGTGCAATGGGAAGGATGCAGTCACTAACTTCGATTCCAGCATTTAACACATTATTAATACCCACATTA
CTAATACCCGCATTAATAGTTAATACCCGCGTAAAAGGAAAGGCAAACCGAACGACCCCATATTACTATGTGCTTCTTTG
CTTCCTATTTAGTCATGTGATGTAAAGAAAAAAGAAAATTAGCTGACAAACATTATTCATAAATATTGTTGCAGAATCTA
CTCATAAGGCATTAGATCACAGCCTTGATTTAAGCTTGGGTGGCTCTAGCTCAAAGCAAAGCAGCAAACAATTAACGGAG
GATAATGAGGATCAAAATTATTCTTCTGTCCAATTTGATATTGATCGGAGGCACCAAAGATTGAGGCCTAAGGTACCTAC
ATAACTTTTCTAGCTTAATTTCTACCAAACTCTCTTGCACTTTTTCTTCTGCATATTATGATAAAGTTTACTAAGGGTTA
GCTGGTGCACGATTTTAATATACGATGGATAATTAATAAGCTTACATGTCTATCCATTTTAACTTAAATATAAGACATTA

GGCATGAAGAAGTTCGAACATGTGACATGCATCTAACTCACACATCATGTGTTATGCTCATAATTATCGCAAGGTGAAAG
TGCTAAGGCACCTTGTAGTTGGTGCATTTATATTGATACCCCTTATGTTCCAATTTTTTTGGTCATAGTTTTACTTGATT
TGCGAAATTTAAGAAATGAATGAAAATTTCTAAAATTTATTGTTTAAAACATGTCATCGCATCATTTTTATGGCTATAAA
ATTTTTCATTAAGTTGAAAGTGATAAGTTGAAAGCTATATTGTTCCAAATTAAGAAACCGGACATTCTTTTGGAATAAAC
AAATTAGAAAATTGTTTTTCCTTTTTTTGCTCCTTATACATGTTTTAATTTCAGCAGCAAACTAGTCCAACACGAAGAAG
AGATGGGTACAATGAGACGGAAACCATGCAGCTCTTGAGCAAAACGCACCTACATTCTCCAGGCTCCTTGAAGTCAAATA
AGAATGAAATGCAAAGGTTTGGCCAATATATGAGAGTTGGTCATGAAGCCCAAATGATTCAAATGTTTTCACCACAATTC
AGCTCGTCAAATTATCAAGTAAGTGCCCATTTCACATGAGTTTAATTCCATGCATTGACACTACATATAGGATAACTATA
CAACTAGGTCATGGCTTAAAAAAATAGTAACTATAAATAACTCTCCATAATGAATATGATTAATTATGTATCTAAGAAAA
TAAGACAGATAACCTGCTAAACAAAATACATTGATAAGATAAAAATTCTTTACATATGCATGTATAAAAATAAAGAGTCT
AACCTCTATAATACAATGTATTTTAGGTTTACTAGAGATAAGTACTTGTACCACCCATAAGAACTGGGAATCTTAACTTG
GAAAATAAGGTAAAATTACCAGCTACTTACAGCTCGTTAAAATTCAATATTAGTCAATAGTGTAAAATTCCTTATATGTA
TATAGGTTTTTTATTGTTTGATGCTATACTATAATTGCTATTGGTATCTGTACTTTTTGTCATTATTAACGTAGTTGCA
CTAAGAAAGTTTATTTTATCATCCTTGTAAAGTTTGTTCAAATTGTTATATGATACAGAACATTCAGTTCCCAAGCAGCA
GCAATGTGGACAGGTATGCTGGACCTAATACGAATAGAAGAGAGCCTATGTTCTCATCAAGTGATACTCAACAATGGCAA
TATTCCAATACTGTTCCTCCTCAGCTATTTGCAACTCCTGCAGCATCATCAGGATTCCCTCAGCAGATAGTAAGATTTCA
AAATAATTGGTCTCAGAAAAATGGCTTCAACTATTCTCTTATGAGGCCTTCTTGA

>*DL-162Scf00072g00229.1_1* 13873492
ATGAAGAAAGTGATGTTGGATCTTAATGTAAGTATAATCAATAATTACATACTTGATCAGAACCTTCCACAAGTTTCTCC
ATCATCAGGAACCTCCAATTCATCCATACAAAATGCAGAGGCAACAAGCAGCGTCGACGACACGTGCTCTACACGCGCCG
GTACCTCGTTCGTGACTCGGCAACTCTTTCCTATTGAGTCGAACCGGGAACAGGACTTAACCCGGTCTGATCGGGTGAAT
TTCACTTCCGGTTTCGGAAACGTGGTAATAGTACAACAACAACAGCAGCAGGAACAACAACAACAGCAAGCGCATGTGAA
GAAGAAGAGTAGGAGAGGACCAAGGTCAAGAAGTTCACAATATAGAGGAGTTACTTTTTACAGGAGAACTGGTAGATGGG
AATCACATATTTGGTTAGTTAGTTAGTTACTTAATTCTCCAGCTGTAATTTTTTTTACTGCATTTTATTTATCGTCGTTT
TATTTAATATAATTAATTTTGGATGCAGGGACTGTGGGAAACAAGTATACTTAGGTATGAATATTGCTATTTGAGTTTGA
ATTGTAAATAAATTTTACACTATCATGTCAGGTTTACCTGTCGACGGTTGGAACAGTTAACCTGTCTTATTATTCAAGTT
TCATTATGCTTGCTATAGTATTAGTTATTACAATCTCCATTTCTGTTTATGTTACCTTTAAATAGTCAATCTCCATGAAC
TTTGACCAATATTCTAAGATATGTCTTTCACCATGTTAATATAAGAAGAGTTGCAACCTATAGTATTTCTCGTATAGTTT
TGCATATCTGAATTTTAATTATAAAATATTAAACTTATCTTACCCAATTTAGCTTCAAAGATTAGCCACTTGACTTTCAG
AAAGGAGAAAGGTCACATAAGTTGGAAAGGAGGAAGTACCAGATTTAGTTAATTTCCTTGTTTCTGTGGATTTTGATGCT
AAAGTTATTATTAATGTCTCTACTTTTTTCTATTTTTATTTTGATTGGTTTTTGTTGTTGGTTTATATTAGGTGGTTTTG
ATACTGCTGATGTTGCAGCAAGGTAAAGTGCAAAAGCATAGTTCGCAAGCATGCTTCCAGTTTTACTTTATTTCCCGAAT
AAATTGTTCCTTGATTTGATTGTATTGGTTTTCTTCTCGATTTTATAGAGCTTATGACAGAGCTGCAATTAAGTTCCGGG
GTGTTGATGCTGATATAAACTTTAGCATAACGGATTATGAGGAGGATATGAAACAGGTTAGAGATACAAAGATTGAATTT
TTGCAAATATGTTATGGTCACATTTGAACCTTATTTCGAGGCGGATAGAGAATTTGAATTTTATGAGTTCGAGTTCTGGA
ATCTCCCACGATCCATTTGATTTATTAGGTTTGAAATCAATTATCTTTACTTGATGATTTTTTCAACAATACACCGGGTC
TGAGCCAACACTAGTTGGTTCGAAATGAACCCATAGCTTATACTCTATATCTGCTTCTGTCTTTGGTTTCATATTATTTC
TTTATTAATTGGGGTGCATTATATGAATCATTTAAATTCTATTCTGTATTCATTGGCCTTATTTCTGGACTGATGCAGTT
GAAACACCTTGGTAAAGAAGAATTTGTTCACGTGCTACGACGCCAGAGCAATGGTTTCTCAAGAGGGAGCTCCAAATTTA
GAGGTGTGACACTGCATAAATGTGGCAGATGGGAGGCTCGGATGGGGCAGTTCCTCGGCAAAAAGTAAGGAACTAAACAC
ATTGAAATTCTTGTAAGGGTAGATTACAGCTTAGGTTATACTAGTAGTTTGTCCAAACTGGGAAATGATTCTGTTAAACA
CGTGTATTTGAAAAGAAATCGCTGGTACTGTGGTATTCATGGTGGGGATTAAACTGCTTACATTGCAGGTATATATATCT
TGGGCTATTCGACAGTGAAGTAGAAGCTGCAAGGTCCTAAGATCTTGAATTACCCTCTCTGATAATGAGTTGTTAACCTA
AACTCTCAACTTCAATCTTGTGTCATCCAACCTCTATCTCCCTTTTCCTTTTCTGCAAAATAGGGCTTATGATAAGGCTG
CAATCAAAACTAATGGAAGAGAGGCTGTTACCAACTTTGAGCCAAGTGCATATGAAGGGGAAACAATATCTGAACCTCAG
AGTGAAGGTTTGCCCAAAAGCTCTTGCTCATTTCCAAACTAGTGCAACTAATCCGAAGCAACATGCATTACTATACCTTG
ATCTATTGCATTTGCAGGTAGCCATCATAATCTTGATTTAAACTTGGGGATATCGACCTCTTCTTCAAAGGAAAATGACA
GGTTTGGAGGGAAATACTATCATCCTTATGATACGCAAGATTTAACAAAATCAAAG

>*DL-162Scf00072g00229.1_2* 13481368
ATGAAGAAAGTGATGTTGGATCTTAATGTAAGTATAATCAATAATTACATACTTGATCAGAACCTTCCACAAGTTTCTCC
ATCATCAGGAACCTCCAATTCATCCATACAAAATGCAGAGGCAACAAGCAGCGTCGACGACACGTGCTCTACACGCGCCG
GTACCTCGTTCGTGACTCGGCAACTCTTTCCTATTGAGTCGAACCGGGAACAGGACTTAACCCGGTCTGATCGGGTGAAT
TTCACTTCCGGTTTCGGAAACGTGGTAATAGTACAACAACAACAGCAGCAGGAACAACAACAACAGCAAGCGCATGTGAA
GAAGAAGAGTAGGAGAGGACCAAGGTCAAGAAGTTCACAATATAGAGGAGTTACTTTTTACAGGAGAACTGGTAGATGGG
AATCACATATTTGGTTAGTTAGTTAGTTACTTAATTCTCCAGCTGTAATTTTTTTTACTGCATTTTATTTATCGTCGTTT
TATTTAATATAATTAATTTTGGATGCAGGGACTGTGGGAAACAAGTATACTTAGGTATGAATATTGCTATTTGAGTTTGA
ATTGTAAATAAATTTTACACTATCATGTCAGGTTTACCTGTCGACGGTTGGAACAGTTAACCTGTCTTATTATTCAAGTT
TCATTATGCTTGCTATAGTATTAGTTATTACAATCTCCATTTCTGTTTATGTTACCTTTAAATAGTCAATCTCCATGAAC
TTTGACCAATATTCTAAGATATGTCTTTCACCATGTTAATATAAGAAGAGTTGCAACCTATAGTATTTCTCGTATAGTTT
TGCATATCTGAATTTTAATTATAAAATATTAAACTTATCTTACCCAATTTAGCTTCAAAGATTAGCCACTTGACTTTCAG
AAAGGAGAAAGGTCACATAAGTTGGAAAGGAGGAAGTACCAGATTTAGTTAATTTCCTTGTTTCTGTGGATTTTGATGCT
AAAGTTATTATTAATGTCTCTACTTTTTTCTATTTTTATTTTGATTGGTTTTTGTTGTTGGTTTATATTAGGTGGTTTTG
ATACTGCTGATGTTGCAGCAAGGTAAAGTGCAAAAGCATAGTTCGCAAGCATGCTTCCAGTTTTACTTTATTTCCCGAAT
AAATTGTTCCTTGATTTGATTGTATTGGTTTTCTTCTCGATTTTATAGAGCTTATGACAGAGCTGCAATTAAGTTCCGGG
GTGTTGATGCTGATATAAACTTTAGCATAACGGATTATGAGGAGGATATGAAACAGGTTAGAGATACAAAGATTGAATTT

TTGCAAATATGTTATGGTCACATTTGAACCTTATTTCGAGGCGGATAGAGAATTTGAATTTTATGAGTTCGAGTTCTGGA
ATCTCCCACGATCCATTTGATTTATTAGGTTTGAAATCAATTATCTTTACTTGATGATTTTTTCAACAATACACCGGGTC
TGAGCCAACACTAGTTGGTTCGAAATGAACCCATAGCTTATACTCTATATCTGCTTCTGTCTTTGGTTTCATATTATTTC
TTTATTAATTGGGGTGCATTATATGAATCATTTAAATTCTATTCTGTATTCATTGGCCTTATTTCTGGACTGATGCAGTT
GAAACACCTTGGTAAAGAAGAATTTGTTCACGTGCTACGACGCCAGAGCAATGGTTTCTCAAGAGGGAGCTCCAAATTTA
GAGGTGTGACACTGCATAAATGTGGCAGATGGGAGGCTCGGATGGGGCAGTTCCTCGGCAAAAAGTAAGGAACTAAACAC
ATTGAAATTCTTGTAAGGGTAGATTACAGCTTAGGTTATACTAGTAGTTTGTCCAAACTGGGAAATGATTCTGTTAAACA
CGTGTATTTGAAAAGAAATCGCTGGTACTGTGGTATTCATGGTGGGGATTAAACTGCTTACATTGCAGGTATATATATCT
TGGGCTATTCGACAGTGAAGTAGAAGCTGCAAGGTCCTAAGATCTTGAATTACCCTCTCTGATAATGAGTTGTTAACCTA
AACTCTCAACTTCAATCTTGTGTCATCCAACCTCTATCTCCCTTTTCCTTTTCTGCAAAATAGGGCTTATGATAAGGCTG
CAATCAAAACTAATGGAAGAGAGGCTGTTACCAACTTTGAGCCAAGTGCATATGAAGGGGAAACAATATCTGAACCTCAG
AGTGAAGGTTTGCCCAAAAGCTCTTGCTCATTTCCAAACTAGTGCAACTAATCCGAAGCAACATGCATTACTATACCTTG
ATCTATTGCATTTGCAGGTAGCCATCATAATCTTGATTTAAACTTGGGGATATCGACCTCTTCTTCAAAGGAAAATGACA
GGTTTGGAGGGAAATACTATCATCCTTATGATACGCAAGATTTAACAAAATCAAAG

>*DL-162Scf00389g00028.1* 13932355

ATGATGTTGGATCTTAATTTAAGTGCAATTTATGATGAAAAAGTAGGAGAAATTGCCGTAGCTGATGAATCGGGAACCTC
CAATTCATCGGCGAGGAATGCAGAAGCCTCCAGCAGTGCTGGAGATGATGACTCGTGCTCCACACGCGCTGCCGGAGACT
TGTTCGCCTTTAATTTCGATATCCTTAAAGTTGGTGGAGCTGAAACTAGTAGGAGTTTCAGTATCAATAATGATGAGGAA
GTGTATGATGAGAATCATATGAGGATGGCTNNNNNNNNNNNNNNNNNNNNNNNNNNNNNNNNNNNNNNNNNNNNNNNNN
CCTTATTGTGTAATCAATAGGCCCAAGAAATTTTGGGGCCTAAAGCCACTGCTTTAGTGGCTTTAGGGTTGGGCCGGCCC
TGGCTCGGCCCGATGTTGTGACTCAGCAGTTTTTCCCGGTAGATACTGCTGAGTCTAATCGGGCCCACCATACCTCTCGG
AGACCTGATTGGGTGGATCTTTCATACGATCCACCCAATACTCTTGGTTTCCGAGAAGTGGGAATAGTACACCCACAACA
ACAACAGTACCAACAACAACAGCAGCAGCAACAACCAGTTAAAAAAAGTAGGAGGGGACCTAGGTCCAGAAGTTCACAGT
ATAGAGGTGTCACTTTTTACAGAAGAACTGGTAGATGGGAATCACACATATGGTTTGTATGCTTTAACTATGAAATGTTT
TAAATTTCTTAATATTGTTAAAATTATTAATATGTGGGTGTTTTTTTTTTTTTTTTTTTTTTGTATAGGGACTGTGGCA
AACAAGTATATTTGGGTATGTACTACAGTATGTAATATTGTTATTTTAATTGTTATCTAATTGGTTAAGTTCATTGGTCT
GTGATCTTTTGGAGTTTAGTTATGCATATGCTATTTCTATTTTGGGAAATTTTCTGATTGGCTTTGTTTTTGGCTTGTTT
TTAGGTGGATTTGACACTGCTCATGCAGCTGCTAGGTAAAACATAAACAGTTTATATACGATATCTCTAATATTTTATAG
ATCCTTTCAGTTTTATGCTTTTTCCCAAGTTAAATTCTATGCTTGAATAGTTGCATTGATGAAGTGTTTCTCGATGTTTT
CTTCGTGATATCAGAGCTTATGATAGAGCAGCAATTAAGTTTCGGGGTGTTGATGCTGATATAAACTTTAACCTAAGTGA
TTATGATGAAGATATGAAGCAGGTTCGAATGTAAAGATTTTAACTAGTACTATAACATGTTAAGCTCATTGTTAAACATG
AATATGCATTGTACTGAATTTGGATTCGAATTGTTTGTGAACTGATGTAGATGAAAAACCTAAGTAAAGAAGAATTTGTA
CACATGCTGCGACGCCAAAGCACTGGTTTCTCAAGAGGGAGCTCGAAATACAGAGGAGTAACGTTGCATAAATGTGGAAG
ATGGGAGGCTCGGATGGGGCAGTTTCTTGGCAAAAAGTAAGAATCTTTATTCATTGAAATTGTTGTAGAAGTAGAAACTT
TATTTATTGAAATTGTTGTAGGAGTAGTTTCAACTAATGTTACTCAAATCCTTCGGAAGTGGCGTTGTACCCGTGTCGGA
TCCTTAAAAATGAATATTATTTGAAGATCTGACACAGGTGCAGTGGCACTTTGTTGGAGGATTCGAGCAAAATAAGGAGT
ACATTACATCTTGAGAATATGTAGTTGGTCTGAACTGGGAGACAATCTGTTAAGCATGCCTATTTGAAGAAAGAAATCAA
CGGGACTACAATATTCATAGTGGGCATTCCACTGCTTACATTGCAGGTATATATATCTTGGACTATTCGACAGTGAAGTA
GAAGCTGCAAGGTCCTAATGATCATGAATTTACCCTCTTCCCTGATAATGAATTTATTACACTAAACTTCTCAACACAAA
ATCTTGTATCAACCAACCCGTATTTTCTGCAAATTAGGGCGTACGATAAGGCAGCTATCAAATGTAATGGAAGGGAAGCT
GTTACCAACTTTGAGCCAAGTACATATGAAGGGGAAACAATCTCTGATCCTCGGAGTGAAGGTTTGTTCACAAGCACTTA
ACATGATCATTAGTTTTTTTATTATCTTTTCATTTTAAATATGTGTATCAAGCAGGTAGCCAGCAGAATCTTGATCTGAA
CTTGGGAATTTCGACCTCTTCTGCGAAGGAAATTGAAAGGTCGGGGGGTTTCCAGTATCATCCTTATGATATGCAAGATA
CAACAAAATTACAGGTTTAAATAGAGTAGTTTATTTTGAAATGTTGCAGCAGATTGAATTTGATGGTGTTTTACTTCCGA
GCTCTTCCTTTCATTTCTTTATACAGATGCAAGAGACTGTGAAACCGTTAATCTTGTTTTTTTAGATTGTGCTGATAATT
GGTTTCCTTTTATGCAGATGGACAAATCTGGTCCAGCAATAGTTAGTAGTTCACATTTTAAAGCACAGCCAGTGACATCT
GAACAAGCTCACTTGTGGAATGGAGTATATTCTAATTTTTTTCCCAGCTACGAGGTACAAAAACAGTTCACTTTATTCTC
TAGCTCTGTTATTCTTCTTTACTAGTTGAAACTTTCGATTTTCTAAAACTTTGGACCGACGTGGGCTTAGTAAGAGCGAC
ATATTCTGTGAGGATTCATGAAACCGAACACATGCTTGGATTTGAGGCATAGTAGTAGTTGTTGTACTTGTACATGTGCC
TTCAGTCCAAAAGAGCCATCTGCATGACCCCCTTGTTTCTACATTACAGGATTCTGTCATTTATGAAATAGTTGGTGGC
TGCAAGTCAATACAGTGTAGGTTCTGATTGTTGTGAACTCTACTGAAACTCTGTTGAAAATTCTTGTAAACATAAGATTC
TGCATAGGTTTTGTGAGGGCCTTTACATTCTTTCGACTGTTTTCCAGAGGGTAAATTGCCATTGATAGACATTCTATTCT
TTGTGGCTAAAGCATTTTATTTAACGGATTTAAATCATATGCCCTAACATTGTAAGCTGGTAAGGCTCTGGTCCTTCCCG
ACCTTGATGCATAGCCGGGAGCTTCGTGCATTGGACTTCCCTTTTATACTCTAACAACATGAATAGTATAAACTTGTCAT
TGAAGTTTAATTTCAGTTTTTTCTAGATGGTTACCAATCAATGCTCGGGCTAATTAACTAAATGGCTTAGCAGGAAAGAG
CGTCTGGGAAGAGAGTAGAAGTAGGTTCATCCCAAGGACCGCCAAGCTGGGTAATGCAAATGCATGGTCAGGTCGGAACA
ACCCCAATGTCAATGTTTACTGCTGCAGCATCATCAGGATTCTCATCTCCGGCTACCATTGCTTCGGCCTCTCCAATATC
TGGTCCTAACCCGAATATTCACAATCTATCATTTGCTACATATTCAACACCATCAACAAATACCTCTCAATACTTTTACC
AGATCAGGCCGCCGCTACCACCTCCATAA

>*DL-162Scf01024g00326.1_1_*(reversed) 13660615

ATGTTGGATCTGAATGTATCAGCAGTTTCTGTGAACTCAAATTGTGATGAAACTGACCCATACAACAATACCAACACGTT
CTTGAAAGATGATATTTCTGGAACTTCAAACACTGATTCTTCTTCAGTCGTCAACGCTGTTGTGGGAGATGAAGATTCCA
ACAGCTCTTCACATCAGCATGTGTTTAAACATTCTCTTTCAACTCTGAGTTTCTCCATATTGAAAAGTGATCGTGTTATG
GAAACTGAAGATGACATGACCAGTGATGATTATAAAACAAGGCAGCTTTTTCCAGTGAAAGTGGAGACGAAGATTCAAGA
TCAAGCTCAGTGCTGGCTTAACTTGTCTGTGCCGGAATCCCGGGGAGGGGCAGATATCGGAGTATACAAACCACCGCCGG

CCAAGAAAAGCAGACGGGGGCCTAGGTCCCGTAGCTCACAGTATCGTGGTGTCACATTTTATCGGCGAACTGGAAGATGG
GAATCGCACATCTGGTACTTAATTCTACATTGCACTAAACCATATTCATTTGTATATGATTAAGTGATGAATTGAGGAAA
AATTGACTTACAAAAATAGGAAATTTATTACTATAGGAAATTAATTATAGGAAGGGCAGCCTTGGTGCAACTGGTAAAGT
TGTCTTTATGTGACCTAGATAGGTCACGGTTCAAGCCGTAAAAACAGCTAATGATGCTTGCTTGCAGTAGGCTGCGTATA
TCACACCTCTTGAGGTGCGGCTTTTTCCGGACCTTGCGTACATCACATCCCTTTGGGGGTGTGGCCCTTCCTCCGACCTT
GCGTGAGCAGAATGCTTTGTGTCCCTGACTCTTTTTTTTTTTAGATGTTAATTTAGAATTAACTATGATTATTAAGTGAT
AAACCTTATGAAAATTATTAATTATATTGTAAACCTAGATAAAATTTATAACTTATCATTATTAAGGGATTTAATGCGCT
CAGTATCTATATTAATTAACAACTATGATTAAGTGAGAAAATAACATGCATCTATTAATTTTGTGTAATGATGCGGATGA
GTTTGTTTTCACTTTGATTTAAATCCTTTATTAATTAATCATCATCATGTGATAAATTGTGTATAAAACTTAAATCATAT
ACTATACTACGGTTTAGTGATAAGCCTTCTGCAAGATATTAGTTGTTGTTTCGATGAATGTACCAGGTGGGGAAAGTATT
TCCAGTGCAGGTGCATACATGCATACCTTACTATAGGCCTTATGAATATAGTTATACCTACAAATCTCTCTTTTCTGTTC
ATCTGTGGGTATGTATGTAGTTGTGCATTTATGCACAAAATTTCATATAGCCGACCCATTGTTTGGGATTGAGGCTTAGT
TGTTGTTGTTACGAGGGGGATTAGTAAGAATTTGTGAAGTCGCATAACGATGGACCATCAAATTCCAACCATTTTCAAGT
AATAAGGTACAAAAAAATGAAGCATGAAGTCCAACTTGACCTTCAGTACGTCTGTACTTTATTTGCCTTTTTATTTAATG
ATGCTTCATTTTATGATGATTTGGATCCCATAGGTTCTATGTGGTATTCTCATTGGCCTTTTTCATGATTACTTATTGGG
GATTTTACATTCTATGTGCAGGGATTGTGGGAAACAAGTGTATTTGGGTAAGTGTCTATCTAAGCTTAAAGTTAATTGAA
GACAGTTTTGCATATTGTTACTGTTCTGTTAATTGTATAAGGGATTAAACCTTTACTGGGATGTTGGTTTTTCAGGAGGA
TTTGATACAGCTCATGCTGCAGCTAGGTAAGATTTTATTCTCAGACCTAACAATCAATTTTGTTATGCTTAATTAATCCT
TATCATGCAGTATATCTATATAATTTCTTTATTCTTTTGAATGATATAGGGCGTATGATCGAGCTGCAATTAAGTTTCGG
GGAGTTGATGCTGACATCAATTTCAGTATAACTGATTATGAAGAAGATATGAAGCAGGTAGAATAATGTGGTGGAATGCA
ACCAATAACTGTGGATATCATGAGAGTTAATTAATTTGAAAGTTGACCTTTAATAAATTGATTTGCAGATGAAAAACTTG
TCAAAAGAAGAATTTGTCCAAATCCTTCGTCGTCAAAGCACTGGATTCTCTAGGGGAAATTCAAAATTCAGAGGGGTAAC
CTTGCAGAAATGTGGTCGATGGGAGGGTCGCATGGGTCAATTCCTTGGGAAAAAGTAAGGATGCCCCAAAACTAGCAGTT
TAAAAGTATCAATGCATGTAACTTGTATTTCTAATTTGGTTTTGAAGGCCTGGTGTTTCAGTCGCTTTTTGTTTCAAATT
CTTGCCTAGATTACAAACTCATGGTAGTTAATTATGTGCGAAAAGTTGATAGGACCCTAGTTTGTAATCTTGCTCCTACA
AGATTACAATGCTTGGCTGCAGGTATATATATCTTGGATTATTTGACAGCGAAGTTGAAGCTGCAATATCCTTGTGATCA
TGAGTTCATACCCTTTTGAGACTAATTGTCTCACCTTTAGCATTCTCATTTTCTGAATTACATCCAACCAAATATGCATT
TTCCAATTTTGGTGCAGGGATTATGACAAAGCAGCCATCACATGTAATGGAAGGGAAGCAATTTCCAACTTTCAACCTAA
CACGTATGGACGGGAAATAAAGGAGGATAATAAAGATGGTGGTAAATCCATCTAACCCTTATCTCTTATTCTTGCAACTT
CTGTGGTGGCCATTTGGAAACCTACAGAAAATAATAATTTCTCTGAACATGGATGTAGGTAGTGGTGAGAATCTTGATTT
GAACCTTTGGATTGCTCCACCTTGGGAGGGGCCAAAGGGCGATGAAATTGGTAGAAACGTGCACTTCAAATTTGGAGCCG
GTGAAATGGTTATTGGAAAAAGACTCGAGGTATTTTCTGATTCAAATACTTAACAACTTCTGTTTTAGTGAGGCCTCCAT
TATGGCAAATTCTGTGCTCTCTCTCTCCCCTCCTCTCTCCCTTTCTCTCGGCTTGAGCATTTTGGGTCTGATCCATTCAG
ATGTTAGTAACAGTTATGGCTACTCTTTGCTCTGTCAAATTGTTTTATGAACACATACAGTAGTCTAGACATTTTGAAGT
TATTGCTGAATCTATAGCATGATTTTGCATTCTCTTATTTTGTCAATCTTCGTTCTTTTTTAAGTTTCCTTTTATGAACT
CTGCTTTTGCCAATCTTACAGATTGAAAGCTCCTCTACTGCTCCACAAGGTCCACCAACAGCATCTAAGTGTTCCATACT
GACTGGAATGTATCCTGGTTTTTCTCCACATAATTGGGTAAGTATTATGTTCTTCTCCCGATATAAGTAAATTTATGTTA
TAGTGTCGAGCGTAACTGTTATATGAAATAACACCAGGCAATGCTTACTTCTGCCATCTGCTAAGTTAAGATTTTGAAAG
AATGGAGAAGCAACTTGTTTAATAATAGACTTGTAAACTCCATTATGGTACACATGGGATTTGTAAACTAAGCAGACTTT
TGTTTAGCTATCCGCCCCACAATTATTCTCCCCTTGCCCCACACCCCTTCTCAACAATATTTCCTCTTCGAATTTATTAC
TCCACCAAGCATAATAATTTCAAGACTGTTTCTTGCACATAGTAACATTTATATTATTCTGGTCCAGAAACTACTGAGGG
CACTACAGTATGACCCTTAAACAAAATATTTCCTTGTCTCCTTGATTCATTATCAACGATGATTGACATGGATTTAATGT
TTTTGGGGATACCATATGATTGTTATGCTTTCAGCGCTCCAACTTTTATGCTGGAAATACTTACATTGACAGAGATGAAA
TTATTTCATCATTCTTTCAAATTACTTCATTTTTTATTTGAACAAAACCTGATGGAGATATTTCTTATTGTTAACAGGAA
GGAGGAATGACGAAGGCTGAAGCAACTTCCTCTCCAGGATTCCCAAATTGGGCATGGAAATTTCGTAGCCAGGGTATGGC
CACTCCAGTGCCAGTGTTTTCTTCTTCTGCAGCATCATCAGGATTCTCTACCACTACCACTCTCTATCCAAATTCCCTCC
TACCGCCAAGCAACCAACTACGACTCCCCACAAACTCGACTTTCCCACACCCTCCAATGACCCTTAACATCAGTTAA

>*DL-162Scf01024g00326.1_2* 13841165
ATGTTGGATCTGAATGTATCAGCAGTTTCTGTGAACTCAAATTGTGATGAAACTGACCCATACAACAATACCAACACGTT
CTTGAAAGATGATATTTCTGGAACTTCAAACACTGATTCTTCTTCAGTCGTCAACGCTGTTGTGGGAGATGAAGATTCCA
ACAGCTCTTCACATCAGCATGTGTTTAAACATTCTCTTTCAACTCTGAGTTTCTCCATATTGAAAAGTGATCGTGTTATG
GAAACTGAAGATGACATGACCAGTGATGATTATAAAACAAGGCAGCTTTTTCCAGTGAAAGTGGAGACGAAGATTCAAGA
TCAAGCTCAGTGCTGGCTTAACTTGTCTGTGCCGGAATCCCGGGGAGGGGCAGATATCGGAGTATACAAACCACCGCCGG
CCAAGAAAAGCAGACGGGGGCCTAGGTCCCGTAGCTCACAGTATCGTGGTGTCACATTTTATCGGCGAACTGGAAGATGG
GAATCGCACATCTGGTACTTAATTCTACATTGCACTAAACCATATTCATTTGTATATGATTAAGTGATGAATTGAGGAAA
AATTGACTTACAAAAATAGGAAATTTATTACTATAGGAAATTAATTATAGGAAGGGCAGCCTTGGTGCAACTGGTAAAGT
TGTCTTTATGTGACCTAGATAGGTCACGGTTCAAGCCGTAAAAACAGCTAATGATGCTTGCTTGCAGTAGGCTGCGTATA
TCACACCTCTTGAGGTGCGGCTTTTTCCGGACCTTGCGTACATCACATCCCTTTGGGGGTGTGGCCCTTCCTCCGACCTT
GCGTGAGCAGAATGCTTTGTGTCCCTGACTCTTTTTTTTTTTAGATGTTAATTTAGAATTAACTATGATTATTAAGTGAT
AAACCTTATGAAAATTATTAATTATATTGTAAACCTAGATAAAATTTATAACTTATCATTATTAAGGGATTTAATGCGCT
CAGTATCTATATTAATTAACAACTATGATTAAGTGAGAAAATAACATGCATCTATTAATTTTGTGTAATGATGCGGATGA
GTTTGTTTTCACTTTGATTTAAATCCTTTATTAATTAATCATCATCATGTGATAAATTGTGTATAAAACTTAAATCATAT
ACTATACTACGGTTTAGTGATAAGCCTTCTGCAAGATATTAGTTGTTGTTTCGATGAATGTACCAGGTGGGGAAAGTATT
TCCAGTGCAGGTGCATACATGCATACCTTACTATAGGCCTTATGAATATAGTTATACCTACAAATCTCTCTTTTCTGTTC
ATCTGTGGGTATGTATGTAGTTGTGCATTTATGCACAAAATTTCATATAGCCGACCCATTGTTTGGGATTGAGGCTTAGT
TGTTGTTGTTACGAGGGGGATTAGTAAGAATTTGTGAAGTCGCATAACGATGGACCATCAAATTCCAACCATTTTCAAGT

AATAAGGTACAAAAAAATGAAGCATGAAGTCCAACTTGACCTTCAGTACGTCTGTACTTTATTTGCCTTTTTATTTAATG
ATGCTTCATTTTATGATGATTTGGATCCCATAGGTTCTATGTGGTATTCTCATTGGCCTTTTTCATGATTACTTATTGGG
GATTTTACATTCTATGTGCAGGGATTGTGGGAAACAAGTGTATTTGGGTAAGTGTCTATCTAAGCTTAAAGTTAATTGAA
GACAGTTTTGCATATTGTTACTGTTCTGTTAATTGTATAAGGGATTAAACCTTTACTGGGATGTTGGTTTTTCAGGAGGA
TTTGATACAGCTCATGCTGCAGCTAGGTAAGATTTTATTCTCAGACCTAACAATCAATTTTGTTATGCTTAATTAATCCT
TATCATGCAGTATATCTATATAATTTCTTTATTCTTTTGAATGATATAGGGCGTATGATCGAGCTGCAATTAAGTTTCGG
GGAGTTGATGCTGACATCAATTTCAGTATAACTGATTATGAAGAAGATATGAAGCAGGTAGAATAATGTGGTGGAATGCA
ACCAATAACTGTGGATATCATGAGAGTTAATTAATTTGAAAGTTGACCTTTAATAAATTGATTTGCAGATGAAAAACTTG
TCAAAAGAAGAATTTGTCCAAATCCTTCGTCGTCAAAGCACTGGATTCTCTAGGGGAAATTCAAAATTCAGAGGGGTAAC
CTTGCAGAAATGTGGTCGATGGGAGGGTCGCATGGGTCAATTCCTTGGGAAAAAGTAAGGATGCCCCAAAACTAGCAGTT
TAAAAGTATCAATGCATGTAACTTGTATTTCTAATTTGGTTTTGAAGGCCTGGTGTTTCAGTCGCTTTTTGTTTCAAATT
CTTGCCTAGATTACAAACTCATGGTAGTTAATTATGTGCGAAAAGTTGATAGGACCCTAGTTTGTAATCTTGCTCCTACA
AGATTACAATGCTTGGCTGCAGGTATATATATCTTGGATTATTTGACAGCGAAGTTGAAGCTGCAATATCCTTGTGATCA
TGAGTTCATACCCTTTTGAGACTAATTGTCTCACCTTTAGCATTCTCATTTTCTGAATTACATCCAACCAAATATGCATT
TTCCAATTTTGGTGCAGGGATTATGACAAAGCAGCCATCACATGTAATGGAAGGGAAGCAATTTCCAACTTTCAACCTAA
CACGTATGGACGGGAAATAAAGGAGGATAATAAAGATGGTGGTAAATCCATCTAACCCTTATCTCTTATTCTTGCAACTT
CTGTGGTGGCCATTTGGAAACCTACAGAAAATAATAATTTCTCTGAACATGGATGTAGGTAGTGGTGAGAATCTTGATTT
GAACCTTTGGATTGCTCCACCTTGGGAGGGGCCAAAGGGCGATGAAATTGGTAGAAACGTGCACTTCAAATTTGGAGCCG
GTGAAATGGTTATTGGAAAAAGACTCGAGGTATTTTCTGATTCAAATACTTAACAACTTCTGTTTTAGTGAGGCCTCCAT
TATGGCAAATTCTGTGCTCTCTCTCTCCCCTCCTCTCTCCCTTTCTCTCGGCTTGAGCATTTTGGGTCTGATCCATTCAG
ATGTTAGTAACAGTTATGGCTACTCTTTGCTCTGTCAAATTGTTTTATGAACACATACAGTAGTCTAGACATTTTGAAGT
TATTGCTGAATCTATAGCATGATTTTGCATTCTCTTATTTTGTCAATCTTCGTTCTTTTTTAAGTTTCCTTTTATGAACT
CTGCTTTTGCCAATCTTACAGATTGAAAGCTCCTCTACTGCTCCACAAGGTCCACCAACAGCATCTAAGTGTTCCATACT
GACTGGAATGTATCCTGGTTTTTCTCCACATAATTGGGTAAGTATTATGTTCTTCTCCCGATATAAGTAAATTTATGTTA
TAGTGTCGAGCGTAACTGTTATATGAAATAACACCAGGCAATGCTTACTTCTGCCATCTGCTAAGTTAAGATTTTGAAAG
AATGGAGAAGCAACTTGTTTAATAATAGACTTGTAAACTCCATTATGGTACACATGGGATTTGTAAACTAAGCAGACTTT
TGTTTAGCTATCCGCCCCACAATTATTCTCCCCTTGCCCCACACCCCTTCTCAACAATATTTCCTCTTCGAATTTATTAC
TCCACCAAGCATAATAATTTCAAGACTGTTTCTTGCACATAGTAACATTTATATTATTCTGGTCCAGAAACTACTGAGGG
CACTACAGTATGACCCTTAAACAAAATATTTCCTTGTCTCCTTGATTCATTATCAACGATGATTGACATGGATTTAATGT
TTTTGGGGATACCATATGATTGTTATGCTTTCAGCGCTCCAACTTTTATGCTGGAAATACTTACATTGACAGAGATGAAA
TTATTTCATCATTCTTTCAAATTACTTCATTTTTTATTTGAACAAAACCTGATGGAGATATTTCTTATTGTTAACAGGAA
GGAGGAATGACGAAGGCTGAAGCAACTTCCTCTCCAGGATTCCCAAATTGGGCATGGAAATTTCGTAGCCAGGGTATGGC
CACTCCAGTGCCAGTGTTTTCTTCTTCTGCAGCATCATCAGGATTCTCTACCACTACCACTCTCTATCCAAATTCCCTCC
TACCGCCAAGCAACCAACTACGACTCCCCACAAACTCGACTTTCCCACACCCTCCAATGACCCTTAACATCAGTTAA

>*DL-BEN*_(reversed) 13882511
ATGTTTGACCTTAATCTTTGTTTCGAAGATGAAGAAGAATTACAATTCGATAATCATAATAATTCTACAGAAACATCAAA
TAATTCTTCTTCAATTATTAATAATATTGAAACAACAACTACTAGTTCAACTTGTGATGATCATGAGTACATTTCATATT
CTAATAATGAATATAATAATAATAATTCATTTGTTGATTTTCTAAAAACTGATAATAATGATCAGTTTTTAGTAACTAAG
GAATTGTTTCCGTTGAGTAACGGTGGAGAAACGGCGGCGCCGGTGAATGTGTATGGTAATTACGGTGGTACAATGGAGCA
GAGGATTATTGTTCCAGTACAGCAACAGCAACAGCAAGTGAAGAAAAGTAGAAGAGGACCGAGGTCAAAGAGTTCACAAT
ATCGGGGTGTTACTTTTTATCGTCGCACTGGTCGTTGGGAATCTCACATCTGGTGTGTGTTTTTGTGAATTTTTGAGCTT
ATTTTTAGCTTGTTTTTGAGAAATTAACTGTTGTTTTTGTAACGTATTTTATGCAGGGATTGTGGAAAACAAGTTTATCT
AGGTAAGGGAATGTGTTTGTTAGTATGTGAGTTTAAGTTCTACGCACTGACGATGTAAATGTTGTTTATACAAATGATTT
ACACTGACTGTATAGGTAAGTTCAACTCGTAAATCGATGTGTATGAATAACTAATATTGTCAGTGTATACAACTTAAGTC
GATGTGTCTATTATGTGTGTTTTAAGTTCAATTCACTGACGATGTAAAAATTTGTATGAAAATGATATGCACAGTCAGTT
AAACTCATAATATCTCTTAATAAATATTACTAATTGGTAATCTAAGTAGTAAAAAAGGCACGTAATATGTTATTACAAGT
TAAAATTCGATGATAGTGTAAAAACATCTTTACATTGTTGATATATAACTTAAATTCGATGTGTTTATTATATGCTGTTT
TGTTGATTTAAGAGTTTATGATTTGTTTCTAACTCTAAGCTTAATTTGGACATTTTTTGGACTTGGTTTTAATCTTGTTA
CAGGGGGATTTGATACTGCACATGCTGCTGCTAGGTATGAAATCAGTTAAACTGAGTTTTGTTAGTGAAGTAATTTTATT
ATTGGAGTTGTTATATGGCAGATATAGGTCGTTATTAGTACTAAATTTCGATACTAATATCAGGGCGTATGATCGTGCTG
CGATTAAATTTAGAGGACTTGATGCAGATATTAATTTCAATGTCAGTGATTATCAGGATGATCTAAAGCAGGTTTGTTGG
ATACTAGCTAATTTAATGAACTACTTAAGTTTGAAATATTGTTTTTTCTCGATTAGTAAACTGAATTTAGTCCATTTCGT
TGTTGTCTTGTGACTGGTATGTAGATGATGAACTTTACTAAGGAAGAGTTTGTGCATATACTTCGACGTCAAAGCACTGG
ATTCTCTAGAGGAAGTTCCAAATACAGGGGAGTCACATTGCACAAATGTGGACGATGGGAAGCTCGGATGGGGCAGTTTC
TTGGAAAGAAGTGAGTTGCCTGAGAACCTTGTCTCACCATTGCTTAGGAATTGTGATGTTATCAGTAATATAGCTGTTTA
AAAGCTTATTGGTATATGATTGTTTCATAATACTGAGTTTGTAAGTTAAAGGGAATATTGTCTCATTGGATATATGACTG
CAGGTATATCTATCTTGGACTGTTTGACAGTGAGATAGAAGCTGCAAGGTCCAATGATCATAACTTAACTCTCCCCTCCG
TTTTACATTATGTGGTTTATCCTTTCAACATTGACTGGACTTGGTCGTTTGCGCTTTCTCAGGGCGTATGACAAGGCTGC
AATAAAATGCAATGGAAGGGAGGCAGTCACCAATTTTGAGCTAAGTACGTATGAAGGGGAATTGAGTACTGAGGCTGATA
ATGGAGGTACTATGTTTAGGCTTTTGTACATATTTCGATTCCATTATGATAGTTGCTTCCGTGTTGAACGATCTGTTTTT
GTATCCTTGTGTAGGTGCAAGCCACAATCTTGATCTGAACTTGGGAATAGCCTCCTCTTCTATAGCTGATGACCAGCATG
ACAACACCTGCCTAATTGGGAATTCTGAATTCCAATGTGCCTCAATTGGTTTGCCTGAATATCGTGGAGCCATGGTAACT
TGGAAGTCCATGATTTTCATTCACATTCGTTCAAGTCATCTTACTTGAACATCTTTCTGCAAAGGAAGTTTGGTAGTTTG
TATTTTCATAAATAAGCAAGCATTCTTAGTTGCACATTTTTTTTGCAGAACTCTCCTTGTACCACAATGGGAAGTAAAAT
GCCTCATGGCCGTCACCTGCTCTGGAATGGAGTAAATACCAGTGTCTTTCCCACATTTAAGGTATTCTTTGTCAAAATTT
GTTGAACCATTTTAAAGTTTTATTGAATTGTAACTGTATCACTCACTGCCAAGCCTATGTCCCAGTTATACATAGTGCTT

ATTTTAGTTTAAAGTGTAATCAGAAACAAATAATGTTCCTTAGCTAACTAGTTAGGAAGCTAATGGTATCTCTGTCTAAC
CTTGGTTCCTTATCTACTTAAATTACAGTCTTCAGTTATCATTCATAACTTTATTAAGATTTTTGCCAGCATATGTGTAG
CATATATGTCTTAATATCCAGTTCTCTTGATGTGATAGCTTTAAGTTGTCTTATGTAACTGCTTCTTATGGGCCTGAAAG
TTCAAGAAGTTTGAGAATTTGATCTTCAGTTGAGCATTGAATGACATATTACAATGCTCCTTCAGGGAACAGCAATAGGG
AAGGGTATGGAAGTTGATTCCGCACCAAATTGGACGTGGCAAGACCAAAATCTTTATGGTGGGAGTTCTTCAGTGTCACT
CTTCTCTACTGCAGCATCATCAGGATTCGCTAATTCAACTACAGCTGCTGCTCATCAACCTTACTTTTCTACTGGACCAT
TACCTTACCATCATTCACCATCACTTGCCAATATGAACTTTGCACAATATTACTGCAGAAGCTGA

>*DL-BOB*(downstream)__(reversed) 13938024
GACACCTTATCCAAATGGATGCGGCAAGACCAGAATTCTTATGGTGGAAGTCCTACAGCTCCATTCTTCTCTACTGCAGC
ATCATCAGGATTCGTAAATTCAGCAATCAGTGGACCTTCAGCTGTTGTTCATCAACTGCACTTTCCTAGTAGAGCACTAC
CTTACCACTATTCACCATCCCTCACAAATGAAACCTTCACATTATTATTGCAGGAGCTGAAATAG

>*DL-BOB*(upstream) 13893052
ATGTTTGATCTTAATCTTAGTTGTGATGATGTTCCAGAAGAATCTGATCAGCTCAATGAACTTTCAGTTACTCAAATGGA
GAATTCAAGAACTTCCAGTACTATTGGTGATGATGATAATTCATGTTCAGATCATATGTCAAATTGTTATACATTTGATA
TACTGAAAGCCAATAGGAAAGAAAGTGAAAATCTTGGTAGTTTTGTGACAAAAGAGTTGTTACCATTGAGTTTGAATGAT
CAGAGCATGGTTTTAGCTCAACAGCAGAGGTTACAGGTGATCAAGAAGAGTAGAAGAGGACCAAGGTCTCGAAGTTCACA
ATATCGTGGAGTTACATTCTATCGAAGAACTGGAAGATGGGAATCTCATATATGGTTAGTGATCATCCCTTGTACTTTGA
TTTTTTAGCAATATTATAGTTGGTTTGATCCCTCAAGCTTTGTAGTACTAGCAACTCTTTAGTTCATTTCCATTCATAGT
GTATACGAGTGTAACTTATTGGGAGATTTATGTTAATGTGTAAAAATCAACCAATTAGAGTAAGACCTCTCTATAATGCT
GTTGTTTGTCTGGAAAACTTTTAGCTGCTATAGTGAGGTTTATAGAGAACATATTATATAACATAAGATGAGAAAGCGGT
TCCAGAGTAATTGTTATAGTGAGGTGGTGTAATAGCGGATGACTACTAAAGAGAGGTCTGACTGAACACGCAATCTCAAA
CTTCTTGAGCTGGGTATAGAAGATCTGTTGATTTTTTACTATTTTTATGCTAGTCGTCAACTCACTGCAACCTTCCTCAC
TTATCCGTGCTTAGTACCACATAGAACTTCATATGCTAGCTCCATACTTAAAAAAAGGGCAGCCCGATAGACGGAGCATC
TCGCGTTCACGTAGGGGCTTGGGAAGGGCTGCACCCGGGGTGCGATGTACGAAGTCTACCATGACACATCAATAGCTGAT
TCAACGGCTCGAACCCCTACTTTACCTTTGATCTAAGGCTCCCTTTACGCTAGCTCCTTGCTTAAGACCAGATAAATTTA
GTCCTATATTGTCCTCTAACATACTGAACTTATGCTTCCTTAGGTGTTGCTTGGAAAAACTTATTGAAAAGTTTAAAATA
GCAGTTGTTTTCTGAGTTTTAGTTGTGACTATCTAACAACTAGTAATGACCTGATTCCACTTTTAAGTTTGGATAGCAAC
TCATAAATTTTTTATATGCGACTGTGAGCAATTTCTTTATGGACTTTGCAGGGATGGTGGGAAACAAGTCTACTTGGGTA
AGCAACTGTGTTAATAACCTCATGTTTTATTGATATGAAAGTTATTCCAGCTTTATTCATGAATTGTTGCTGACTGCAAG
CTTCATTTGGGCATTTGGAACCTGTATTTGCAGGGGGTTTTGACACTGCACATGCTGCTGCTAGGTACTGTATAATTTAA
AATAGGTTTGACCTTAACGCCATGCTGATATTTTGTACTTTTCAAAAGAAAGAATTGTAGTGCTAAGTTTTTTTTTTTTT
TTGGGTACTAATTTCAGGGCATATGACCGTGCTGCGATTAAGTTCCGAGGATTTGATGCAGATATCAATTTTAACGTTAG
TGATTATGAAGAAGATCTGAAGCAGGTTGGTTGAAGTAGCTAATACTGCCATTACTTCTGGTTGAAGTCCCTTTCCAATA
AGGAAATGGCTGCTATGTAATGTAATTGTCTGACTGGTATGTAGATGAAGAACTTTTCCAAAGAAGAGTTTCTGCACATA
CTTCGTCGTCAGAGCACTGGTTTCTCTAGAGGAAGTTCGAAGTTCAGGGGAGTCACTCTGCATAAATGTGGACGATGGGA
AGCGCGGATGGGCCAGCTTCTTGGAAAGAAGTGAGTGACTGCATGTTTCAATGTGGCTTAGAAATGTCACTCTATATTTA
ATGTGCCTATTTCAAGTTTTCCATTATCTATCACATGATAGCACTGATCTTTATAAGCTAGAGGGAATATTGTCTCACCA
GCTACCGTATGTTGTATGCCTGCAGGTATATCTATCTTGGACTATTTGACAGTGAGATAGAAGCTGCAAGGTACAATAAT
CAAAACCAGACTCTTCATTCCCTTTCATGTTATGTAGTTTAACCTCTCAACATTGACTAAACATGGTCTTTGCATTGGGG
CTTGATCAGGGCATATGATAAGGCTGCTATAAAAAGCAATGGGAGAGAAGCAGTCACCAATTTTGAGCTGAGCACATATG
AAGGGGTATTAAGTTCTGAGACTGCTGATACTGGAGGTACTATCATGACTTACACCTTTTTCTGGAAACCATTTTTATGT
TTGTCTCGTTTACTCTTTAGCTTTGGTTAGCTGGTTGAAAACTACTAACCTTTACAATGGATGCAGGCACAAGTCATAAT
CTTGATCTGAGATTGGGCATATCTCCCTCTTCCTGTGCTGACAATCAACATGGAAATACCAGCCAAATGGGAATCTCTCA
GTGCCGGCCTGGCTCAAATGGTTTACCTGAACATAGAGAAGTCTTGGTAATTTGAGACCTCATGTTTCTATCTTCATCCA
TAGTCTTCCATGTATACTAGTGAGATTTGTAACAAAGGTAGATTTGTATTCAACTTCTATGGCTGAGCAGCATACTTGTG
CCTCTCTTGCAGAGCTCTGCTTCTACTACACCAAGAAGTATGCTGCTTCATGGTCAGCATATGCTAGATCAGCACCCCCT
CCATTGGAATGGACCGAATGACAATCTCTTTCCCACATTTAAGGTAACTTCTACATTGCTAGGAATTAGGATTATTTAAT
TTCTGCTCGTAAAAAATGGTGTTTGTAGCCTTGATTACCTAGTTTCATCGACATGTTTCTTATTTATGCTTAATGTAATT
TGGAAGCATCATTGCAGTTACCTCACAGGACAGGGAACTTATATAGTCTCGGCCTAAACCCCACATTTCATGATTTCAAG
AGCATCATCTTATTTATTATTGACATTCTCAATAAAATTTCATCTTGTATATATGTAGTTTATTGAGGTCAGAAAAGATA
CTCTTTTCCTGTATGGTAATTGTGACAAATTATCTAGTAGGAGAACCTTTTAGTTCTATGTAAAAGCCTTATCATGTGCA
TCTTTTAACAACATTCTATTCGCGCTTCCTGCAGGGAACAGCAATAGAGAAGGGCTTGGAAGTTGACACTGTAACAACTA
AATTCGCGTCAAGAAAATAAATATCGAGACAAGAAAATATTGCAACAATATATTTATTTATCAAAA

Table S5. Nucleotide sequence of B-class genes from 'Duo Lavender'.
This table presents the nucleotide sequences of B-class genes identified in 'Duo Lavender' (DL).

>*DL-DEF* 13703682
ATGGCTCGTGGAAAGATCCAGATCAAGAGAATAGAAAACCAAACAAACAGGCAAGTGACATATTCTAAGAGAAGAAATGG
ACTTTTCAAGAAGGCTAATGAACTCACTGTTCTTTGTGATGCCAAAGTTTCCATAATTATGATTTCCAGTACTGGCAAGC
TTCATGAATTCATTAGTCCATCTATCACGTACGTATCCAAGAACTAGTATTCTCTTGTGTATTTCAAATTTGTATTTATG
TTAATTATTTTTGTGTTTTTGATATTGTTTGTATGTTCATAGGACTAAGCAGTTGTTCGATCTGTACCAAAAGACTGTTG
GAGTTGATCTTTGGAACTCCCACTATGAGGTTTTGTGTCGCTTCTAAAGTTTCTAGATTTCCAACTCGATATTTTGTTAA
ATTGTGTGTTAATTCCGTCTTAAACTTCGGTTCAAGTCAAACTAAGACAATCATTTTAGAATCGAGGGAGTATATTTCAG
TGTTTCAGATTAATTATTGTTACTAATATTGCGCAGAAAATGCAAGAGCAACTGAGGAAGCTAAAGGAAGTAAATAGGAA
TCTCCGAAAAGAGATCAGGTAGGTACAAACCTAGGAGTTATAGATAGTATTTACTAGGAATATACTTTATGATATTTTAT
TGATATGTCAAAATAAATGAACAGTATTTTTTTTTAAAATAAAAATTTCTCTTTAAGTTTTGCTGATATTAACAATGGAC
AGGCAGAGGATGGGAGAAAGCCTAAACGATCTGAACTATGAGCAGTTGGAAGAGCTCATGGAAAATGTCGACAATTCTCT
CAAGCTTATTCGTGAAAGAAAGGTTTAATATATTTGCTTTTCAAATTCAGCAATTTGCCAATACCTACTAAATTATCAAA
CTAATTTTTTTAAATTGAATCAACTGCAGTTTGCTTTCTTTTCCTTTGGATCTACTTAAATATTCATGTAGATCAACAAA
AAGAAGAGATATAAAGCAACAAAAGAGCCTAGAAACAATCGGAGTAGCTTACATTCTACTTTGTTAATTACTTTAAATTG
CAAATGGGATACTAGGAAATGTCTTTTCATAAAACTAGACAGGGAAATGTTTGCTATTCCTTTATTTAACTTCAGCATTC
TTCAGATGATATGACAATCCCACCTAATTATAGGTCAAAATTACTCTATTTTCTTGGTTTCCCTCATCCCCTCTCAATTT
CATTTGATAATAGAGTGGCTGGCATATAATTATGATAATTTTACTTTTAAATGCATTTTGGAGTTCACATATGTTGCAAC
TATCAGATCGATATTCAATCATTCGGCTAATTTAGATTCGTTTTGACTATGGTTAGAGTGTTTCATATTAAGAAGTTCTT
TAATCTAGAGCTGATCAAACTCGAAATATTTAGTTAGAGATGAAGGAATTCCAACATTTCACTATACCACTTGTAGTATC
ATAGACTTTACTTCACTAGCACCAAAAAATTCCACTCCCATACATCGATCCATCCTTAGACTATCAAAGATTAAGCAAGCA
AATGAAATAAAAGAATAATTCTATTTCCTTGTTTCTTTCTGTCTTTTTGGAAGATTTATCGAATAGGAGAATTGAATATT
CTTGATATATTTTCATGTCCTTAGCTTGTCTTCCAAAGTTACTCATTTAATTTATGTATTTTGTAAACAATGCCTAACTT
AGTTTCATTTGTGAAAGAACAGTATAAGGTGATTGGCAATCAGATTGAGACATTCAAGAAGAAGGTAAAAACTACTATCA
GTTGGCAGTTTTTGTAGGAGTTACTTCTTGAGGGTGAATTCTTATTTTGGTTGATTGTATATTTGCAGGTCAGGAATGTG
GAAGAAATTCATAGGAATCTCTTGCTTGAATTTGTAAGTGCTTAAACTTATTAATTCCAGCTAACTCACTGTTTTGTTAG
AAGCAAAAGCATCTGCCATTGGATTCATATATATTGTCAAAGGCTAAATAGATCTAGGTGAGGGTTGACCTGTTAATTGA
TTTTTTCTTTATGTGAGAATGATGAAAATGATGTTGTGTACGTTGAGAATGGTTCTGCCCCTAAGATCGTTATAAATTCA
AGTAAGAGCCTTAAATTTCCATAGTTGGAATAATTCTTGAATCAGATAAAATAGAAAAGAAGAATAAAGCAGAAAAAATA
TGTAAGATAAGATAATCAACTACTAAAAACTACTCCATAAGATAGTTCAATTGCTAGGATTATTTTTTATAAGGAGCCCT
TTGTCTTCATAGTTTTATCACTAATCCTGGACTCAAATTAATGGGATCTAACAGCCTCGAATACCAAAGTCCTAAAGTAA
AAAAAAAAAAAAGTTTCGTTTATGTACTCTTAGTAAAAGAGTAAGAAATTTAAGCAAAAAAATTTATAAGGTAGTGCATA
TGGTGGATCTTGGATTTTAAATTTATCGTTACGGACTATAACCCTTCTTGCTTATTGGGCTCTAGATAGATTATTTATTT
TTAGAGTATTTTTTATAAACATAAATACAAAATTTAGACCAAAGTTACTGAACCATTAGTAGACTCGTAGATCTGCGCTT
GATAAGGAATTGATGATTTTGGATATTTGTGCAGGATGCAAGACAAGAGGACCCATATGGGCTAGTTGAACAAGAAGGTG
ACTACAATTCTGTGCTTGGTTTTCCAAATGGAGGGCATCGCATATTAGCCTTACGCCTTCAACCAAACCACCACCAGCCA
AATCATCATCATCATCTTCACAGTGGTGGAGGCTCTGATATCACTACTTTTGCTCTGCTTGAGTAG

>*DL-GLO1*
ATGGGGAGAGGAAAGATAGAGATAAAAAGAATAGAAAACTCAAGCAACAGACAAGTAACTTACTCAAAAAGAAGAAATGG
GATCTTGAAAAAAGCTAAGGAAATTAGTGTTCTTTGTGATGCTCGTGTTTCTGTTATCATTTTTGCTAGCTCTGGCAAGA
TGCATGAGTTCTCTTCTACTTCGTTGGTTGATATTTTGGATCAATATCACAAGCTTACTGGTAGAAGATTGTGGGATGCT
AAGCATGAGAACTTGGACAATGAAATCAACAAAGTCAAGAAAGACAATGACAACATGCAAATTGAACTCAGGCACTTGAA
GGGTGAAGATATCACATCTTTGAACCATAGAGAGCTCATGATATTGGAAGATGCCCTTGAAAATGGACTCACTAGTATTC
GTAACAAACAGAATGAGGTTCTGAGGATGATGAGGAAAAAGACTCAAAGTATGGAGGAGGAGCAAGACCAACTTAATTGC
CAATTGCGCCAACTTGAGATAGCAACCATGAATAGGAATATGGGAGAAATTGGCGAAGTGTTTCACCAGAGGGAGAATCA
TGATTACCAAAACCATATGCCTTTTGCCTTCCGAGTACAACCAATGCAGCCAAATTTGCAGGAGAGGTTGTAA

>*DL-GLO2*_(reversed) 13879477
ATATGAAAATATTACGGTCAAAGTTAAACTCGTTTGACTCTCGAAATCCGAACTATGACAACCATTTTGGAACTGAAGGA
GTATTAATTAATTTATAATCAAGTTTATGTAGGAAATATGTCTTTTCAGGCACCTCAAAGGAGAAGATATCAATTCTTTG
AACCACAAAGAGCTTATGGTTTTGGAAGAAGGCTTAACAAATGGACTTTCTAGTATCAGTGCCAAGCAGGTAATTAAATT
TTCCCCTAAATTTTTATCAGTAATCATGTCTTGCCTTTTAATTACAATTATCAGAAACAGACTCTGATGACCTTTCTTTT
TCTTTTTGTTTCTAATTACTTACTGTTCTACAGTCGGAGATCTTGAGGATGGTCAGGAAAAATGTAAGTGTTTGCAAATA
AAGATACCTGCTTATACATAGGTCTGAATTAATGAATTAATGCATTTGGGCCAAGTACTAATATAGTATTTATTTATTTA
TTTGTGATGAATAATTCAGGATCAAATTCTGGAGGAGGAACACAAGCAACTTCAATATGCTTTGGTAAGTCAATCATACA
TGGTAAAATTTACCTACAGCCGATATTTATTTCACCACCCTTATCCAAAACAATTATTACACAAAATCATGTAAGTTTCA
CCTCAACAACTAAAATTAAAGTGATTATAATTATCATTAAAATTTGGTTAAGTGACAAATTGGGTGTATGCAGCATACAT
GTCTAGCATTCTTTGATTGGCGTAAATAACTGCGAGTTTTCCAACATATTTTTAGTTACGGCTCTGATTTTGATAGTAGT
GATTTTTATGTTCATATTGGCTCTGGGTTGGTTATTCTTATTGTAAATTGGTAAAAATTTCAAGTTTTTATGGGTAAAAA
TCTTAAAATTTTGTTTATTGGAGAATAACTTGACTTATATAATCAACTTTATTAATCTGTGCACAGCACCAAAAGGAGAT
GGCAGCCATGGGTGGAAATATGAGAATGATTGAAGAAGTGTACCATCAAAGAGACAGGGATTACGAATACCAGCAGATGC
CATTTGCCCTTCGAGTTCAGCCAATGCAGCCAAATCTACATGAAAGAATGTAGAGCCTATAATTCTACTATATGCATTTT
AAATGAAAGATCGTTAACAATTTAGGGTATGTACTAGAAGACTTCTAACTAGTGATATGGTGAATTAACTAGACTATTGC

GGGACAACCTTTTCATGTGTGACAATATAATTATAGTATATTACCTAATGGTTGTACTGAATATTGAAGTTCTCTTCACT
TCCTACATGGTTTTAAGTATTGATATGCAATGTTTGTGCTACTATATATAGATATGGCATATTTTGGTTGCATTGTTATT
ACGC
>*DL-TM6*
ATGGGTCGTGGTAAAATTGAGATCAAGAAAATAGAGAACTCAACAAACAGGCAAGTGACTTACTCCAAGCGAAGAAATGG
TTTATTCAAGAAAGCTAAAGAACTTACTGTTCTTTGTGATGCTAAGATCTGTCTCATCATGCTCTCCAGTACTAGGAAAT
TCCATGAGTATACTAGCCCCAACACTACGACAAAAAAGATGATTGATTTGTACCAAAGGACACTTGGGGTTGATATTTGG
AGCAAGCATTACGAGAAAATGCAAGAAAACTTGAACAGATTGAAAGATATCAATAACAAGCTAAGAAGAGAGATAAGGCA
AAGAACAGGAGAAGACATGAGCGGCCTCAATTTGCAGGAATTGTGCCACTTGCAGGGGAACATCTCTGATTCTCTAGCTG
AAATACGTGAAAGAAAGTATCACGTGATCAAGACTCAAACAGATACCTGCAGGAAGAGGGTGAGGAACTTAGAAGAGCAA
CATGGAAGCCTCGTACATGATTTGGAAGCAAAAAGCGAAGATCCAACGTATGGTGTAGTGGAAAATGAGGGACATTTCAA
CTCTGCTATGGCATTTGCCAATGGGGTACACAACCTTTATGCTTTTCGCCTACAGACATTGCACCCCAATCTTCAAAACG
GAGGAGGGTTTGGTTCTCGTGATCTACGTCTTGCTTGA

Table S6. Nucleotide sequence of C-class genes from 'Duo Lavender'.
This table presents the nucleotide sequences of C-class genes identified in 'Duo Lavender' (DL).

>*DL-FBP6* 13542691
TGGGAAAATGTATAATAATACAACAGACCATAAAAAAGAAATAGGATCATACGAGCAAGTTATTTTTTTTGATGATCCAT
GTAAATCAATTATGACAAGCAGGGTAGTGGAAATATAGAGTTGGAAGTTTTAATTTCTAATTTATGATGTTTAAAGATGC
CACACCTCTTGCAGGAGATTGAGATGCAGAATGCCAACATGTACCTTCGAGCAAAGGTCCTATTCTTTTTGTTTACATTT
TTTGTATGAACTTTTTATACTCCATTTTTATGATGCTACTACCATATAGGGAATTAGCTAGGTTGTCTAGCATTTTCTGT
AATCTTAAAAAGTTTTGACTTGCAGGATTTAATAAAAAGTAGTAGTAATACTAATTTTATTTCATGGATATTCTTTCACA
TAGAAACTGAGATATTTCCGATGAATTTTTTGAAGCCAAGTGTATTACTTTGAATAATGTGTGCAGATTGCTGAGGTAGA
GAGAGCAACACAGCAAATGAACTTGATGCCTGGAGGAGGATCTGAATACCAGCAACAGCCCATGAGTAGTACTTCTCAGC
CTTATGATGCTCGCAACTTCCTGCCTGTAAATCTGCTCGAACCTAATCCTCATTACTCTCGTCAGGACCAAACTGCTCTA
CAGCTTGTGTAAGCTTCCCTTCTAACCTGCTTATTGCCACTGCTGGTTCCAAGATGGACATCAGCTTTAAATTTCTGACA
GTACGAAAAATAGCCTCCTAGCTGTTATCATCGATGGTTATACCAAATCTTGTATGTAAAAATAAGGAGTAGAATGTGAT
ATCATATACATTTATAATTATCTATTGTCAATTGTTGAGCTATATAGTTGTTCTATATTGATTAAGGGGGCTCCAAAGGA
GTTTCTAGAGGTCCTCAGCACATTCTTAAGGTTTAGGTTGGATGCCAACAATAGTAATCAAAAATCAACCATTGGTAACT
CCATCTTACAGTGATTAGAGCACACAAAGATTCCTTGTATGATTTGAACTCCAAAATTGGGTTACATATGTGGGCGGTGT
TGAATTATATCTGAATGGCTTCTCAATGAGTGATGCAGACCTAAGGCCACGGTGTGAAGTTTGCTAAAGTTTACATCGGA
TTAAGAAATTGTATCTCATATATAGCCAACCAACTCATCTGCCACACTACTTCCTTGAAACAATGTGGGAGTTGAGCCTT
CAATGCAAGATCATTGTATATTTTCTACATTTTATTGGATATGACAAGATATTTGTAATACTCTCTTTTGGATTATCCAG
AATAGTAGTTAAATGTCTCAACTATTAGGTCCATGATCCTTATCTTTTCTACAGATTCATAGAGCATTGCTTTTGCTTTT
GCTATCAAACTATTTCCTCTTCCTAGTGGTGTAGCATCTTGGTTAAAATTACATAATTTTGATTATTGCTTTTGCCTTGA
ACTGCATATTACTAAATACAACAATAGTTTCTAATTGGAACAGTTATGATGTGATCATTTACTATATTTAGTCTCTTACT
GATTACTTTCCCTTATTGCTGCCTGATGAAATCTATGCAGCTGATGATCTTGGAGGAATCTACTATCCAGTTTTCTGAAT
TTAGCTCAGGGGTCTTCCTTCATCTTCAGAAACGTCCTTCAGTTTTAACCTATAGTTTGTAAATCCTCAGAACTGCTACC
ACTGAGATTTGTATGTTTCTGGTTAAGCATTTAGTTATCTGTTTCGTTAAGGA

>*DL-pMADS3*
ATTAAAAGAAACACTCTTTACTTTATAAATACCTATCCCTTAGTGCAAACTCTCTTCCATTTTCTGCATCTATCCTCTGC
AGATTAATTTGCAAAGGAAGAACTAAAAGCTTCTATCTCTTATTCCATCTCCAAATCTTCTTTTCTTATCAGGTTAGCAA
TTAAACTAAAAACATTTACACACCATGTCAAAAAAATCAATTTGAACCATTTTTTGAATGTAACATAAAATAAGTGGTTG
GATATTGATCAATGCAAGATAGTTTGAGTTAGTATGATTAGGAAAGTATTCACCAAATTCTTTAGCAATCATCACTGATT
GCTTCATTTTAGTTGTATTGAACAATAAAAGGTCATTTTTTCCAAGTTTGTCACATAGTTTTTTTTTTTGTTTTTTTGTG
TTTGATATAGGTGCTGCAATGGAGTTCCAAAGTGATCTAACAAGAGAGATCTCTCCACAAAGGAAACTAGGAAGAGGAAA
GATTGAGATCAAGAGGATCGAAAACACGACAAATCGGCAAGTCACTTTTTGCAAGAGACGCAATGGTTTGCTCAAAAAAG
CCTATGAATTATCTGTGCTCTGTGATGCTGAAGTTGCTTTGATTGTCTTCTCTAGCCGAGGCAGGCTCTATGAGTATGCC
AACAACAGGTAATCTTTTAACAAAAAAAAATTAGAAAGTGTTAATTTCAGAAAGTTTAATCTTTACCTTCTTGAGTCTCT
ACAGCTTTGTCTAGCTAGCTTTACCTTTCTTCTTCATTTCTCACTTCTTCTTTTCTTGCAATTCTGTTTGTCTTCCTTAA
AAGAGAAAATAATCGTGATTGGAAAATTTGCTTGTGTTACAGAATGCTTAGGAAGATCTGAACTTTAAAAGGAGAAAGAT
CATGTTGTTTTGAAGTTTTTAAACTAAGAGTTTTTCCTTCTAACAAAGAATTGACTTTCCTTTCCTTTCTTTTTTTATTG
TTTAATAAGTTTTGTTTGTTTCTATTTTTGTGATCCCAATTTTGTTAAAAGACTTGTAGATGTATAGATCTGTTTGTTGT
AAAAATTTGGGAAGGTACTCATCACAACTCTGAGATCAGCATAGTTTCTTCATTTCAGTTTCTGTTATGTTTTCTCTCTC
TCAAAGATGAAGCAAGAGAGCTGTCGAGTTCTAATTCGCCCATTTAGCCCATCATTAAATTAGAGTTAATATGGGATTTA
GATCTTATAGTAGTCCTTTCACAGCCAATGAAAATTCAGATTAGGGTTTTGTACTTTGTTAATAGCTGTGCATTACTGTT
TTTGTCTTGAACTTTGTTTCTATTGGCAGAGTCAAACTTGCCCTAATTAGGGTTTTTTTTTTTCAGCTAGAAAATTATTG
GTGTTTTTCCAATTCTCTAAATTATTTCTTCTTTAACAATAAAATCTCAGATATGCTTCTCATGTGTAGTGCAAATTCTA
GTGTTTTTTCTTGAAATAAAATGTAGAAATCCAATCAGTGTTCAAAGATCAATTATAGACAAAAGTTGACATTCTTCAAA
GTGTTTCCGTTTTCCCTTTTTCTTTACTTTCCCTTCCTATTTCTTAAAATGTTTTACACCGTTGAGGAAATGGGGTCATC
TATACACTCTACTCTAGCTTAGAGTTTTAAGAACCAGAAATTTGTTGGGGTGGGTGGTTGGTGTAAGTTTTGTCTCAGTT
ACAAGAATCTCTGGTCACGTCATACAAGATTTTACACTTGTTGACAAATTTGAGAAAAGACATATTGTGATGAGGGAATG
GTTTCCACTGAGCTATCTCTTAAATTTCTATCTTCAATTGTGTAAAATTGAAAACACTAGCTCCTTATTTTTCTTTGAGA
TATCTCAGTTTAATGCTTTCACAAAGTTTCTGAAAGTGTTGGAACTTAAACAGCAAAGAAACAGAAGTGGAAGAAAGAGA
AGTACTAAACCAATGAAAATTTTACTCACTTAGCTAAGTTTTCAATAGGATCTTGTGCATAGATATATTTGCGTAAGACA
TGTAATAGAGTNNNNNNNNNNNNNNNNNNNNNNNNNNNNAGAGATCAGACATAGATCCGTACAATTTATATTCCAACCAATGA
GAGAGTTCTGAACTCTCTGGCTTTCTATCTTGCATGGTATTGGCTGCTGAAAATGGACGGTTGTGATGTGATCAGATGAG
ACAGACAAGACTGGATCATACTATTCACTTTACCTTACAACACTGTAAAAATATTACTTTATTATAGATTATTGATAAAA
TGCCTTTGAAACAAAACCCTAGGTGGGTAAAAACCTAGAGTTGAATGGTATTACCCAAGGAATAACTGGTACATGATGCG
TGGCATAGATCTGAACCGTTGAAACAGTGGACCAATCATATTGTGGGAACTTGTGCCAGCTTGGCAGAGAGCAGCTAATC
AGTAGCTCGACGAAATTAAGGTTGTAAGTAGACTAGGAAATGAGAGTCATTCCTCAGTGTTGGTTCTTTTCTTTCAATCT
GAAGGTCTATACTAGAAAATATTGTATCAATGTCATGTTTGAGTAGACAGATTGAGTCTGACTCAAAGCAACACTTTGTT
TTTTTATTTAAGCTGTACGTGTTGGCCTAAATTAGCAAGACATAATAGGTGTATTAAATATGGAGTAAGAAAGTATCTGG
ATTTTCATTACCTTTTGAACAAAAGGGTAGTGGTTAAGGCACTCTTTCTATGTTTGCTTCAAGTGAACAAGCACTCGTGT
CTAAAGATATATGAATAGCTGAAACCTCTCTCCTTATGTTTTGGTCCGTAGTATAAGCTTTGTTTCACGCTAAATTAATT
TCTCACGAACTTAGGTACAACACTTTTTGGGGGAAAATGACACTCTGTTTTTAAAGTGTAGTTGTAATAGGAAACGAGAA
AATTTTAAGCTTAAGAGAAGATTAAATTTGAAACTGATATTGTCATGAGCAATTTGACCAGTGTGGGCAAATAATTAATA
CTCTTATCTATTCTATTTTATGTAGGGACGTTTCTTTTTTGTGAATTTTCAAAATAAATAAATAACTTTCTAAATTAAAA

AATAATTTAAATTAAACTTCTATATTAGTAGCACAAATTTGTTGCCAATACTAATCTCACTTTTTTTATTTTGGTAATAT
TTGTAGTACCCCTTTCCCCAATATATATTCTTTTAAATGTTTAACATTACAAGTTTCAAAAAATTTTTGATTGATAGGTT
CCCAACACTCTTCACTTCTGCTTTTAATTCGGTGCTTATCAATACTTTGTCAAATAAATTACGACAAAGGTAATTATACC
TGTTGGCTGATTTACTATCTTTGTGTGAAAATTAGTGAAGTTCAATACTAGTAAATAAAGATCTGGGCAAGCTTGCGTTA
CACATACTTATCATCACATCTTATACAGATCCTTTTTCTTGCCTAAATATTGGAGAGTACAATTAATTATCTTTCTTCAA
TTTCCCATCTTTACTTTGTCAAGCCAACAAACGACTGCAATTTTTTTCTCTTGTTTTCTTGTATTTTCCCCAGTATCTCA
GCGAAGGGTAAATCTTTCATATTGATTTATGCAAATGTGTCATTTTCCTTTTCCTTTTTCTCCTTTCTTTTCAGCCTTTA
CACCATTAAGGAAGACTTAGCAGGTGTTAAGAACACATAGCTAACTACAACTCTACTGTGGGAAATGTGTTTTCACAAGT
TGCTTGTTGACAGCCAAATTGAAGGGAAATGCTCTTTCTTTTGTTATGTAGAATAGCTAAGCACTAGAAACTTATAACCT
ACTAGCAAATGATTTTGTGCAAGCAAATCTTCTTTAACATTATTAATTTTCTTCCTTAATCTTTTAAGTACCCTGAAAGC
TCTAGATTTGCTCGTCTTTCATGTTTCCTAGGGGAGTTGCAAGATGTACGTGGTCATAAGTAATAGCCAAAAATCAAGAA
TACTTTAGCAAATGGAACAAACCGTGGAGATGGCTTTGGAAGATGTTCCTTTTTGTGTTCTTAATTTTTNNNNNNNNNNN
NNNNGTGGGGGTGGTGGGGGCTTAAAATGGAACTTAAATTAATATTTAAATATTTACAAGAGTACAGCGGAAATAGTTAA
TGCATCTATTTCCTCTGTTTAAAAAAAGATGAACCTATTTTTTTTAGTTCGTTTAGATAAAATGATCTCTTTCTAAATTT
TGAAATAAACTTTTACTTTTACCTTTAATGAGATGATATAGCCTACAAATATCTATGACTTACTTTAGACCAAGTTTCAA
AAGTCATTTCTTCTTTATTAAACTCCGCACAAAGTTAAATGGATTCATCATTTTTGAAACAAAGAGAGTAGTGCCATGAG
ACGCTCCTTTTCAAAAACATCAGTAACAAAAGGTTGCGGACTTGCGGTGTGCAAACACTAATTTTGTTGTAAAAAGGGTA
GTTTTCGTGGTAAGAAAATTTGCCTCATTTTGCTGACACCATAGGAGGTCACACAAGAGGGAGGCAAACCTTATTTTGGA
GGAAGGGTCAATAGATAGAATGCTATTAGGAAACTTTTAATTCGATCTTGATTTTGATACCGATCAAAAATATATTAATC
TGGTTGTAACGCGATAGCTGATTATGGTTGTCTATAAATTTTAAGATTGATTAGAAACTGTCTAACATTTTTATGGTCTA
GTGTGAAAGCAACAATTGAGAGGTACAAGAAAGCTTGTTCAGATTCCTCAAACACTGGTTCAATTGCCGAAGCTAATGCT
CAGGTATAATTTCATAAAGTCCTCTTCCTACAACGAAGCCTATATTATGACTTGCTTTGATTATTCAAACTTCTTTGAGA
TTATCGAACCATTTCTTATGTTACGTATTTTGATTCCCCTTCCCCTTCAGTAATGAAATGTTTTATTATATTAGACTTTA
TAGAGATAAAATCTACTACTACCTCATTTCAAAAAAAAAAAATAACACATTTCCTTATTAAACTTTCTATTTTAACACAAG
TATTATGATATATATAAAACCACAAGTTTCAAAAGTTTCATAGTAACATAAATGTTATGGAACCGGGCTTCTATTGTTAT
TCACTCTACTTAATCAGCTGTTCTAAAGTCGTCAAGGTGGCACGATTGGATTAAGTTTTTCATCTAGTATTTTATTTTGA
AAGAATTAGTTTTGTTTTATTTTGGTCGTTTCTTGAAAGAAGGCATTTGTTTTGTTCTTTCTTTCTTTTGGTTCTGAACT
ACTTATGTTAAACCATGTTGATTTGGTTTAGTAAACAACCAAGTTAAACTGAAATACATCTAAGGTCAAATTCTAAGGTC
ATTATAGGAGCATATTCGTATCTCTAGATTCAGTTTACATTTAGTTAATTAAAGGCCTTCTGTCCATACATAAAGTTCTT
AACCAATATGGATTATAAGTTGCAAGCATTATGGATTCTTGATCTCATTAAATCTTATGCTGTTTAATAAAACCCAGTAT
TACCAGCAAGAAGCCTCCAAACTCCGTGCACAAATTGGAAATCTGCAGAACCAGAACAGGTGAAATTTATTGTTTTCTTT
TTTTTTTCTCTTTACTTTTCTATTCACATTTGTTTTCTTACCATTTTTTTCTCATTTTGTTATTTGTTGTTAATGGTCCT
CCTTAACTCAACTACTTCGATTCAGGAACTTTCTTGGTGAATCTCTTGCTGCACTGAATCTCAGAGATCTGAGGAACCTG
GAACAAAAAAATTGAAAAAGGCATTAGCAAAATCCGAGCCAAAAAGGTGTACTTACATTATTTCCCAAAATTTCATATCAC
TTTTGTTTGGTGAAATTTTCAACTCCTTGTGATGGCTATGATTTACTAACATACCATTCTCAAATTACAGAATGAGCTGT
TGTTTGCTGAAATTGAGTATATGCAAAAGAGGGTAAGTAATCGTGCTTACATACCATGAAAGAATAGTTTCCTAAAGTTT
TAAACAGATGTTGAACTTATGTGAAATTCATAAAGATCAAAATGTTCAAGCCTTTTATGTTCATGCCAAAAAGTAACATA
TGTTGATGCACAGCAGGAAATTGATTTACACAACAACAATCAGTATTTAAGAGCAAAGGTCCATTTCTTACTCAAATATT
TGCTTGTTAGTCCTTAAATATATTCCTTCTTCTGCAAAAATTATTGGTTTTTGTATCTAAATTAAACAGATTGCTGAAAC
TGAGAGATCCCAGCAGATGAACTTGATGCCTGGGAGTTCTAGCTATGACCTTGTGCCTCCCCAGCAGTCATTCGATGCGC
GGAACTATCTACAAGTGAATGGCTTGCAGACCAACAACCATTACCCTAGACAAGACCAACCACCTCTTCAACTAGTGTGA
GTACTCCCTCCTACTTAAGTTGCCCAATTATATTATCATTGTTTACTAGTAGTATCATTCAAGTTCTTGCATTGAGCATT
CAGAATTGGTATACTGGATCAAATACTAGATCTAATTTTTCATTAAAATGCCACTCCCTTGTCTCAAATTCTTTTGCACT
CTTTTTGTTCAAGACTGGGAGAGCATTAACAAAACTGTTCAAAATAACTCTTTTATTAAATTGAAATGAATATTTTGTTA
TCTCATAGCGGAAAAAAAAAGGATCTTGAACTTGCAATACTTTTGTACTACTTCTAAATATCTAAATTAGGCATTGTCTAT
ATCGAACTTAATTTTGAAAATTGACCAAGTAGAGACTTGAAAAGCACAAAGAGAAAGAAAAAGTGAAAATTAGGACAGAT
GTATATCATCTAGGAGAGGGGATAGAATGCAACACTCAAAGGTACATTGGAACATTGATATTTATCTTTTTTTACTTAAG
GTGGACACTAAAATATCTTTCTTATATTTTTTCCTTTAGTTTAGGAAATATAGGAATTAATTAAATACTTAATATGCATC
GTTAGATCATCACAAAACGTATTGATACCCAGATCCCCATACTAGCAAAAGTCCAAGTACTGTTCGAATTTTTCAAACAA
AAGTAGTTCCTAATTTGTTTTTCCCTAAAACTGGTAGAACAAATGCAGAAAGAACTGCTTGATAAATAAAACTACAATAG
GCACTGTTTGAATCCTTTCTTAGCTAGTATATCAGCATTCTGCTCTCTGAAACAGTGAAACTACGCTGGATTGCTTGGGC
CGTAGCTGGTGCATCAATAACCTGTAATCATTAACTATAGTCCAATAAGTTTGCTTAGAGTCATTAATAATGTTAATAGA
CTCTGTAGAGTCCACTTCAATATACATAAGAGTGAGTTTGTGCCTACAGGTAACCTTTATTCTTGTTTTCTTGAGCAGCT
AATTTATTTGGAGGTCTTTCTTTCTGCTATGGTCTACATGATCTACAAGAGTAGACTACTAAGCTTGAAGATTCTCGGGA
ATGAAGATCAACTGATGTATACCATATTATTACTTGCTGAATGAAGGAGAATCTTGAATATTATATTTTAAGTGGACATG
TACTTTTCGCTTAATGTTCGTTTTTCTGCCTGTGTACTATCAGGAATTAAGCTTCTTAAGAAGATAACTACTGTTTCGCT
CAATTATGTTCTTAATATTTTTGTGGTAATTTGGATGGTTCATATCACTCCATATATAAGGAAGCCAGGAAGAGATAAAT
GGGATCACT

Table S7. Nucleotide sequence of Phylogenetic analysis.
The evolutionary tree includes sequences from *Arabidopsis thaliana* (*At*), *Eustoma grandiflorum* (*Egra*), *Prunus persica* (*ONH*), *Petunia axillaris* (*Peaxi*) and *Petunia inflata* (*Peinf*).

>peinfROB3
MWNLNDSPDHRKDDELSEEGGKQVRSVSNSSTSSAVDNIEDGTSNSSISISRQKGKEKKTTTTTTHSSKLFGFSVISPNN
NNNNDNLSSESEPPVTRNFFPVDESEIGHATASTFDDGSSRSFPRAHWAGIKLCQPESPGNSSVGKNIEFSQQVQPMKKS
RRGPRSRSSQYRGVTFYRRTGRWESHIWDCGKQVYLGGFDTAHAAARAYDRAAIKFRGVEADINFNLEDYEEDLKQMRNL
TKEEFVHVLRRQSTGFPRGSSKYRGVTLHKCGRWEARMGQLLGKKYVYLGLFDTEVEAARAYDKAAIKCNGKDAVTNFDS
SIYENELNLTESTHKALDHSLDLSLGGSSSKQSSKQLTEDNEDQNYSSIKFDIDRRHQRLRPKQQTSPTRRRDGYNETET
MQLLSKTHLHSPGSLKSNKNEMQRFGQYMRVGHEAQMIQMFSPQFSSSNYQNIQFPSSSNVDRYAGPNTNRREPMFSSSD
TQQWQYSNTVPPQLFATAAASSGFPQQIVRFQNNWSQKNGFNYSLMRPS

>peinfROB2
MWNLNDSPDQRMEEESEEACSSPIDEKGKRVGSVSNSSSSAVVIEDGNNNNNSEDEDEEEEDDEKGKKKRSIHNKIFGFS
VMDPNNYRDLSESEQPVTRNFFPVEESEMGSSTNLPRSQWAGIKFYHESETRGSTVLAGNPTEIVVQQQQPVKKSRRGPR
SRSSQYRGVTFYRRTGRWESHIWDCGKQVYLGGFDTAHAAARAYDRAAIKFRGMTNLTKEEFVHVLRRQSTGFPRGSSKY
RGVTLHKCGRWEARMGQFLGKKAYDKAAIKCNGKDAVTNFDRSIYENELNTTEFTDNAADHNLDLSLGSSSSKEGSRELM
GDNRGQNFQLDVDWRNQGSRPKLPNPIGFDTQRRGGYNESETLQLLSQTHIHSPGSLQPNINEMQRFGQFTRNGESHMVQ
VFPRQFSSYQNQSPSSSNVGQIGTTNARDVLSLTTSNSQEWYHPNIPPHHIFATAAASSGFPQQIVRPQNWSQKTGFHHT
FMRPP

>peinfROB1
MWDLNDSPDQRREINIDESEEGCSSHIELEPDDEKGKRVGSFSTSSSSAIAIDEISEEEDGEKGKKKRSSPSKLFGFSMV
GPGDLEQPITRQFFPVDEAEAETGVVTNGSLNFPRAHWVGVKFYQNEPLGITGVVDVTQQQQQQQQPMKKSRRGPRSRSS
QYRGVTFYRRTGRWESHIWDCGKQVYLGGFDTAHAAARAYDRAAIKFRGVEADINFNLEDYEGDLKQMTNLTKEEFVHVL
RRQSTGFPRGSSKYRGVTLHKCGRWEARMGQFLGKKYVYLGLFDTEVEAARAYDKAAIKCNGKDAVTNFDPSIYENELNS
TESTDSGADHNLDLSLGGSSSKKNNREFGDNRGQNPSSMQFDVDWRHNGLRPEKQTAPIDMDARRRDNGYNESETLQLLS
KTHLHSPVSLKHNNSHQLQRFGQYMRPGESHMIQMFPPQFGSSNYQIQFPSGSNGGRIGATNVGDLSLSSSNASSQWQSN
LPPQIFAAAAASSGFSQQIVRPQNWSSENGFHHSLMRPS

>peinfBOB
MFDLNLSCDDVPEESEQLNELSVAQMENSRTSSTTGDDDNSCSDHMSNCYTFDILKTNRKESENLGSFVTKELLPWSLND
QRIVLAQQQRLQVIKKSRRGPRSRSSQYRGVTFYRRTGRWESHIWDGGKQVYLGGFDTAHAAARAYDRAAIKFRGLDADI
NFYVSDYEEDLKQIKNFSKEEFLHILRRQSTGFSRGSSKFRGVTLHKCGRWEARMGQLLGKKAYDKAAIKSNGREAVTNF
ELSTYEGVLSCETADIGGTSHNLDLRLGISPSSCADNQHGNTSQMGISQCRHGSNGLPEHSEVLSSASTTPRSMLLHGQH
MLDQHPLHWNGPNDNLFPTFKGTAIEKGLEVDTSSKWMRQDQNSYGGSPTAPFFSTAASSGFVNSAFAAPSAVVHQLHFP
SGALRYQYSPSLTNMNLSQYYCRS

>peinfBEN
MFDLNLCFEDEEELQFDNHNNSTETSNNSSSIINNIETTTSSTCDDHDYISYSNNNNNNNSFVDFLKTDNDQFLVTKEL
FPLSNGGETAATVNVYGNYGGSMEQRIIVPVQQQQQQVKKSRRGPRSKSSQYRGVTFYRRTGRWESHIWDCGKQVYLGGF
DTAHAAARAYDRAAIKFRGLDADINFNVSDYQDDLKQMMNFTKEEFVHILRRQSTGFSRGSSKYRGVTLHKCGRWEARMG
QFLGKKAYDKAAIKCNGREAVTNFELSTYEEELSTEPDNGGAIHNLDLNLGIASSSLADNQHGDTCLIGPSEFQCASIGL
PEYRGAMNSPCTTMGSKLPHGRHLLWNGVNTSVFPTFKGTAIGKGMEVDSSPNWTWQDQNLYGGSSSVPLFSTAASSGFA
NSTTAAAHQPCFSTGPLPYHHSPSLANMNFAHYYC

>Peinf101Scf12294g00004.1
MKKVMLDLNVSIINNYILDQNLPQVSPSSGTSNSSIQNAEATSSVYDTCSTRAGNLFVTRQLFPVESNREQDITRSDRVN
FTSGFGNVVIVQQQQQQEQQQQAQLKKKSRRGPRSRSSQYRGVTFYRRTGGFDTADVAARAYDRAAIKFRGVDADINFSI
TDYEEDMKQLKHLGKEEFVHVLRRQSNGFSRGSSKFRGVTLHKCGRWEARMGQFLGKKAYDKAAIKTNGREAVTNFEPSA
YEGETISEPQSEGSHHNLDLNLGMSTSSSKENDRFGGKYYHPYDMQDVAKSKMDKPGPVVVGSSYLKGLPMRQPHLWTGA
YSSSSPGYEGRATDTRKDIGSSQGPSNWALQMPGQGGTAPMTMFYTAASSGFLTPATASASPMVPSTIASQYYYHISSHI
PPP

>Peinf101Scf01705g00017.1
MLDLNVSAVSVNSNCDETDPYNNTNTFLKDDISGTSNTDSSSVVNAVVGDEDSNSSSHQHVFKHSLSTLSFSILKSDRVM
ETEDDMTSDDYKTRQLFPVKMEAKIQDQAQCWLNLSVPESRCGADIGTYKPPPAKKSRRGPRSRSSQYRGVTFYRRTGRW
ESHIWDCGKQVYLGGFDTAHAAARAYDRAAIKFRGVDADINFSITDYEEDMKQMKNLSKEEFVQILRRQSTGFSRGNSKF
RGVTLQKCGRWEGRMGQLLGKKAYDKAAITCNGREAVTNFEPNMYGREIKEDNKDGGSGENLDLNLWIAPPWEGPKGDEI
GRNVHFKFGAGEMVIGKRLEIESSSTAPQGPPTASKCSILTGMYPGFSPHNWEGGMTKAEATSSPGFPNWAWKIRSQGMA
TPVPVFSSSAASSGFSTTLYPNSLLPPSNQLRLPTNSTFPHPPMTLNI

>Peinf101Scf01310g01017.1
MMLDLNLSAIYDEKVGKITVADESGTSNSSLRNAEASSSAGDDDTCSTRAAGDLFAFNFDILKVGGAETSRSCCNNDDEE
VYDENHVRMARPDVVTQQFFPVDIAESNRAHHTSRRPDWVDLSYDPPNTVGLREVGIVHPQQQQYQQQQQPVKKSRRGPR

SRSSQYRGVTFYRRTGRWESHIWDCGKQVYLGGFDTAHAAARAYDRAAIKFRGVDADINFNLSDYDEDMKQMKNLSKEEF
VHMLRRQSTGFSRGSSKYRGVTLHKCGRWEARMGQFLGKKAYDKAAIKCNGREAVTNFEPSTYEGETISDPRSEGSQQDL
DLNLGISTSSAKEIERSGSFQYHPYDMQDATKLQMDKSGPAIVSSSHFKAQSMTSEQAHLWNGVYSNFFPSYEERASGKR
VEVGSSQGPPSWIMQMHGQVGTTPMSMFTAAASSGFSSPATIASASPISGPNPNIHNLSFATYSTASTNTSQYFYQIRPP
LPPP

>Peinf101Scf01034g02005.1
MLDLNVSAVSVNSNCDETDPYNNTNTFLKDDISGTSNTDSSSVVNAVVGDEDSNSSSHQHVFKHSLSTLSFSILKSDRVM
ETEDDMTSDDYKTRQLFPVKMETKIQDQAQCWLNLSVPESRGGADIGTYKPPPAKKSRRGPRSRSSQYRGVTFYRRTGRW
ESHIWDCGKQVYLGGFDTAHAAARAYDRAAIKFRGVDADINFSITDYEEDMKQMKNLSKEEFVQILRRQSTGFSRGNSKF
RGVTLQKCGRWEGRMGQLLGKKAYDKAAITCNGREAISNFQPNTYGREIEDNKDGGSGENLDLNLWIAPPWEGPKGDEIG
RNVHFKFGAGEMVIGKRLEIESSSTAPQGPPTASKCSILTGMYPGFSPHNWEGGMTKAEATSSPGFPNWAWKIRSQGMAT
PVPVFSSSAASSGFSTTLYPNSLLPPSNQLRLPTNSTFPHPPMTLNIS

>PeaxiROB3
MWNLNDSPDHRKDDELSEEGGKQVRSVSNSSTSSAVDNIEDGTSNSSISISRQKGKEKKTTTTTHSNKLFGFSVISPNNN
NNNNDNLSSESEPPVTRNFFPVDQSEIGHATASTFDDRSFPRAHWAGIKLCQPESPGNSSMGKNTELSQQVQPMKKSRRG
PRSRSSQYRGVTFYRRTGRWESHIWDCGKQVYLGGFDTSHAAARAYDRAAIKFRGVEADINFNLEDYEEDLKQAKFVHVL
RRQSTGFPRGSSKYRGVTLHKCGRWEARMGQLLGKKYVYLGLFDTEVEAARAYDKAAIKCNGKDAVTNFDSSIYENELNL
TESTHKALDHSLDLSLGGSRSKQSSKQLTEDNEDQNYSSIQFDIDRRHQRLRPKQPSPTRRRDGYNETETMQLLSKTHLH
SPGSLKSNKNEMQRFGQYMRVGHESQMIQMFPPQFSSSNYQNIQFPSSSNVDRYAGPNTNRREPMFSSSDTQQWQYSNTV
PPQLFATAAASSGFPQQIVGFQNNWSQKNGFNYSLLRPS

>PeaxiROB2
MWNLNDSPDQRMEEESEEACSSPIELDDYKGKGVGSVSNSSSSAVVIEDGNNNNNNSEDEDEEEEDDEKGKKKRSIHNKL
FGFSVMDPNNYRDLSESEQPVTRNFFPVDESEMGSSTNLPRSQWAGIKFYLESETRGSTVLAGNPTEIVVQQQQQQPVKK
SRRGPRSRSSQYRGVTFYRRTGRWESHIWDCGKQVYLGGFDTAHAAARAYDRAAIKFRGMTNLTKEEFVHVLRRQSTGFP
RGSSKYRGVTLHKCGRWEARMGQFLGKKAYDKAAIKCNGKDAVTNFDRSIYENELNTTEFTDNAADHNLDLSLGSSSSKE
GSRELMGDNRGQNFQLDVDWRNQGSRPKLPNQIGFDTQRRGGYNESETLQLLSQTHIHSPGSLQPNINEMQRFGQFTRNG
ESHMVQVFPRQFSSYQNQSPSSSNVGQIGTTNARDVLSLSTSNSQEWYHPNIPPHHHIFATAAASSGFPQQIVRPQNWSQ
KTGFHHTFMRPS

>PeaxiROB1
MWDLNDSPDQRREINIDESEEGCSSHIELEPDDEKGKRVGSFSTSSSSAIAIDEISEEEDGEKGKKKRSSPSKLFGFSMV
GPGDLEQPITRQFFPVDEAEAETGVVTNGSLNFPRAHWVGVKFYQNEPLGITGVVDVTQQQQQQQQPMKKSRRGPRSRSS
QYRGVTFYRRTGRWESHIWDCGKQVYLGGFDTAHAAARAYDRAAIKFRGVEADINFNLEDYEGDLKQMTNLTKEEFVHVL
RRQSTGFPRGSSKYRGVTLHKCGRWEARMGQFLGKKYVYLGLFDTEVEAARAYDKAAIKCNGKDAVTNFDPSIYENELNS
TESTDSGADHNLDLSLGGSSSKKNNREFGDNRGQNPSSMQFDVDWRHNGLRPEKQTAPIDMDARRRDNGYNESETLQLLS
KTHLHSPVSLKHNNSHQLQRFGQYMRPGESHMIQMFPPQFGSSNYQIQFPSGSNGGRIGATNVGDLSLSSSNASSQWQSN
LPPQIFAAAAASSGFSQQIVRPQNWSSENGFHHSLMRPS

>PeaxiBOB
MFDLNLSCDDVPEESDQLNELSVTQMENSRTSSTIGDDDNSCSDHMSNCYTFDILKANRKESENLGSFVTKELLPLSLND
QSMVLAQQQRLQVIKKSRRGPRSRSSQYRGVTFYRRTGRWESHIWDGGKQVYLGGFDTAHAAARAYDRAAIKFRGLDADI
NFNVSDYEEDLKQMKNFSKEEFLHILRRQSTGFSRGSSKFRGVTLHKCGRWEARMGQLLGKKYIYLGLFDSEIEAARAYD
KAAIKSNGREAVTNFELSTYEGVLSSETADTGGTSHNLDLRLGISPSSCADNQHGNTSQMGISQCRPGSNGLPEHREVLS
SASTTPRSMLLHGQHMLDQHPLHWNGPNDNLFPTFKGTAIEKGLEVDTLSKWMRQDQNSYGGSPTAPFFSTAASSGFVNS
AISGPSAVVHQLHFPSRALPYHYSPSLTNETFTLLLQELK

>PeaxiBEN
MFDLNLCFEDEEELQFDNHNNSTETSNNSSSIINNIETTTTSSTCDDHEYISYSNNEYNNNNSFVDFLKTDNNDQFLVTK
ELFPLSNGGETAAPVNVYGNYGGTMEQRIIIPVQQQQQQQQQQVKKSRRGPRSKSSQYRGVTFYRRTGRWESHIWDCGKQ
VYLGGFDTAHAAARAYDRAAIKFRGLDADINFNVSDYQDDLKQMMNFTKEEFVHILRRQSTGFSRGSSQYRGVTLHKCGR
WEARMGQFLGKKAYDKAAIKCNGREAVTNFELSTYEGELSTEADNGGASHNLDLNLGIASSSIADDQHDNTCLIGNSEFQ
CASIGLPEYRGAMNSPCTTMGSKMPHGRHLLWNGVNTSVFPTFKGTAIGKGMEVDSAPNWTWQDQNLYGGSSSVPLFSTA
ASSGFANSTTAAAHQPYFSTGPLPYHHSPSLANMNFAQYYCRS

>Peaxi162Scf01024g00326.1
MLDLNVSAVSVNSNCDETDPYNNTNTFLKDDISGTSNTDSSSVVNAVVGDEDSNSSSHQHVFKHSLSTLSFSILKSDRVM
ETEDDMTSDDYKTRQLFPVKVETKIQDQAQCWLNLSVPESRGGADIGVYKPPPAKKSRRGPRSRSSQYRGVTFYRRTGRW
ESHIWDCGKQVYLGGFDTAHAAARAYDRAAIKFRGVDADINFSITDYEEDMKQMKNLSKEEFVQILRRQSTGFSRGNSKF
RGVTLQKCGRWEGRMGQFLGKKDYDKAAITCNGREAISNFQPNTYGREIKEDNKDGGSGENLDLNLWIAPPWEGPKGDEI
GRNVHFKFGAGEMVIGKRLEIESSSTAPQGPPTASKCSILTGMYPGFSPHNWEGGMTKAEATSSPGFPNWAWKIRSQGMA
TPVPVFSSSAASSGFSTTTTLYPNSLLPPSNQLRLPTNSTFPHPPMTLNIS

>Peaxi162Scf00389g00028.1
MMLDLNLSAIYDEKVGEIAVADESGTSNSSARNAEASSSAGDDDSCSTRAAGDLFAFNFDILKVGGAETSRSFSINNDEE

VYDENHMRMARPDVVTQQFFPVDTAESNRAHHTSRRPDWVDLSYDPPNTLGFREVGIVHPQQQQYQQQQQQQQPVKKSRR
GPRSRSSQYRGVTFYRRTGRWESHIWDCGKQVYLGMYYSGFDTAHAAARAYDRAAIKFRGVDADINFNLSDYDEDMKQMK
NLSKEEFVHMLRRQSTGFSRGSSKYRGVTLHKCGRWEARMGQFLGKKYIYLGLFDSEVEAARAYDKAAIKCNGREAVTNF
EPSTYEGETISDPRSEGSQQNLDLNLGISTSSAKEIERSGGFQYHPYDMQDTTKLQMDKSGPAIVSSSHFKAQPVTSEQA
HLWNGVYSNFFPSYEERASGKRVEVGSSQGPPSWVMQMHGQVGTTPMSMFTAAASSGFSSPATIASASPISGPNPNIHNL
SFATYSTPSTNTSQYFYQIRPPLPPP

>Peaxi162Scf00072g00229.1
MKKVMLDLNVSIINNYILDQNLPQVSPSSGTSNSSIQNAEATSSVDDTCSTRAGTSFVTRQLFPIESNREQDLTRSDRVN
FTSGFGNVVIVQQQQQQEQQQQQAHVKKKSRRGPRSRSSQYRGVTFYRRTGGFDTADVAARAYDRAAIKFRGVDADINFS
ITDYEEDMKQLKHLGKEEFVHVLRRQSNGFSRGSSKFRGVTLHKCGRWEARMGQFLGKKAYDKAAIKTNGREAVTNFEPS
AYEGETISEPQSEGSHHNLDLNLGISTSSSKENDRFGGKYYHPYDTQDVTKSKMDKPGPVVVGSSYLKGLPMRQPHLWTG
AYSSFSPGYEGRASDKSKDIGSSQGPSNWALQMPGQGGTAPMTMFYTAASSGFVTPATASASPMVPSTIASQYYYQISSH
IPPP

>ONI24032
MLDLNVNITLTSDASFDYEKTKDMEVEELPQGSRTQMEDSGTSNSSVVNAEEAPTPSNAGEEDSTNNTTSSFVFDILKKD
KDGLCNTTYYGGAKDQNPSLQFVTRSLFPVTGDGGGGNEAECGLGLSSASSTARPQWLNLSFAESGGQAQAELRIMQQKK
PQPRKSRRGPRSRSSQYRGVTFYRRTGRWESHIWDCGKQVYLGGFDTAHSAARAYDRAAIKFRGVDADINFTLGDYEEDM
KQLGHLNKEEFVHVLRRQSTGASRGNSKYRGVALPKCGGAGGRWEARMAQFPEKKVFEKEGIKYNTGREAAAVTNFVDPS
IYEGEVVLDASIEGSGHNLDLSLGISQPSSGQKGNGNLGDFQFRYKERPMVNGSAASAAVGQTPHVLTMVAKHPALYSGM
YPGFLQKYEEMDSDHNGAQAVSSPRYTNLAWQVHGNSHSVSPVQVFSIAASSGFPSSMASTAPPAANYFPPNLQGSASAS
YNVGPLPFPTPSTM

>ONI03164_(peach_PET)
MLDLNLSFVCNDVVSSSDNNLLHLPATTSPIQSSASFNSSSNLTATGDDEDLNFLSPNDVAADDNAHCDARTIQLFPLAQ
SVRSSSSSSSSSRKQWLGLSSNSGLEVEPSYYAPAEQIVPLQHKVKKSRRGPRSRSSQYRGVTFYRRTGRWESHIWDCGK
QVYLGGFDTAHAAARAYDRAAIKFRGTEADINFNVSDYEDDIKQMSNFTKEEFVHILRRQSTGFSRGSSKYRGVTLHKCG
RWEARMGQFLGKKAYDKAAIKCNGREAVTNFEPNSYDGQIMSEAHNGGSDKSLDLNLGIAPPLVSELQKNNSNLSSFPVQ
LGCDDIPIHMRTRNENCAPAPMRAQLSHGSMVASEEPPIMSNINSSFFPIQMERATEKRMDVNSFPNWAWQLQGLNGGAT
PMPLFSAAASSGFPSSTATSPPAAVTQLHFPNTTILHHHFSPSTVTNNIPGFYCRS

>ONI02974
MWDLNDSPDQRPRGDESERCSSQKTSADGDEEKGKRVGSVSNSSSSAVVVEDDGSDEEEDDDGPAKLAKTRGGGGGKIFG
FSMAHEESMDGDPPVTVTVTRQFFPVELDSTTEIMGPTRGAVPPAAPPSSSSSSFPRAHWVGVNFGQSDSGSPGKPPAAV
EAAHQPMKKSRRGPRSRSSQYRGVTFYRRTGRWESHIWDCGKQVYLGGFDTAHAAARAYDRAAIKFRGVEADINFSIEDY
EEDLKQMTNLTKEEFVHVLRRQSTGFPRGSSKYRGVTLHKCGRWEARMGQFLGKKYVYLGLFDTEIDAARAYDKAAIKCN
GKEAVTNFDPSIYENELNPSSESSGVNAAEHNLDLSLGSSNSKKNNQAFGSSDHGQNAAMEVQHSASMQLEADWRNQGFR
QKLNLQRDRSREETDAHRRDGYLETEAMQLLLRTNLHSPAPNEMHKYGQFSRRPTVGDTQMPHTFPPHFNSPNNYHHVQF
PSSSEGGRIGSDLSLSMSDHPHQQQWQSGTPTSDIFATAAASSGFPPQIRPSAQNCWLQKSGFHSLTRR

>ONI00480
MLDLNLNVVGSGPNDVESCGTQMDESGTSNSSVVNADASSTNDDSCSTRAARYDAVTTFNFDILKVRGGEDEEDDVVVTK
ELFPVTGGLSNWPGQGQSSASSSLVRKNLMELGFDHGGTGEVRLVQQKQQQPAAPPPQQQQVKKSRRGPRSRSSQYRGVT
FYRRTGRWESHIWDCGKQVYLGGFDTAHAAARAYDRAAIKFRGVDADINYNLSDYEEDLKQMKNLTKEEFVHILRRQSTG
FSRGSSRYRGVTLHKCGRWEARMGQFLGKKYIYLGLFDSEVEAARAYDKAAIKCNGREAVTNFEPSTYEGEMISEAGNED
GDHNLDLNLGISPPSFGNCQKEVEGHLQFHSGPYDGHNGKRMEHNVNATMSDPPFKGLVMTSQHPPLWNGVYPSCFSNQE
RATEKRIALGSQGPPNWAWQMHGQVSATPMPLFSTAASSGFSFSATTPPAAVHPLQPSTPTALNLCFTSPATAAANTSQ

>Egra_v1_Chr33.g43780.t1
MLDLNLIVTNDDEENFPYDDSTTSNSSTVNANAPESTYNAAATEDDTSSTRSDHLFTFNFGILKGNGETNQSGVETRQSF
PVSQINDAALSNRSHLDLQCTVQQQRLPQKHVKKGKRGPASRSSPYRGVTFYRRTGRWESHIWDCGKQIYLGGFDTADAA
ARVYDKAAIMFRGDDAYINFKLADYQDDMKQMKNLNKEQLVQILRRKSSGFSRGSSKFRGVTLHKCGRWEARMGQLPGEK
YIHLGLFDSEIEAASAYDKEAMKCNGREAVTNFGPNTYRSEAISISQAEDAGTNRNLDLNLGISNSSPKENRSMRGMKYK
ANPVQNVELPKEQTERKIEKGSLQGSQRWACQLQNQCLTSPMAVFPRAAASRFSSPVTSSLSASSQLLKTHSPTALTICL
SSFQP

>Egra_v1_Chr30.g44830.t1
MLDLNVDVASAEYSACEETDSSPEAIRIDFQSNGRRRGAGGEGDCSGASNASSVISGGVIVPDESNSSDQTTFKFSILDN
RRHVAEIEDDYDSGQLQTRHLFLVAAEEKLERRRHWPNLAVPEVVGVGEIGMGNVEKGKHVILPSTQSQPSAKKSRRGPR
SRSSQYRGITFYRRTGFTADLVNLDCGKQLYLEGFDTALAAARAYDRAAIKFRGTDADINFQIGDYEEEMKQMNHLTKEE
FVHVLRRRQSAGVSWGSSKYRDTMPKCSQWDAQLSHFLGKKAFDKGALEYSRKEALVDFELCSYG

>Egra_v1_Chr27.g08820.t1
MMVNTAEIKDDYDSGRLQTRHLFPVAAIEELERPRQWLNLAMPVVGVAEIGMGDAEEREHVIQASTQQPPPAKKSRRGPR
SRSSQHRGVTFYRRTGRWESHIWDCGKQVYLGGFDTAHAAARAYDQAAIKFRGVDADINFQIGDYEEDMKQMKDLTKKNL
FIQWEAQMSHFLGKRAFDKAALEYSRKESVVDFELNSYGCEAARSSKDGGRGESLDLNLWISPSKDGHMKTDSSKSFNVH

CLPFDTFESKWLKDMDASASPQDGAMAHNNPAIWRSNYSWFAPTDEERERKSSLQGMTLPAGIPNWPWKVHHHGAIPSVA
AASSGFSSTTRTCHRPL

>Egra_v1_Chr25.g35480.t1
MLDLNLDVASLEYSACEETDSSLEAVDLEIQSNGIRRGGGGGGKGDDSGASNASSVINEESNSSDRTTFRFSILDNGRNN
CNVPEIEDDYDSSRLLTRHLFPLSAGEKVWRPRHWLNLAVPEVVGVAENGMSDVWKEKHVILPSTQYQLPAKKSRRGPRS
RSSQYRGVTFYRRTGRWESHIWDCGKQVYLGGFDTAHAAARSYDRAAIKFRGVDADINFQIGDYKEDMKHMKDLTKEEFV
HILRRRRSAGVSRGSSKYRDTMPKCSQWEAQLSHFLGKKALDKAALEYSRKEAVVGFELSSYGWESIRSSKNEGMGESLD
LNLWISPSTEGQMKNEMSKRFHVHHLPFETLERKRLKNVNNSIAPQDGALEHNNPAVFRGNYSLFAPKDEGGEIKSSLQG
VALPAGLSNWPWKVHHHGMIPLVDAASSGFSSTNTDCHRPLYPLTGKTRPPSALAFGIEQWLLIATGKS

>Egra_v1_Chr23.g30170.t1
MLDLNVDVASAEYSACEETDSNPEAIHINFQSNGRRRGAGGKGSYSGASNASSVISAGVIVPDESNSSDRTTFKFSILDN
RRHVTEIEDDYDSGRLQTRHLFPVAAEEKLEKCRHWLNLAVPEVVGVGEIGMVNLEKGKHVILPSTQLPPPAKKSRRGPR
SRSSQYRGITFYRRTGRWESHIWDCGKQVYLGGFDTALAAARAYDRAAIKFRGTDADINFHIGDYEEEMKQVKDLTKEEF
VHVLRRRQSAGVSRGSSKYRDTMPKCSQWDAQLSHFLGKKAFDKEALECS

>Egra_v1_Chr09.g05400.t1
MELLDLNAIAIENEEQIFMDHESANSNSSVVNGDVNSTGTVDDTCSVRSAGAENLFTFNFGILNVNGGGEAEEEELENVT
DVVSRTGFVTRELFPGSEVRDNITEAFRSWTDPLFVPQRTQPPVKKSRRGPRSRSSQYRGVTFYRRTGRWESHIWDCGKQ
VYLGGFDTDHSAARAYDRAAIKFRGPDADINFNPTDYEEDMKQVKHLSKEEFVHILRRQSTGFSRGSSRYRGVTLHKCGR
WEARMGQLLGKKYMYLGLFNDEIEAARAYDRAATKCIGTKAVTNFEPISYESKVISNLQIEDADSTHYLDLNLGISDNSS
KDIGRGRNIRYRPYEVKNGSIHKEMLTRDRIGVGSSQEAPKWDWQMLGQLRATPTKVISTAASSGFSSPVTRGFWASSQV
PKAKSPTALDLCFSSYQKPSNNVTHGFLRLKPRA

>Egra_v1_000379F.g00200
MESLDLNAIAIEDEQQIFMDYESATSNSSVVNGEVTEANSTGTIDDSCSVRSAGAENLFTFNFGILKVNGEGEAEEEELE
NVKDVGGRAGFVTRELFPAGEVRDNITETRRSFTDLPFVPQRTQVTQPPVKKSRRGPRSRSSQYRGVTFYRRTGRWESHI
WDCGKQIYLGGFDTAHSAARAYDRAAIKFRGPDADINFNLTDYEEDTKQVKHLTKEEFVYILRRQSTGYARGSSKYRGAA
LHKCGRWEARMGQLLGKKDYDQTAIKCSGTEAVANFEPNSYESEVLSNLQIEDSTHDLDLNLGISNNSSKDNGSGRNVRY
RPYEVQNGSIHKIDSSQLRVRPQTSQQTRIWGGMSAANSAAAPNSKEISTRDRIGVSSSQGPPKGGWQMQGQLRTTPMQV
VSTAASSGFSLPVTSGFPASSNVSKAQSPTSLSLCFSSYHKPSTNVTHGFLRLKPRA

>EgAP2d
MEGWLKWRFEVSAASLPQHHFLIPCFLLSPSKATFGEVGTSALIACSTYFAASPAAHFSPEAVSDGVDTESEKILGVSIS
QMENSGTSNSSVVNVDSSSTAGDVDSCFDHQLDVSAFNFGRLKSSTGVNGSNSRGVVISKNLFPTDTREVVAESARTERW
LDLSGSFHGSPKTTIVNQAQQQRQMVKKSRRGPRSRSSQYRGVTFYRRTGRWESHIWDNGKQVYLGGFDTALSAARAYDR
AALKFRGIEADINFNISDYEHDLKQVIHLTKEEFVHILRRQSTGFSRGSSKYRGVTLHKCGRWEARMGQFLGKKYIYLGL
FDSEIDAARAYDKAAIKCNGREAVTNFDPSMYEEEMIMEAGDGVNRDVIDLDLGIAPPGLAGGRSDKGDVGYQKLQCSSE
FELEHDGRQKSKAAMLATPSAPGPSIISQNSASSTGRSPSFFPIHKGTAIGENVGIGSATNWAWQTQHLRRELPLVSFFP
TAALSGFHSSTSTTPTCSAAAPHPPFFLMPSSSPYKLSTSFSNRSTMTNYYHNS

>EgAP2c
MWDLNDSPDQQIDDESEGCSSPVDDNVKGKRVNGSGSNSSPLTVVMEDISDEENNKLVKGIQKRAGTFGFPVSYEDDNCS
SESEPPSEPVGGGVVGKSPDVSQPLKKSRRGPRSRSSQYRGVTFYRRTGRWESHIWDCGKQVYLGGFDTAQSAARAYDRA
AIKFRGVEADINFSLADYEDDLKQMTNLTKEEFVHVCNLGLFDSEVEAARAYDKAAIKCNGKDAVTNFDPSIYENELNSN
GNSAAKQNGQELPTNVHNATNQRFIGDWRHQGLRSKAGPFELDGSSTW

>EgAP2b
MWDLNDSPDQQIDDESEGCSSPVDDNVKGKRVNESGSNSSSSAVVIEDISDEVEGGIQKRAASFGFPVNNYQDNCSSASE
PPVTHQFFPVDTGGVQNFPRAHWFGVKFCQSEPVGGGVVGKSPDVSQPLKKSRRGPRSRSSQYRGVTFYRRTGRWESHIW
DCGKQVYLGGFDTAQSAARAYDRAAIKFRGVEADINFSLADYQDDLKQMTNLTKEEFVHVLRRQSTGFPRGSSKYRGVTL
HKCGRWEARMGQFLGKKYVYLGLFDTEVEAARAYDKAAIKCNGKDAVTNFDPSIYENELNSIESSSNAAEHNLDLSLGSS
AAKQNGQELGSRHQGLRSKAGPFELDGSCTRGDGYNEAETMQLLSQTHLHSPGALNSREMHTYEPFSIAGEPPMFQNFPP
RFGSSTYHPFQFPSSSYGGLTGVNEDLSLGTNNLNRQCNPPMYYGTAAASSGFPQQTFRSRNWPQKNGFYPLMRPS

>EgAP2a
MWDLNDSPDQERDYESESCSVVDGEDLKGKKRVDGSASNSSSSVVVVGDCSEEEAGEKGCRKRHSSKVFGFPVAYNDDNC
SSESETPVTHQFFPVDESDMGSNDGGSDVGLSNFPRAHWVGVKFCQPEPGGGVGKSGEVSQPSKKSRRGPRSRSSQYRGV
TFYRRTGRWESHIWDCGKQVYLGGFDTAHAAARAYDRAAIKFRGIEADINFTLSDYQDDLKQVSNLTKEEFVHVLRRQST
GFPRGSSKYRGVTLHKCGRWEARMGQFLGKKYVYLGLFDTEVEAARAYDKAAIKCNGKDAVTNFDPSIYSNELNSSDSSC
NLDLSLGNSAPIDVGGDHPRRKGYNASEPHMPPQMLPQQNNSSNFYPFSSSSDRGVSGANGGFSVSRSDPNRQWEAEYLY
GTAAASSGFQQQRVTTQNWPQTNGFHILTRPS

>AtTOE3
MWNLNDSPDHHEESDSRGNPVGHVSNGMSQSATWLPFVLPVTRNFFPAQSMEPGVRWSGFNSVGKSDPSGSGRPEEPEIS
PPIKKSRRGPRSRSSQYRGVTFYRRTGRWESHIWDCGKQVYLGGFDTAHAAARAYDRAAIKFRGVDADINFDIEDYLDDL

KQMGNLTKEEFMHVLRRQSTGFPRGSSKYRGVTLHKCGRWESRLGQFLNKKYVYLGLFDTEIEAARAYDKAAIKCNGKDA
VTNFDPKVYEEEEDLSSETTRNGHNLGLSLGESSSEEFRLKSDIASIRSRIRDEERLLGSDLSLAMMTTTVRSEKQQSDG
GGNRVVGMAASSGFSPQPSPYRIPRTFHFSRP

>AtTOE2
MLDLNLDVDSTESTQNERDSITVKGVSLNQMDESVTSNSSVVNAEASSCIDGEDELCSTRTVKFQFEILKGGGEEEEEDD
DERSAVMMTKEFFPVAKGMNFMDSSAQSSRSTVDISFQRGKQGGDFIGSGSGGGDASRVMQPPSQPVKKSRRGPRSKSSQ
YRGVTFYRRTGRWESHIWDCGKQVYLGGFDTAHAAARAYDRAAVKFRGLEADINFVIGDYEEDLKQMANLSKEEVVQVLR
RQSSGFSRNNSRYQGVALQKIGGWGAQMEQLHGNMGCDKAAVQWKGREAASLIEPHASRMIPEAANVKLDLNLGISLSLG
DGPKQKDRALRLHHVPNNSVCGRNTMMENHMAAAACDTPFNFLKRGSDHLNNRHALPSAFFSPMERTPEKGLMLRSHQSF
PARTWQGHDQSSGGTAVAATAPPLFSNAASSGFSLSATRPPSSTAIHHPSQPFVNLNQPGLYVIHPSDYISQHQHNLMNR
PQPPP

>AtTOE1
MLDLNLNADSPESTQYGGDSYLDRQTSDNSAGNRVEESGTSTSSVINADGDEDSCSTRAFTLSFDILKVGSSSGGDESPA
ASASVTKEFFPVSGDCGHLRDVEGSSSSRNWIDLSFDRIGDGETKLVTPVPTPAPVPAQVKKSRRGPRSRSSQYRGVTFY
RRTGRWESHIWDCGKQVYLGGFDTAHAAARAYDRAAIKFRGVDADINFTLGDYEEDMKQVQNLSKEEFVHILRRQSTGFS
RGSSKYRGVTLHKCGRWEARMGQFLGKKAYDKAAINTNGREAVTNFEMSSYQNEINSESNNSEIDLNLGISLSTGNAPKQ
NGRLFHFPSNTYETQRGVSLRIDNEYMGKPVNTPLPYGSSDHRLYWNGACPSYNNPAEGRATEKRSEAEGMMSNWGWQRP
GQTSAVRPQPPGPQPPPLFSVAAASSGFSHFRPQPPNDNATRGYFYPHP

>AtSNZ_2
MLDLNLKIFSSYNEDQDRKVPLMISTTGEEESNSSSSSTTDSAARDAFIAFGILKRDDDLVPPPPPPPHKETGDLFPVVA
DARRNIEFSVEDSHWLNLSSLQRNTQKMVKKSRRGPRSRSSQYRGVTFYRRTGRWESHIWDCGKQVYLGGFDTAYAAARA
YDRAAIKFRGLDADINFVVDDYRHDIDKMKNLNKVEFVQTLRRESASFGRGSSKYKGLALQKCTQFKTHDQIHLFQNRGW
DAAAIKYNELGKGEGAMKFGAHIKGNGHNDLELSLGISSSSESIKLTTGDYYKGINRSTMGLYGKQSSIFLPMATMKPLK
TVAASSGFPFISMTSSSSSMSNCFDP

>AtSNZ_1

MLDLNLGILSTHNEDEDCKVPTSIFIQEEDSINPSNDNLSLITFGILKRNVEILPPPPPPPPPPPPSENELSGPGNEWLD
LSSMQRNKQETLVMKKKSRRGPRSRSSHYRGVTFYRRTGRWESHIWDCGKQVYLGGFDTAYTAARAYDRAAIRFRGLQAD
INFIVDDYKQDIEKMKNLSKEEFVQSLRRASASLARGGSKYKNTHMRNDHIHLFQNRGLNAAAAKCNEIRKMEGDIKLGA
HSKGNEHNDLELSLGISSSSKVRILEPADYYMGLNRSVTSLHGKPLPGYLPITEIKPLKTVVASSGFPFITMINPSSLSL
SCFDP

>AtAP2
MWDLNDAPHQTQREEESEEFCYSSPSKRVGSFSNSSSSAVVIEDGSDDDELNRVRPNNPLVTHQFFPEMDSNGGGVASGF
PRAHWFGVKFCQSDLATGSSAGKATNVAAAVVEPAQPLKKSRRGPRSRSSQYRGVTFYRRTGRWESHIWDCGKQVYLGGF
DTAHAAARAYDRAAIKFRGVEADINFNIDDYDDDLKQMTNLTKEEFVHVLRRQSTGFPRGSSKYRGVTLHKCGRWEARMG
QFLGKKYVYLGLFDTEVEAARAYDKAAIKCNGKDAVTNFDPSIYDEELNAESSGNPTTPQDHNLDLSLGNSANSKHKSQD
MRLRMNQQQQDSLHSNEVLGLGQTGMLNHTPNSNHQFPGSSNIGSGGGFSLFPAAENHRFDGRASTNQVLTNAAASSGFS
PHHHNQIFNSTSTPHQNWLQTNGFQPPLMRPS

Table S8. Oligo Sequences Used in this Study

Gene/allele	Forward primer (5′-3′)	Reverse primer (5′-3′)
Linkage Analysis		
PhBOB	AGGACAGGGAACTTATATAGTCTCG	CACTGATTGCTGAATTTACGAATCC
**Gene Expression Analysis**		
GAPDH	ACTTTGTTGGTGACAGCAGGT	TCATACCATGACACAACTTTCACA
BOB	AAATGGATGCGGCAAGAC	TGCTGCAGTAGAGAAGAATGGA
BEN	ACTCTCCTTGTACCACAATGGG	CCATTCCAGAGCAGGTGACG
ROB1	GACAGTGGGGCAGATCACAA	TGAGCTTGAACCACCCAAACT
ROB2	CCTCGACAGTTCAGCTCGTA	GTGAAAGCACATCTCTTGCATT
ROB3	TGGGAAGGATGCAGTCACTA	GCTGTGATCTAATGCCTTATGAGTAG
pMADS3	CTATGAGTATGCCAACAACAGT	GCAGCAAGAGATTCACCAA
FBP6	CGTCTCTATGAATATGCCAACAACAG	TGCTCAATGCCTCTCCAACA

Table S9. Nucleotide sequence of the 10kb insertion in *PhBOB*.

>the upstream 670bp region of the 10kb insertion in *PhBOB*

TTTCTGATTTCAGAGCATCATCTTATTTATTATTGACATTCTCAATAAAATTTCATCTTGTATATATGTAGTTTATTGAGGTCAG
AAAAGATACTCTTTTCCTGTATGGTAATTGTGACAAATTATCTAGTAGGAGAACCTTTTAGTTCTATGTAAAAGCCTTATCAT
GTGCATCTTTTAACAACATTCTATTCGCGCTTCCTGCAGGGAACAGCAATAGAGAAGGGCTTGGAAGTTGACACTGTAACA
ACTAAATTCGCGTCAAAAAAATAAATATCGAGACAAAAAAATATTGCAACAATATATTTTATTTATCAAAATATGAGTGTT
ACAATCTCTATGATTCCTCTGATTCGTCTTTTCAAACTATAACTTCAAGGGCTTTTGAGCTTGATCTTGAAGTTTGGATTTGGA
TTTGGATTTGGCTTTGGATTTGATCTTGATCTTGACTTGATGAGTTCAAGAGCTTGGAGCTTTATCTTGAACTTGGTCTTGATCT
TGAACTTGTACTTGGATTCAAGGGCTTGAAGCTTGATCTTGAATCTTCGTCTTGATATCTAGAACTTGTAGAGAAATATTTGA
GTGCTTGCAGCTTTTGTAGAAATCTCTTGCTTTGATCCACGAGCTCTTTCTTGCTTCTTGTTATGGATTCTTGCTTCTGATTTCTT
TT

>the downstream 652bp region of the 10kb insertion in *PhBOB*(reversed)

TTGTAGAGAAATATTTGAGTGCTTNGCAGCTTTTGTAGAAATNCTCTTGCTTTGATCCACGAGCTCTTTCTTGCTTCTTGTTAT
GGATTCTTGCTTCTGATTTCTTTTATTTTTGAAATGATGGATTTGACCCCTTTATATAGATGTGGGGTGGAGTAGCCACCAAGA
AACCCTGACGGGCCTTGGCCCGTGAACCGGCCAATCAGATTTAAGCAATGACTAGGCCGTATTTGATTGGCCATAACATGTC
ACATGTCCACATGGCGTATTTTCATTGGTCTTGGGGTTTGACTTGGATTGTCACATCATTTGACGTGTGGCATTATCCAATTGG
CTATCTTATTTGACTTGGCGCCACGTCACTTGCCATGTGGCACATTTTTGGGCCTTTAGGAAAACTTATATCCATTGGGCCAT
AAGCTAGTTGGGATAGCCTAGCAAGATTGGACTTTTATTATATTTAAATAACTAGCCCAATTATATTAGCCCATAGCACTTAT
TTGGACCAATATATTTTTAATTTAATATATAGCCATTTTATTTCATGGATTTTGATCCAATAAAATTTACTTGTCTACAGACAC
CTTATCCAAATGGATGCGGCAAGACCAGAATTCTTATGGTGGAAGTCCTACAGCTCCATCTCTCTACT

A

```
CLUSTAL O(1.2.4) multiple sequence alignment


miR172_binding                ------------------------------------------------------------ 0
BW-BOB                        CTTCATATGCTAGCTCCATACTTAAAAAAAGGGCAGCCCGATAGACAGAGCATCTCGCGT 60
DL-BOB_upstream               CTTCATATGCTAGCTCCATACTTAAAAAAAGGGCAGCCCGATAGACGGAGCATCTCGCGT 60
DL-BOB(downstream)_(reversed) ------------------------------------------------------------ 0

miR172_binding                ------------------------------------------------------------ 0
BW-BOB                        TCACACAGGGGCTTGGGAAGGGCTGTGCCCCATGGGGTGCATGTAGGAAGGCTACCCTGG 120
DL-BOB_upstream               TCACGTAGGGGCTTGGGAAGGGCTGCACCCGGGGTGCG--ATGTACGAAGT----CTACC 114
DL-BOB(downstream)_(reversed) ------------------------------------------------------------ 0

miR172_binding                ------------------------------------------------------------ 0
BW-BOB                        TGCAAGCATCAATGGTTGATTCAACGGCTCGAAGTCCAACTTTATCATTGATCTAAGGCT 180
DL-BOB_upstream               ATGACACATCAATAGCTGATTCAACGGCTCGAACCCCTACTTTACCTTTGATCTAAGGCT 174
DL-BOB(downstream)_(reversed) ------------------------------------------------------------ 0

miR172_binding                ------------------------------------------------------------ 0
BW-BOB                        CCCTTTACGCTAGCTCCTTGCTTAAGGCCGGATAAATTTAGTCCTATATTTTCCTCTAAC 240
DL-BOB_upstream               CCCTTTACGCTAGCTCCTTGCTTAAGACCAGATAAATTTAGTCCTATATTGTCCTCTAAC 234
DL-BOB(downstream)_(reversed) ------------------------------------------------------------ 0

miR172_binding                ------------------------------------------------------------ 0
BW-BOB                        ATACTGAACTTATGCTTCCTTAAGTGTTGCTTGGAAAAACTTATTGAAAAGTTTAAAATA 300
DL-BOB_upstream               ATACTGAACTTATGCTTCCTTAGGTGTTGCTTGGAAAAACTTATTGAAAAGTTTAAAATA 294
DL-BOB(downstream)_(reversed) ------------------------------------------------------------ 0

miR172_binding                ------------------------------------------------------------ 0
BW-BOB                        GCAGTTGTTTTTTGAGTTTTAGTTGTGACTATCTAGCAGCTAGTAATGAGCTGATTCCAC 360
DL-BOB_upstream               GCAGTTGTTTTCTGAGTTTTAGTTGTGACTATCTAACAACTAGTAATGACCTGATTCCAC 354
DL-BOB(downstream)_(reversed) ------------------------------------------------------------ 0

miR172_binding                ------------------------------------------------------------ 0
BW-BOB                        TTTTAAGTTTGGATAGCAACTCATAAATTTTTTATATGCAACTCTGAGCAATTTCTTTAT 420
DL-BOB_upstream               TTTTAAGTTTGGATAGCAACTCATAAATTTTTTATATGCGACTGTGAGCAATTTCTTTAT 414
DL-BOB(downstream)_(reversed) ------------------------------------------------------------ 0

miR172_binding                ------------------------------------------------------------ 0
BW-BOB                        GGACTTTGCAGGGATGGTGGGAAACAAGTCTACTTGGGTAAGCAACTGTGTTAATAACCT 480
DL-BOB_upstream               GGACTTTGCAGGGATGGTGGGAAACAAGTCTACTTGGGTAAGCAACTGTGTTAATAACCT 474
DL-BOB(downstream)_(reversed) ------------------------------------------------------------ 0

miR172_binding                ------------------------------------------------------------ 0
BW-BOB                        CATGTTTTATTGATATGAAAGTTATTCCAGCTTTATTCATGAATTGTTGCTGACTGCAAG 540
DL-BOB_upstream               CATGTTTTATTGATATGAAAGTTATTCCAGCTTTATTCATGAATTGTTGCTGACTGCAAG 534
DL-BOB(downstream)_(reversed) ------------------------------------------------------------ 0

miR172_binding                ------------------------------------------------------------ 0
BW-BOB                        CTTCATTTGGGCATTTGGAACCTGTATTTGCAGGGGGTTTTGACACTGCACATGCTGCTG 600
DL-BOB_upstream               CTTCATTTGGGCATTTGGAACCTGTATTTGCAGGGGGTTTTGACACTGCACATGCTGCTG 594
DL-BOB(downstream)_(reversed) ------------------------------------------------------------ 0

miR172_binding                ------------------------------------------------------------ 0
BW-BOB                        CTAGGTACTGTATAATTTAAAATAGGTTTGACCTTAACGCCATGCTGATATTTTGTACTT 660
DL-BOB_upstream               CTAGGTACTGTATAATTTAAAATAGGTTTGACCTTAACGCCATGCTGATATTTTGTACTT 654
DL-BOB(downstream)_(reversed) ------------------------------------------------------------ 0

miR172_binding                ------------------------------------------------------------ 0
BW-BOB                        TTCAAAAGAAAGAATTGTAGTGCTAAGTTTTTTTTTTTTTTTGGGTACTAATTTCAGGGC 720
DL-BOB_upstream               TTCAAAAGAAAGAATTGTAGTGCTAAGTTTTTTTTTTTTTTTGGGTACTAATTTCAGGGC 714
DL-BOB(downstream)_(reversed) ------------------------------------------------------------ 0
```

```
miR172_binding                      ------------------------------------------------------------ 0
BW-BOB                              ATATGACCGTGCTGCGATTAAGTTCCGAGGATTTGATGCAGATATCAATTTTAACGTTAG 780
DL-BOB_upstream                     ATATGACCGTGCTGCGATTAAGTTCCGAGGATTTGATGCAGATATCAATTTTAACGTTAG 774
DL-BOB(downstream)_(reversed)       ------------------------------------------------------------ 0

miR172_binding                      ------------------------------------------------------------ 0
BW-BOB                              TGATTATGAAGAAGATCTGAAGCAGGTTGGTTGAAGTAGCTAATACTGCCATTACTTCTG 840
DL-BOB_upstream                     TGATTATGAAGAAGATCTGAAGCAGGTTGGTTGAAGTAGCTAATACTGCCATTACTTCTG 834
DL-BOB(downstream)_(reversed)       ------------------------------------------------------------ 0

miR172_binding                      ------------------------------------------------------------ 0
BW-BOB                              GTTGAAGTCCCTTTCCAATAAGGAAATGGCTGCTATGTAATGTAATTGTCTGACTGGTAT 900
DL-BOB_upstream                     GTTGAAGTCCCTTTCCAATAAGGAAATGGCTGCTATGTAATGTAATTGTCTGACTGGTAT 894
DL-BOB(downstream)_(reversed)       ------------------------------------------------------------ 0

miR172_binding                      ------------------------------------------------------------ 0
BW-BOB                              GTAGATGAAGAACTTTTCCAAAGAAGAGTTTCTGCACATACTTCGTCGTCAGAGCACTGG 960
DL-BOB_upstream                     GTAGATGAAGAACTTTTCCAAAGAAGAGTTTCTGCACATACTTCGTCGTCAGAGCACTGG 954
DL-BOB(downstream)_(reversed)       ------------------------------------------------------------ 0

miR172_binding                      ------------------------------------------------------------ 0
BW-BOB                              TTTCTCTAGAGGAAGTTCGAAGTTCAGGGGAGTCACTCTGCATAAATGTGGACGATGGGA 1020
DL-BOB_upstream                     TTTCTCTAGAGGAAGTTCGAAGTTCAGGGGAGTCACTCTGCATAAATGTGGACGATGGGA 1014
DL-BOB(downstream)_(reversed)       ------------------------------------------------------------ 0

miR172_binding                      ------------------------------------------------------------ 0
BW-BOB                              AGCGCGGATGGGCCAGCTTCTTGGAAAGAAGTGAGTGACTGCATGTTTCAATGTGGCTTA 1080
DL-BOB_upstream                     AGCGCGGATGGGCCAGCTTCTTGGAAAGAAGTGAGTGACTGCATGTTTCAATGTGGCTTA 1074
DL-BOB(downstream)_(reversed)       ------------------------------------------------------------ 0

miR172_binding                      ------------------------------------------------------------ 0
BW-BOB                              GAAATGTCACTCTATATTTAATGTGCCTATTTCAAGTTTTCCATTATCTATCACATGATA 1140
DL-BOB_upstream                     GAAATGTCACTCTATATTTAATGTGCCTATTTCAAGTTTTCCATTATCTATCACATGATA 1134
DL-BOB(downstream)_(reversed)       ------------------------------------------------------------ 0

miR172_binding                      ------------------------------------------------------------ 0
BW-BOB                              GCACTGATCTTTATAAGCTAGAGGGAATATTGTCTCACCAGCTACCGTATGTTGTATGCC 1200
DL-BOB_upstream                     GCACTGATCTTTATAAGCTAGAGGGAATATTGTCTCACCAGCTACCGTATGTTGTATGCC 1194
DL-BOB(downstream)_(reversed)       ------------------------------------------------------------ 0

miR172_binding                      ------------------------------------------------------------ 0
BW-BOB                              TGCAGGTATATCTATCTTGGACTATTTGACAGTGAGATAGAAGCTGCAAGGTACAATAAT 1260
DL-BOB_upstream                     TGCAGGTATATCTATCTTGGACTATTTGACAGTGAGATAGAAGCTGCAAGGTACAATAAT 1254
DL-BOB(downstream)_(reversed)       ------------------------------------------------------------ 0

miR172_binding                      ------------------------------------------------------------ 0
BW-BOB                              CAAAACCAGACTCTTCATTCCCTTTCATGTTATGTAGTTTAACCTCTCAACATTGACTAA 1320
DL-BOB_upstream                     CAAAACCAGACTCTTCATTCCCTTTCATGTTATGTAGTTTAACCTCTCAACATTGACTAA 1314
DL-BOB(downstream)_(reversed)       ------------------------------------------------------------ 0

miR172_binding                      ------------------------------------------------------------ 0
BW-BOB                              ACATGGTCTTTGCATTGGGGCTTGATCAGGGCATATGATAAGGCTGCTATAAAAAGCAAT 1380
DL-BOB_upstream                     ACATGGTCTTTGCATTGGGGCTTGATCAGGGCATATGATAAGGCTGCTATAAAAAGCAAT 1374
DL-BOB(downstream)_(reversed)       ------------------------------------------------------------ 0

miR172_binding                      ------------------------------------------------------------ 0
BW-BOB                              GGGAGAGAAGCAGTCACCAATTTTGAGCTGAGCACATATGAAGGGGTATTAAGTTCTGAG 1440
DL-BOB_upstream                     GGGAGAGAAGCAGTCACCAATTTTGAGCTGAGCACATATGAAGGGGTATTAAGTTCTGAG 1434
DL-BOB(downstream)_(reversed)       ------------------------------------------------------------ 0

miR172_binding                      ------------------------------------------------------------ 0
BW-BOB                              ACTGCTGATACTGGAGGTACTATCATGACTTACACCTTTTTCTGGAAACCATTTTTATGT 1500
DL-BOB_upstream                     ACTGCTGATACTGGAGGTACTATCATGACTTACACCTTTTTCTGGAAACCATTTTTATGT 1494
DL-BOB(downstream)_(reversed)       ------------------------------------------------------------ 0
```

```
miR172_binding                    ------------------------------------------------------------ 0
BW-BOB                            TTGTCTCGTTTACTCTTTAGCTTTGGTTAGCTGGTTGAAAACTACTAACCTTTACAATGG 1560
DL-BOB_upstream                   TTGTCTCGTTTACTCTTTAGCTTTGGTTAGCTGGTTGAAAACTACTAACCTTTACAATGG 1554
DL-BOB(downstream)_(reversed)     ------------------------------------------------------------ 0

miR172_binding                    ------------------------------------------------------------ 0
BW-BOB                            ATGCAGGCACAAGTCATAATCTTGACCTGAGATTGGGCATATCTCCCTCTTCCTGTGCTG 1620
DL-BOB_upstream                   ATGCAGGCACAAGTCATAATCTTGATCTGAGATTGGGCATATCTCCCTCTTCCTGTGCTG 1614
DL-BOB(downstream)_(reversed)     ------------------------------------------------------------ 0

miR172_binding                    ------------------------------------------------------------ 0
BW-BOB                            ACAATCAACATGGAAATACCAGCCAAATGGGAATCTCTCAGTGCCGGCCTGGCTCAAATG 1680
DL-BOB_upstream                   ACAATCAACATGGAAATACCAGCCAAATGGGAATCTCTCAGTGCCGGCCTGGCTCAAATG 1674
DL-BOB(downstream)_(reversed)     ------------------------------------------------------------ 0

miR172_binding                    ------------------------------------------------------------ 0
BW-BOB                            GTTTACCTGAACATAGAGAAGTCTTGGTAATTTGAGACCTCATGTTTCTATCTTCATCCA 1740
DL-BOB_upstream                   GTTTACCTGAACATAGAGAAGTCTTGGTAATTTGAGACCTCATGTTTCTATCTTCATCCA 1734
DL-BOB(downstream)_(reversed)     ------------------------------------------------------------ 0

miR172_binding                    ------------------------------------------------------------ 0
BW-BOB                            TAGTCTTCCATGTATACTAGTGAGATTTGTAACAAAGGTAGATTTGTATTCAACTTCTAT 1800
DL-BOB_upstream                   TAGTCTTCCATGTATACTAGTGAGATTTGTAACAAAGGTAGATTTGTATTCAACTTCTAT 1794
DL-BOB(downstream)_(reversed)     ------------------------------------------------------------ 0

miR172_binding                    ------------------------------------------------------------ 0
BW-BOB                            GGCTAAGCAGCATACTTGTTGTGCCTCTCTTGCAGAGCTCTGCTTCTACTACACCAAGAA 1860
DL-BOB_upstream                   GGCTGAGCAGCATACT---TGTGCCTCTCTTGCAGAGCTCTGCTTCTACTACACCAAGAA 1851
DL-BOB(downstream)_(reversed)     ------------------------------------------------------------ 0

miR172_binding                    ------------------------------------------------------------ 0
BW-BOB                            GTATGCTGCTTCATGGTCAGCATATGCTAGATCAGCACCCCCTCCATTGGAATGGACCGA 1920
DL-BOB_upstream                   GTATGCTGCTTCATGGTCAGCATATGCTAGATCAGCACCCCCTCCATTGGAATGGACCGA 1911
DL-BOB(downstream)_(reversed)     ------------------------------------------------------------ 0

miR172_binding                    ------------------------------------------------------------ 0
BW-BOB                            ATGACAATCTCTTTCCCACATTTAAGGTAACTTCTACATTGCTAGGAATTAGGATTATTT 1980
DL-BOB_upstream                   ATGACAATCTCTTTCCCACATTTAAGGTAACTTCTACATTGCTAGGAATTAGGATTATTT 1971
DL-BOB(downstream)_(reversed)     ------------------------------------------------------------ 0

miR172_binding                    ------------------------------------------------------------ 0
BW-BOB                            AATTTCTGCTCGTAAAAAATGGTGTTTGTAGCCTTGATTACCTAGTTTCATCGACATGTT 2040
DL-BOB_upstream                   AATTTCTGCTCGTAAAAAATGGTGTTTGTAGCCTTGATTACCTAGTTTCATCGACATGTT 2031
DL-BOB(downstream)_(reversed)     ------------------------------------------------------------ 0

miR172_binding                    ------------------------------------------------------------ 0
BW-BOB                            TCTTATTTATGCTTAATGTAATTTGGAAGCATCATTGCAGTTACCTCACAGGACAGGGAA 2100
DL-BOB_upstream                   TCTTATTTATGCTTAATGTAATTTGGAAGCATCATTGCAGTTACCTCACAGGACAGGGAA 2091
DL-BOB(downstream)_(reversed)     ------------------------------------------------------------ 0

miR172_binding                    ------------------------------------------------------------ 0
BW-BOB                            CTTATATAGTCTCGGCCTAAACCCCACATTTCATGATTTCAAGAGCATCATCTTATTTAT 2160
DL-BOB_upstream                   CTTATATAGTCTCGGCCTAAACCCCACATTTCATGATTTCAAGAGCATCATCTTATTTAT 2151
DL-BOB(downstream)_(reversed)     ------------------------------------------------------------ 0

miR172_binding                    ------------------------------------------------------------ 0
BW-BOB                            TATTGACAATCTCAATAAAATTTCATCTTGTATATATGTAGTTTATTGAGGTCAGAAAAG 2220
DL-BOB_upstream                   TATTGACATTCTCAATAAAATTTCATCTTGTATATATGTAGTTTATTGAGGTCAGAAAAG 2211
DL-BOB(downstream)_(reversed)     ------------------------------------------------------------ 0

miR172_binding                    ------------------------------------------------------------ 0
BW-BOB                            ATTCTCTTTTCCTTCATGGTAATTGTGACAAATTATCTAGTAGGAGAACCTTTTAGTTCT 2280
DL-BOB_upstream                   ATACTCTTTTCCTGTATGGTAATTGTGACAAATTATCTAGTAGGAGAACCTTTTAGTTCT 2271
DL-BOB(downstream)_(reversed)     ------------------------------------------------------------ 0
```

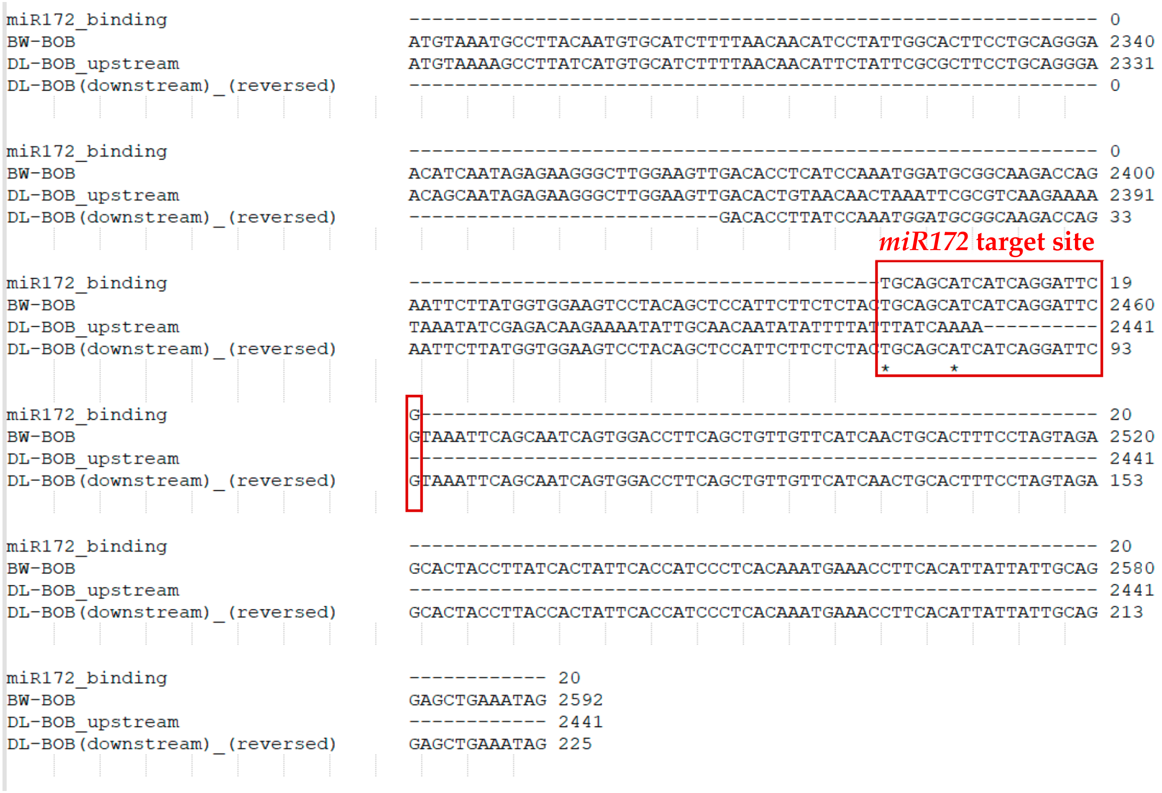

B

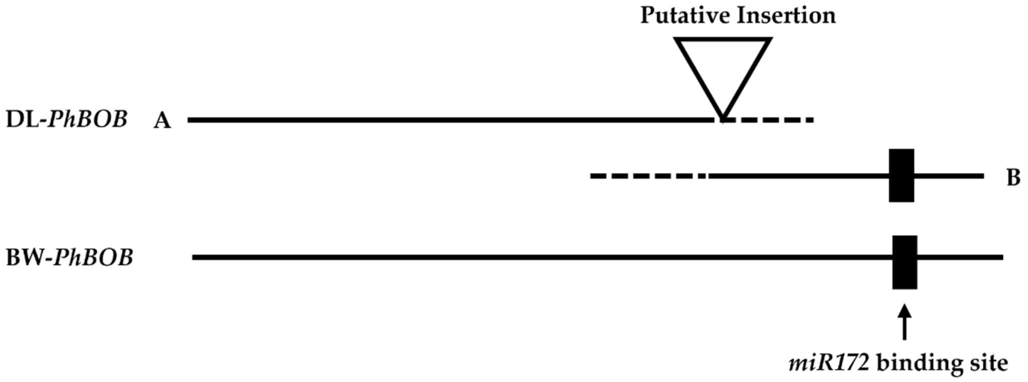

Figure S1. Sequence alignment and schematic illustration of the putative insertion upstream of the *miR172* binding site in *PhBOB*. (A) Nucleotide sequence alignment of the *PhBOB* genomic region surrounding the miR172 binding site in 'Baccarat White' (BW) and 'Duo Lavender' (DL). The miR172 target site is highlighted in red. In DL, we identified two separate fragments corresponding to the *PhBOB* region, which could not be joined into a continuous sequence, indicating the presence of a large insertion between them. (B) Schematic diagram of *PhBOB* gene structure in BW and DL. In BW, the *PhBOB* gene is contiguous with a single *miR172* binding site. In DL, a putative 10 kb insertion is located upstream of the *miR172* binding site, potentially affecting *miR172*-mediated regulation.
